# Supplementary material for: Flexomagnetism and vertically graded Néel temperature of antiferromagnetic Cr2O3 thin films
Source: Nat Commun. 2022 Nov 8;13:6745. doi: 10.1038/s41467-022-34233-5 (PMC9643371; doi:10.1038/s41467-022-34233-5)
Supplement: Supplementary file 2 — supplementary information [file 41467_2022_34233_MOESM2_ESM.pdf]

Supplementary Information

Flexomagnetism and vertically graded Néel temperature of antiferromagnetic Cr_2O_3 thin films

Pavlo Makushko,[1,*] Tobias Kosub,[1,*] Oleksandr V. Pylypovskyi,[1,2,†] Natascha Hedrich,[3] Jiang Li,[1] Alexej Pashkin,[1] Stanislav Avdoshenko,[4] René Hübner,[1] Fabian Ganss,[1] Daniel Wolf,[5] Axel Lubk,[5,6] Maciej Oskar Liedke,[7] Maik Butterling,[7] Andreas Wagner,[7] Kai Wagner,[3] Brendan J. Shields,[3] Paul Lehmann,[3] Igor Veremchuk,[1] Jürgen Fassbender,[1] Patrick Maletinsky,[3] and Denys Makarov[1,‡]

[1] *Helmholtz-Zentrum Dresden-Rossendorf e.V., Institute of Ion Beam Physics and Materials Research, 01328 Dresden, Germany*
[2] *Kyiv Academic University, 03142 Kyiv, Ukraine*
[3] *Department of Physics, University of Basel, 4056 Basel, Switzerland*
[4] *Institute for Solid State Research, Leibniz Institute for Solid State and Materials Research Dresden, 01069 Dresden, Germany*
[5] *Leibniz Institute for Solid State and Materials Research, IFW Dresden, Helmholtzstr. 20, 01069 Dresden, Germany*
[6] *Institute of Solid State and Materials Physics, TU Dresden, 01069 Dresden, Germany*
[7] *Helmholtz-Zentrum Dresden-Rossendorf e.V., Institute of Radiation Physics, 01328 Dresden, Germany*

Supplementary Note 1. Transmission electron microscopy characterization

Transmission electron microscopy (TEM) was applied to determine the mechanism behind the persistent strain in magnetron sputter deposited Cr_2O_3 thin films. The corresponding TEM images indicate high crystallinity and homogeneity of the grown films (Supplementary Figs. 2–6). In contrast to Cr_2O_3 films prepared by thermal evaporation [1, 2], where a clear periodic columnar pattern was observed, there are only shallow fringes in the TEM images of magnetron sputtered films. These shallow fringes come from defects that do not cross the complete thickness of the films.

Dislocations are formed at the free surface during the deposition process, and their nucleation is much easier under compressive strain [3]. Initially, the Cr_2O_3 film grows pseudomorphically on sapphire, reproducing its lattice parameters and accumulating large in-plane compressive strain. The strain energy, which is stored in the film, increases with thickness until reaching a critical thickness, at which it becomes energetically favorable to form dislocations and thereby to relax strain. The nucleated dislocations tend to diffuse towards the interface with the substrate to lower their energy. This process has low dynamics in such hard materials as Cr_2O_3, however, it can be promoted by temperature and by increasing the total thickness of the deposited film. For instance, for the Cr_2O_3 film with the sample thickness of 30 nm, dislocations are observed at the distance of about 5 nm from the interface with sapphire (Fig. 1c, main text), limiting the thickness of the pseudomorphic layer. These dislocations form a phase boundary between pseudomorphic Cr_2O_3 and non-pseudomorphic, but still strained Cr_2O_3. Dislocations tend to propagate towards the film/substrate interface [4]. On the contrary, the 250-nm-thick Cr_2O_3 thin film sample reveals misfit dislocations directly at the interface with sapphire (Supplementary Fig. 2). This mechanism contrasts with the strain relaxation via the grain boundaries in thermally evaporated films.

The gradual color change across the specimen in Supplementary Fig. 3d is a thickness effect due to TEM specimen preparation. In particular, classical TEM lamella preparation, which we applied for the samples discussed in this work, leads to wedge-shaped TEM lamellae, not plane-parallel ones. This means that the TEM lamella thickness is gradually changing. As a consequence, for cross-sectional specimens, the lamella thickness gradually increases from the sample surface (in our case the Pt layer) into the direction of the substrate, but also in the lateral sample direction. More specifically, for the element map shown in Supplementary Fig. 3d, the lamella thickness increases from the upper right side of the image to the lower left side. However, an increasing lamella thickness results in enhanced energy-dispersive X-ray spectroscopy (EDX) signal intensity, thus leading to a gradual color change. For the thinner chromium oxide sample, there is also such gradual lamella thickness change. However, the field of view shown in Supplementary Fig. 6c is significantly smaller than that of Supplementary Fig. 3d. Consequently, the lamella thickness change across this smaller field of view is significantly smaller, leading to a more homogeneous EDX signal intensity and hence a homogeneous color distribution for Supplementary Fig. 6c.

---

* These authors contributed equally
† o.pylypovskyi@hzdr.de
‡ d.makarov@hzdr.de

To characterize the interface between Al_2O_3 and Cr_2O_3 of 30 nm thickness, we acquired aberration-corrected high-resolution high-angle annular dark-field scanning TEM images (HAADF-STEM; Supplementary Fig. 5), exhibiting atomic column contrast roughly proportional to Z^2 (Rutherford cross section with Z being the atomic number). High-resolution HAADF-STEM data has been acquired at 300 kV acceleration voltage at a FEI Titan^3 80-300 TEM equipped with probe corrector. The camera length was chosen such that electrons scattered into angles between 60 mrad and 280 mrad have been detected with the annular dark-field detector. Weakly scattering O columns remain invisible at these large scattering angles. The data reveal an abrupt change between weakly scattering Al ($Z = 12$) and Cr ($Z = 24$), and hence an atomically sharp interface. Moreover, the data confirms epitaxial phase matching between Al_2O_3 and Cr_2O_3 with the same in-plane lattice parameter at the interface.

To quantify the strain distribution along the whole thickness of the Cr_2O_3 film, we conducted a geometric phase analysis [5][6] of large field of view high-resolution TEM data (HR-TEM, Supplementary Fig. 5a). The Fourier transform, shown as inset in Supplementary Fig. 5a, confirms crystallinity of the Cr_2O_3 layer. Supplementary Fig. 5b,c display the diagonal components u_{xx} and u_{zz}, revealing the varying in- and out-of-plane dilation or compression (the shear component u_{xz} was constant throughout the image, Supplementary Fig. 5d). In particular, we observe an almost linearly increasing in-plane strain tensor component u_{xx} as a function of the distance from the interface until bulk lattice parameter, in agreement with the X-ray diffraction (XRD) data. This gradual relaxation of the Cr_2O_3 lattice clamped at the Al_2O_3 interface is facilitated by the presence of misfit dislocations and other crystal defects, appearing as sharp lines of diverging strain in the strain maps (indicated with circles). The out-of-plane lattice parameter of Cr_2O_3, being not clamped, shows a sharp jump at the interface, which is subsequently reduced again in response to the growing in-plane lattice parameter.

For the geometric phase analysis, we selected the two linearly independent systematic reflections $(0\,\bar{1}\,1\,\bar{2})$ and $(0\,\bar{1}\,1\,4)$ for retrieving their geometric phase, and hence displacement field along the corresponding reciprocal lattice direction. Here, the mask sizes utilized for cutting out the reflection had a characteristic radius of $0.5\,\mathrm{nm}^{-1}$, corresponding to $2\,\mathrm{nm}$ spatial resolution of the displacement field maps. Finally, the elastic strain tensor is evaluated utilizing Eq. (S10). Consequently, the ratio between the in- and out-of-plane lattice parameters of Cr_2O_3 (Supplementary Fig. 23e) and the strain tensor (S24) reads $a = (1 + u_{xx})a_0$ and $c = (1 + u_{zz})c_0$. We used the bulk Cr_2O_3 lattice parameters a_0 and c_0 as reference, with respect to which the strain is calculated. To retrieve the averaged strain profiles as function of distance to the Al_2O_3 interface, we deliberately neglected the regions of diverging strain (due to crystal defects) by considering only strain components smaller than 0.1 by the absolute value in the evaluation. The presence of the crystal defects in combination with the numerical derivative is responsible for the large scatter of the strain data.

Regarding the Pt/Cr_2O_3 interface, there is no Cr-enriched layer for the 30-nm and the 250-nm-thick samples, within the resolution of the analysis. According to the STEM-EDX results, we do not see any hint for an O deficiency at the Pt/Cr_2O_3 interface.

Supplementary Note 2. X-ray diffraction studies

We use X-ray diffraction (XRD) to analyze the crystal structure and strain in the sputter deposited Cr_2O_3 thin film samples. In this respect, reciprocal space maps (RSM) study is commonly used for the characterization of thin films, refining lattice parameters, estimating strain and strain distribution [7]. In particular, there are detailed studies where RSM is used to characterize epitaxial thin films of similar space group [7–10]. The penetration depth of Cu-K_α radiation in Cr_2O_3 is on the scale of more than 10 micrometers. Therefore, in our analysis we assume that the intensity of the signal is equal throughout the film thickness of all samples studied in our work.

Two series of RSM investigations were performed on Cr_2O_3 thin film samples. The results of the first experiment series (XRD Series I) are shown in Supplementary Figs. 9,10,11,12,15,16. The results of the second experiment series (XRD Series II) are shown in Supplementary Figs. 13,14,17,18,19,46,47.

Supplementary Figs. 9,11,15 show RSM data of the asymmetric $(1\,0\,\bar{1}\,10)$ and symmetric $(0\,0\,0\,6)$ reflections for Cr_2O_3 thin films thickness of 30 nm, 50 nm and 100 nm, respectively. Two pronounced reflections are displayed on each panel: a sharp reflection from the Al_2O_3 substrate and a broad reflection from the Cr_2O_3 film. The positions of the strain-free reflections for the bulk Al_2O_3 and Cr_2O_3 are indicated with black crosses. The RSM suggest high crystallinity and epitaxial growth of the Cr_2O_3 films on $(0\,0\,0\,1)$ oriented sapphire substrates. The main Cr_2O_3 reflection of the sputter deposited films is shifted substantially from its relaxed position, indicating longer c and shorter a lattice parameters, see Supplementary Figs. 9a,d and 11a,d. The lattice parameters were extracted from the RSM using the following Gaussian fit of the profile of the reflections on q_c and q_a (for RSM maps, we use axes labels q_a and q_c to refer to the lattice parameter assessed from them)

$$I_{\text{XRD}} = \sum_{i=1}^{N} I_i^{\max} \exp \frac{(Q - Q_i^{\text{opt}})^2}{0.5 w_{i,\text{XRD}}^2}, \quad Q = q_a \text{ or } q_c, \tag{S1}$$

where $N = \overline{1,3}$ stands for the number of Gaussian peaks used to fit the data, $I_i^{\max}$, Q_i^{opt} and $w_{i,\text{XRD}}$ are the height, center and half-width of the i-th peak, respectively, q_a and q_c represent the obtained coordinate position of the reflection, and $Q_i^{\text{opt}} = q_a^{\text{opt}}$ or q_c^{opt} for the i-th peak with index i omitted for simplicity. Supplementary Figs. 9b,c,e, 11b,c,e and 15b,c,e represent the fitting by Eq. (S1) with $N = 1$. Lattice parameters are calculated from the reflection as

$$\begin{aligned} a &= 2h/\sqrt{3}q_a \\ c &= l/q_c, \end{aligned} \tag{S2}$$

where h and l are Miller indexes of the corresponding reflections. We exploit the c/a ratio as a measure of strain. The value for single crystalline strain-free Cr_2O_3 is 2.739 and is taken as a reference [11]. We notice a gradual reduction of strain with the increase in the thickness of Cr_2O_3 thin film samples.

The RSM of the asymmetric $(1\,0\,\bar{1}\,10)$ reflection of the 30- and 50-nm-thick Cr_2O_3 thin film samples enclose a narrow streak at q_a values of Al_2O_3. This indicates the presence of a pseudomorphic layer of Cr_2O_3 at the interface with Al_2O_3. This layer of Cr_2O_3 is highly stressed, and the crystal lattice parameter a is identical to the one of Al_2O_3. In turn, this leads to a strongly elongated c-axis. In addition, a non-elliptical shape of the reflection suggests the presence of structural inhomogeneity along the thickness of the Cr_2O_3 films, i.e., a strain gradient. This is not the case for a 100-nm-thick Cr_2O_3 film, for which RSM does not reveal signatures of the pseudomorphic layer.

To qualitatively evaluate the strain gradient along the thickness of the Cr_2O_3 thin films, we inspect the RSM in further details. We split the RSM of the $(1\,0\,\bar{1}\,10)$ reflection into sections parallel to q_a and q_c axes (as shown in Supplementary Figs. 10, 12, 16 (XRD Series I) and in Supplementary Figs. 14 and 18 (XRD Series II)) and fit their profiles separately using triple-Gaussian. We analyze the variation of the intensities and positions of individual peaks to assess the lattice parameters of Cr_2O_3 in three structurally different regions along the film thickness: (i) close to the bottom interface, (ii) in the interior of the film and (iii) close to the top surface. The analysis provides a semi-quantitative access to the strain gradient along the thickness of Cr_2O_3 films. The films with thickness of 30 and 50 nm are found to be highly strained at the bottom interface, and the strain gradually relaxes towards the top surface. In contrast to these thinner films, the sample with the thickness of 100 nm reveals only the presence of a small strain gradient at the level of the confidence interval (Supplementary Table 2). For this sample, the $(1\,0\,\bar{1}\,10)$ reflection can be fitted with a single-Gaussian along q_a direction, and a double-Gaussian along q_c direction.

A small difference in the a and c lattice parameters for the 30- and 50-nm-thick samples observed in the region closer to the interface with Al_2O_3 is a result of competition of strain energies, accumulated in Cr_2O_3 thin films. Close to the interface (within the pseudomorphic layer), the a parameter is constrained due to the coherent epitaxial growth and is equal to the a lattice parameter of the Al_2O_3 substrate. At the same time, the c lattice parameter of Cr_2O_3 is not constrained and is dependent on the strain energy that is accumulated in the film. Therefore, we need to compare the a lattice parameter for 50- and 30-nm-thick samples. These two samples reveal the presence of the pseudomorphic

layer. From the analysis of the RSM data (Supplementary Figs. 10, 12), we extract the a lattice parameter at the bottom interface to be 4.75±0.03 Å for 30-nm-thick sample as well as 4.75±0.03 Å for the 50-nm-thick one. The a lattice parameters for these samples are similar to the one of the Al_2O_3 substrate (4.785 Å). In contrast, thicker samples reveal the a lattice parameter of 4.94 Å (100-nm-thick sample) and 4.95 Å (250-nm-thick sample), which is closer to the one of the bulk Cr_2O_3 with $a = 4.96$ Å. The assesed values of the in-plance a and out-of-plance c lattice parameters relying on the RSM and respective c/a ratio is summarized in Supplementary Fig. 26.

The RSM analysis of the thin film samples grown on a Pt(20 nm) seed layer shows a (0 0 0 1) oriented growth of the Cr_2O_3 and does not indicate the presence of any persistent strain of the crystal lattice, independent of the film thickness (Supplementary Fig. 46 for 30-nm-thick and Supplementary Fig. 47 for 250-nm-thick Cr_2O_3 film). Calculated values of the in-plane and out-of-plane lattice parameters of both samples correspond to those of the single crystal.

To confirm the phase purity and epitaxial growth of the magnetron sputtered Cr_2O_3 films, we performed 2θ scans around the (0 0 0 6) reflection (Supplementary Fig. 7) and ϕ-scans of the $(1\,0\,\bar{1}\,10)$ reflection (Supplementary Fig. 8). The 2θ data reveal only reflections from Cr_2O_3 thin film (except for the thinnest one), the Pt cap layer and Al_2O_3 single crystalline substrate, confirming phase purity of the thin films. The ϕ-scans correspond to the rotation around the [0 0 0 1] axis. Hence, by performing a ϕ-scan of 360°, three peaks can be observed which is in accordance with the three-fold symmetry (for rhombohedral syngony) of the [0 0 0 1] axis. By conducting such scans for the Al_2O_3 substrate and grown Cr_2O_3 thin films, the epitaxial relationship between the substrate and thin films can be established. No twinning was observed for our samples.

We note that the Pt cap layer causes pronounced Laue oscillations in most of the scans shown in Supplementary Fig. 7. The coherent scattering thickness of a crystal is $2\pi/\Delta Q$, where ΔQ is the period between the minima of the Laue oscillations in reciprocal space. If we take the first three minima to the left of the Pt (1 1 1) peak, we obtain the corresponding thickness of the Pt capping layer of about 4.1 nm for the 100-nm-thick Cr_2O_3 film, about 3.7 nm for the 50-nm-thick film and about 4.8 nm for the 15-nm-thick film. These values agree with the nominal thickness of the Pt capping layer.

The (0 0 0 6) reflection of the pseudomorphic Cr_2O_3 layer is expected at about 38.3°. This is confirmed by the RSM of the $(1\,0\,\bar{1}\,10)$ reflection where q_c of the pseudomorphic layer is about 7.1 nm^{-1}. It cannot be identified directly due to its much lower intensity ($|F|^2$ of Pt (1 1 1) is almost 15 times larger). Furthermore, the contribution of the pseudomorphic layer is hidden by the Pt (1 1 1) reflection.

For the 50-nm-thick film we observe strong Laue oscillations of a shorter but varying period, which correspond to a thickness of approximately 60 nm. A simulation shows that these oscillations with a varying period and amplitude can result from the combination of a pseudomorphic layer and a partly relaxed Cr_2O_3 layer with a strain gradient. A model with homogeneous layers does not fit these oscillations.

Error bars of the lattice parameters are estimated from instrumental precision of the diffractometer and from the uncertainty of the peak fitting.

Supplementary Note 3. Positron annihilation spectroscopy

Positron measurements were conducted using two complimentary techniques: (i) Doppler broadening variable energy positron annihilation spectroscopy (DB-VEPAS) and (ii) variable energy positron annihilation lifetime spectroscopy (VEPALS). DB-VEPAS depth profiles have been performed at the apparatus for *in situ* defect analysis (AIDA) [12] of the slow positron beamline (SPONSOR) [13]. Positrons have been implanted into samples with discrete kinetic energies E_p in the range between 0.05 and 35 keV, which allowed depth profiling from the film surface down to a couple of micrometers into the substrate. A mean positron implantation depth can be approximated by a material density dependent expression: $\langle z \rangle = 36/\rho \cdot E_p$, where $\rho = 5.22\,\mathrm{g\,cm^{-3}}$ (Supplementary Fig. 20a, top x-axis). After the implantation, positrons lose their kinetic energy due to thermalization. Following a short diffusion, annihilation with electrons take place usually at delocalized lattice sites or localized in vacancy-like defects and at interfaces. The annihilation process leads to the emission of at least two anti-collinear 511 keV gamma photons, which are then detected. At the annihilation site, thermalized positrons have very small momentum compared to electrons. Hence, a broadening of the 511 keV line is observed mostly due to momentum of the electrons. The annihilation photons are registered with one or two high-purity Ge detectors (the former with energy resolution of 1.09 ± 0.01 keV at 511 keV). The broadening is characterized by the so-called S-parameter defined as a fraction of the annihilation spectrum in the middle part (511 ± 0.93 keV). The S-parameter is a fraction of positrons annihilating with low momentum valence electrons and correlates to concentration of vacancy-like defects. The plot of the calculated S-parameter as a function of the positron implantation energy $S(E_p)$ provides the depth dependent information. In the case when the positron diffusion is limited due to a large defect density, the S-parameter will scale directly with the defect concentration and size. In systems where defects are less abundant additional considerations, besides the magnitude of the S-parameter, should be noted before final interpretation, e.g. positron back diffusion to surface, film thickness, or positron affinity [14].

Variable energy positron annihilation lifetime spectroscopy (VEPALS) measurements were conducted on Cr_2O_3 samples at the Mono-energetic Positron Source (MePS) beamline at HZDR [15]. A typical lifetime spectrum $N(t)$ is described by $N(t) = \sum(1/\tau_i)I_i \exp(-t/\tau_i)$, where τ_i and I_i are the positron lifetime and intensity of the i-th component, respectively ($\sum I_i = 1$). All the spectra were deconvoluted using the non-linear, least-squared based package PALSfit fitting software [16] into few discrete lifetime components, which show direct evidence of several different defect types (sizes) (Supplementary Fig. 20). The corresponding relative intensities reflect, to a large extent, the concentration of each defect type (size). In general, the measured positron lifetime is directly proportional to the defects size, i.e., the larger the open volume, the lower the probability and longer it takes for positrons to be annihilated with electrons [14, 17]. The positron lifetime and its intensity has been probed as a function of the positron implantation energy E_p or in the other words implantation depth (thickness).

Theoretical calculations of positron lifetimes for the de- and localized states, i.e., positrons annihilated at interstitial atomic positions (bulk lifetime) and trapped at defect states were obtained using the atomic superposition (ATSUP) method within two-component density functional theory (DFT) ab initio calculations [18]. For the electron-positron correlation, the generalized gradient approximation (GGA) scheme was used [19]. To calculate the electronic structure of defects, we employed a supercell approach placing the defects in the center of supercells. The supercells containing 1920 Cr and O atoms were constructed from the perfect corundum lattice structure (space group 167, with lattice parameters $a = 0.496\,\mathrm{nm}$ and $c = 1.366\,\mathrm{nm}$) with $4 \times 4 \times 4$ hexagonal unit cells. Such supercell sizes are large enough for the accurate positron wave-function determination using the periodic boundary condition, which is natural for supercell calculations. The calculated positron lifetimes corresponding to specific spherical void sizes (vacancy cluster sizes) are plotted in Supplementary Fig. 22.

The low electron momentum fraction S as a function of E_p for Cr_2O_3 films with varied thickness is shown in Supplementary Fig. 20a. The initial steep slope of S for low E_p represents variations of the defect density in the top part (sub-surface region) of the film, which are a consequence of the positron back diffusion to the surface and annihilation at surface states (PAS measurements were conducted on films without Pt cap layer). The variations of S considered from deeper parts of the film reflect changes of open volume more straightforward, since the superposition with surface states is much lower. There S scales directly with the defect density. However, its sensitivity to the defect size is most of the time convoluted with the defect concentration and is difficult to separate. Deeper into the films, S reflects the positron annihilation inside the substrate, where all the curves coincide. The 250 nm sample exhibits the lowest value of the S-parameter among the studied thin films (Supplementary Fig. 20a), which could be taken as an indication of the smallest defect density. Yet, this reduction could be a consequence of a drop in available positron trapping centers in the interface region of the film, i.e., mono-vacancies associated with dislocations, whereas the top part of the film is less affected (see Supplementary Table 1). The average defect size, based on the average positron lifetime τ_{av}, is elevated in the top part of the 250 nm film and at the same time is not much different compared to the other samples with lower thicknesses, see Fig. 20b. Moreover, a larger open volume emerges in the interface part of the film as reflected by the increase in τ_{av} for E_p >4 keV. The 100 nm film shows this additional

maximum related to a larger open volume in the interface region more clearly, for both S and τ_{av} at $E_p \approx 4$ keV. On the other hand, thinner films are characterized with a similar overall open volume, as evidenced from $S(E_p)$, still τ_{av} reveals a small difference close to the interface. Relatively large values of τ_{av} in the top region of all the films, but particularly large contrast between S and τ_{av} for the 250-nm-thick film, are a consequence of a more intense (I_2 in Supplementary Fig. 20j) second positron lifetime component, τ_2 (Fig. 20i), which dominates the overall depth distribution in the sub-surface and interface regions. Considering the initial raise (drop) of I_1 (I_2) in Supplementary Fig. 20j, the sub-surface region itself is not thicker as 20 nm. It gives a threshold for the surface-free signal, once the relative intensity is plateauing or even decreases (as in case of I_1). For thinner films, the sub-surface region is reduced to $\approx$ 10 nm. The second lifetime τ_2 represents vacancy agglomerations (vacancy clusters). The calculated size of the vacancy clusters (assuming spherical geometry) is presented in Supplementary Fig. 22 and Supplementary Table 1. A cluster size of about 8 (4) vacancies is expected for the top (bottom) region of the 250-nm-thick film, respectively. In addition, τ_2 gradually decreases indicating a gradual reduction of the vacancy cluster size along the film thickness, which is evident for the other Cr_2O_3 film thicknesses as well (see Supplementary Fig. 20c,e,g). Considering that the relative intensities are constant in the mid-thickness range, only defect states directly from this area are expected, without surface and substrate contributions. Regarding the first lifetime component τ_1, the interface region has a characteristic defect microstructure compared to the rest of the film. Close to the interface, $\tau_1 = 159 \pm 2$ ps nearly approaches the calculated value for bulk, $\tau_B = 151.7$ ps. Such a scenario is typical for weekly or shallow trapping isolated dislocations [20]. On the other hand, in the top part of the film, τ_1 is larger, although, still below the calculated value for monovacancy, $\tau_{mv} = 197.4$ ps, which is a fingerprint of a "deeper" trapping at dislocations containing vacancies [21]. Depth profiles of thinner films share few common characteristics: (i) τ_1 in the top part of the films indicates dislocations associated with monovacancies in all the cases, whereas (ii) τ_1 from the bottom region is shorter and decreases with the film's thickness, indicating detachment of vacancies from dislocations or alternatively, reduction of both. (iii) The distribution of τ_1 related defects is quite similar, as illustrated by the relative intensities. Finally, (iv) τ_2 shows a gradient starting at the surface, where it is the longest, decreasing down to the interface with the substrate.

To discuss positron lifetimes (τ_1 and τ_2) dependence on the Cr_2O_3 films' thickness in further details, their averaged values from the top and bottom regions are plotted in Supplementary Fig. 21a and 21c with corresponding relative intensities (Supplementary Fig. 21b and 21d). All these data are included in Supplementary Table 1 as well. The analysis of τ_1 indicates a stronger association between dislocations and vacancies in the top part of the film that weakens with increasing the sample thickness. However, the relative intensity I_1 remains similar for all samples independent of the thicknesses, reflecting a deep positron trapping to dislocation-vacancy complexes but potential reduction in their density. It is possible that I_1 compensates due to a large decrease of τ_2 related void size, followed by a lower trapping cross-section. The trapping cross-section scales with size [14]. τ_1 from the interface region changes more abruptly at the 100 nm film thickness, where a transition from dominant dislocation-vacancy complexes ($<$100 nm) to free dislocations ($\geqslant$100 nm) take place. The free dislocations are shallow positron traps, which is reflected in the decrease of I_1. The reduced trapping at dislocations leads to the increase of I_2 and higher annihilation chance with τ_2 related vacancy agglomerations. The void component τ_2 decreases with the film thickness for both the top and bottom regions. It is, however, much longer for the former, which directly evidences a larger void size in the top part of the film compare to the bottom region.

Error bars in Figure 1d,e (main text) and in Supplementary Figs. 20–22 correspond to the standard deviations of the fit.

Supplementary Note 4. Raman spectroscopy studies

We utilize Raman spectroscopy as a complementary tool for the strain analysis in the Cr_2O_3 thin films. Supplementary Fig. 24 shows the Raman spectra for the films with thicknesses of 30 nm, 50 nm, 100 nm and 250 nm. Each column in the figure depicts a spectral range around a certain vibrational Raman mode. The Raman spectra of thin films with the thickness of 30 and 50 nm are dominated by the sharp modes of the sapphire substrate. Most of them are located between 350 and 500 cm^{-1}. The corresponding fitting terms are shown in blue color. For the thicker Cr_2O_3 films (100- and 250-nm-thick samples), the substrate modes become weaker and the Cr_2O_3 phonons start to dominate the Raman spectra.

We resolve three Raman-active phonon modes from the Cr_2O_3 thin films: $E_g(2)$ at 290 cm^{-1}, $A_{1g}(2)$ at 560 cm^{-1} and $E_g(5)$ at 620 cm^{-1}. The other four Raman modes out of the total 7 Raman modes expected for Cr_2O_3 [22] are either too weak or hidden by stronger Raman peaks of the sapphire substrate. According to the lattice dynamics calculations [23], the high frequency $A_{1g}(2)$ and $E_g(5)$ modes are related to oscillations of oxygen ions and the low frequency $E_g(2)$ mode involves mainly the oscillation of the heavier chromium ions. Importantly, all three modes are related to the *in-plane* lattice motion normal to the [0 0 0 1] direction [23]. Therefore, their eigenfrequencies are mainly affected by the change of the lattice parameter a.

As shown in Supplementary Fig. 24, $E_g(2)$ and $E_g(5)$ modes can be fitted by a single Lorentzian peak for all studied films. However, the fitting of the $A_{1g}(2)$ mode is more complex. First, there is a peak from the sapphire substrate at 576 cm^{-1} that overlaps with this mode. Second, in the highly strained 30-nm-thick and 50-nm-thick films, we observe an additional shoulder on the high frequency side that we relate to the inhomogeneous strain distribution along the thickness of the Cr_2O_3 thin film. Therefore, we added a second Lorentzian to obtain a good fit to the experimental data. The obtained frequencies of the Raman modes in Cr_2O_3 are shown in Supplementary Fig. 25. For the $A_{1g}(2)$ mode, we show the frequency of the dominating low-frequency peak. Thus, the estimated strain should be considered as a lower bound suggesting that some part of the thin film can be strained even stronger.

To estimate the in-plane strain $\Delta a/a$, we utilize the coefficients of the frequency shift for each Raman mode, which are known from the high-pressure Raman study of Cr_2O_3 [24]. They are -0.7 $cm^{-1}\,GPa^{-1}$, 3.0 $cm^{-1}\,GPa^{-1}$ and 4.2 $cm^{-1}\,GPa^{-1}$ for the $E_g(2)$, $A_{1g}(2)$ and $E_g(5)$ modes, respectively. Furthermore, the high-pressure XRD data from the same study show that for moderate pressures the strain in Cr_2O_3 can be approximated as $\Delta a/a[\%] = -0.1383 \times P$ [GPa]. Combining this scaling with the known pressure-induced shifts of the Raman phonons, we estimate the strain $\Delta a/a$ based on the position of each Raman mode. The right axes in Supplementary Fig. 25 show the estimated strain. Remarkably, the $E_g(2)$ mode shifts red and the $A_{1g}(2)$ and $E_g(5)$ modes shift blue in the thinner Cr_2O_3 films, but the estimated strain is *compressive* in all cases due to the different signs of the pressure coefficients. This conclusion from the Raman spectroscopy study is in line with the discussion of the XRD-RSM data.

The results from all three Raman modes demonstrate qualitatively the same strain behavior: a negligible strain for the 100- and 250-nm-thick samples and a strong increase of the strain for the 30- and 50-nm-thick samples. However, the absolute values of the strain for the 30-nm-thick film vary from $\approx 0.4\%$ for the $A_{1g}(2)$ mode to almost 2% for the $E_g(2)$ mode. There are two reasons for this deviation. First, in contrast to the modes with the E_g symmetry, the fully symmetric $A_{1g}(2)$ mode involves exclusively the in-phase oscillation of the oxygen ions normal to the [0001] direction [23]. Therefore, it should be sensitive only to the in-plane strain. The E_g modes may be affected by other strain components. Second, the $A_{1g}(2)$ mode produces the strongest peak in the Raman spectrum leading to a high confidence of its fitting. The E_g are much weaker. In addition, the pressure coefficient (sensitivity to the strain) of the $E_g(2)$ is much lower compared to the $A_{1g}(2)$ mode. Based on these arguments, we have chosen the $A_{1g}(2)$ mode strain estimation as the most reliable one. These results are shown in Fig. 1f of the main text demonstrating a good agreement with the XRD-RSM data.

Error bars on the strain values assessed from Raman spectra correspond to the uncertainty of the peak position fitting in Supplementary Fig. 24.

Supplementary Note 5. Magnetotransport investigations

We use magnetotransport measurements to assess the magnetic properties of the top surface of Cr_2O_3 thin films. We monitor the change of the transversal resistance of the Pt electrode prepared on Cr_2O_3 thin films as a function of the out-of-plane applied magnetic field. In this way, the dependence of the transversal resistance on magnetic field $R_{\mathrm{Tr}}(H,T)$ is recorded at different temperatures (Supplementary Fig. 27). The signal is determined by the magnetic state of the underlying Cr_2O_3 film and, thus on the measurement temperature. At a low temperature of 10°C (below the bulk transition temperature of Cr_2O_3), the transversal resistance linearly changes with magnetic field (lines in blue tones). As the temperature increases, a Langevin-type contribution emerges (green-red tones), which indicates the transition of the Cr_2O_3 to the paramagnetic state. We utilize this effect to assess the antiferromagnet-paramagnet transition temperature (Néel temperature) in Cr_2O_3. We fit the measured signal as a sum of the normal Hall effect of the Pt electrode, and the spin Hall magnetoresistance, dependent on the magnetism of the Cr_2O_3 top surface. Namely, we assume that the Pt electrode generates a signal within the linear part of the paramagnetic Langevin-like response. At the same time, the signal caused by the magnetism of the Cr_2O_3 is linear at low temperatures and transforms into the Langevin-like at higher temperatures. To assess the Néel temperature at the top surface of the sample, $T_{\mathrm{N}}^{\mathrm{top}}$, we extract the portion of R_{Tr}, which corresponds to the antiferromagnetic Cr_2O_3.

We focus on the analysis of the magnetotransport data in a high-field region (above 1 T) at temperatures close to the transition temperature. The uniaxial anisotropy of Cr_2O_3 rapidly decreases approaching the phase transition temperature [25], which leads to the respective weakening of the spin-flop field complemented by the introduction of a spin-flop phase at the sample boundary [26]. Having that R_{Tr} is proportional to the net magnetic moment at the top surface of the film [27], the resistance at strong enough fields H reads

$$\begin{aligned} &R_{\mathrm{Tr}}(H,T) = \mu_0[p_1(T) + \chi_{\mathrm{Pt}}(T)]H + p_2(T),\\ &p_1(T < T_{\mathrm{N}}^{\mathrm{top}}) \propto \overline{a} M_0 f(T) + \chi_0 + \chi_0'(T - T_{\mathrm{N}}^{\mathrm{top}}),\\ &p_2(T < T_{\mathrm{N}}^{\mathrm{top}}) = 0,\\ &p_1(T > T_{\mathrm{N}}^{\mathrm{top}}) = 0,\\ &p_2(T > T_{\mathrm{N}}^{\mathrm{top}}) = \mathrm{const.} \end{aligned} \tag{S3}$$

Here, χ_{Pt} measured in Ohm T^{-1} is the coefficient, which characterizes the transversal resistance of the Pt layer as function of temperature and applied magnetic field, M_0 represents the saturation magnetization of one sublattice of Cr_2O_3 at $T = 0$, and the function

$$f(T) = \left(1 - \frac{T^3}{T_{\mathrm{N}}^3}\right)^{\beta} \tag{S4}$$

with $\beta \approx 1/3$, describes the temperature evolution of the sublattice magnetization below the phase transition [28], $\overline{a}$ is a constant of the exchange nature describing the out-of-plane angle of magnetization in the spin-flop phase, $\chi_0 = \chi_{\parallel}(T_{\mathrm{N}}^{\mathrm{top}})$ characterizes the static part of the transversal resistance of Pt for the field applied parallel to c-axis at $T = T_{\mathrm{N}}^{\mathrm{top}}$ and $\chi_0' \equiv \partial\chi_{\parallel}(T < T_{\mathrm{N}}^{\mathrm{top}})/\partial T$ in the vicinity of $T_{\mathrm{N}}^{\mathrm{top}}$ [25]. Above $T_{\mathrm{N}}^{\mathrm{top}}$, only the saturated paramagnetic response remains, which is reflected in the appearance of a constant non-zero p_2, while p_1 becomes zero. These assumptions are valid in a sufficiently narrow range of temperatures around $T_{\mathrm{N}}^{\mathrm{top}}$. We note that the expression (S3) formally describes both, spin-flop and hard c-axis configuration of an antiferromagnet.

The methodology used for the analysis of the magnetotransport data is shown for the 250-nm-thick Cr_2O_3 sample. The linear fits are shown with lines in Supplementary Fig. 28a. Normal Hall effect in Pt (χ_{Pt}) and transverse spin Hall magnetoresistance effect related to the magnetically ordered Cr_2O_3 (p_1) are combined in the slope of linear fit. The coefficient p_2 characterizes the paramagnetic contribution. Supplementary Figs. 28b,c show the temperature dependency of the fitting coefficients $p_1(T)$ and $p_2(T)$ in (S3). In Supplementary Fig. 28c, $p_2(T)$ shows saturating behavior above $T_{\mathrm{N}}^{\mathrm{top}}$, which can be related to the growth of the magnetic susceptibility of Cr_2O_3 above the phase transition temperature in the range of several dozen of degrees [25, 29]. In Supplementary Fig. 28b, it is possible to distinguish three regions with qualitatively different behavior: far below the phase transition (< 30° C), in the vicinity of the phase transition (30°C. . . 40°C), and in the paramagnetic state above the transition temperature (> 40°C). First, the contribution from the normal Hall effect of Pt has to be subtracted. This can be done by the linear fit of $p_1 + \chi_{\mathrm{Pt}}$ as a function of temperature at temperatures above the phase transition (black line in Supplementary Fig. 28b). According to (S3), in this region $p_1 = 0$. This allows us to recover the temperature dependence of $\chi_{\mathrm{Pt}}(T)$. As the normal Hall effect of Pt is linearly dependent on temperature (Supplementary Fig. 44), we subtract this dependence from the data to obtain the signal $R_{\mathrm{Tr},0}(H,T)$ that is proportional to the magnetisation of Cr_2O_3

top surface, see Supplementary Fig. 28d.

$$R_{\mathrm{Tr},0}(H,T) = R_{\mathrm{Tr}}(H,T) - \chi_{\mathrm{Pt}}\mu_0 H. \tag{S5}$$

In Supplementary Fig. 28d, we clearly see the transition from the antiferromagnetic state of Cr_2O_3 (characteristic linear dependence of the transversal resistance on the magnetic field) to the paramagnetic state (characteristic Langevin-like response). The transverse resistance $R_{\mathrm{Tr},0}(H,T)$, evaluated in a strong enough magnetic field ($\mu_0 H > 1\,\mathrm{T}$) above the transition temperature, is constant and levels off at higher temperatures. Thus, the signal that originates from the paramagnetic Cr_2O_3 can be described by p_2 and subtracted to obtain the signal dependent only on the antiferromagnetic order of the Cr_2O_3 top surface

$$R_0(H,T) = R_{\mathrm{Tr},0}(H,T) - p_2(T). \tag{S6}$$

Supplementary Fig. 28e shows $R_0(H,T)$, which is the high field (above 1 T) component of the dependence $R_{\mathrm{Tr},0}(H,T)$ with the subtracted paramagnetic response $p_2(T)$. In this case, the signal grows linearly at low temperatures (blue triangles) and is constant and almost zero at high temperatures (red rhombuses). To evaluate the Néel temperature, we build the offset-free term of the transversal resistance (S3),

$$R_0(T) = R_0(\mu_0 H = 1.25\,\mathrm{T}, T), \tag{S7}$$

see Supplementary Fig. 28f. In the paramagnetic state of Cr_2O_3, the dependence $R_0(T)$ vanishes. Approaching the Néel temperature from the magnetically ordered state, the surface magnetization acquires a component perpendicular to the Cr_2O_3/Pt interface and reveals a typical temperature behavior of the order parameter in Cr_2O_3 [28].

The results of the fitting procedure for the reference Cr_2O_3 single crystal and films of different thickness are shown in Supplementary Fig. 29. A sharp antiferromagnet-paramagnet (AFM-PM) transition at $35\ldots36\,°\mathrm{C}$ is observed for the Cr_2O_3 single crystal, which agrees with the previously obtained data [30]. The transition temperature above 70°C is observed for the 30-nm-thick sample. Error bars on the $T_{\mathrm{N}}^{\mathrm{top}}$ assessed by magnetotransport measurements arises from the smooth decay of the $|R_0(T)|$ to the noise level in the vicinity of AFM-PM transition.

Alternatively, the change of the magnetic state at the Cr_2O_3/Pt interface can be detected by evaluating the change of the slope of the dependence $R_{\mathrm{Tr}}(H = 0, T)$. The transition of the $R_{\mathrm{Tr}}(H = 0, T)$ function from the linear to the Langevin-like shape upon the transition of the Cr_2O_3 to the paramagnetic state is accompanied by a pronounced change of the slope $\partial R_{\mathrm{Tr}}/\partial H|_{H=0}$ at low ($< 100\,\mathrm{mT}$) magnetic fields (lines in Supplementary Fig. 45a). We analyze the variation of this parameter as a function of temperature according to (Supplementary Fig. 45b):

$$f_{\mathrm{s}}(T) = \tanh T, \tag{S8}$$

We extract the transition temperature from the inflection point of the hyperbolic tangent function. The obtained values of the Néel temperature are summarized in Supplementary Fig. 45c. We note that the Néel temperatures estimated accordingly to the two described methods for the analysis of the magnetotransport data are similar within the measurement error.

The Cr_2O_3 film samples in the thickness range of 30. . . 250 nm reveal the criticality behavior near the phase transition temperature similar to the one of the single crystal. This suggests that these thin film samples do not experience finite size effects. However, this is not the case for a 15-nm-thick sample. For this sample, the function $R_0(T)$ close to the Néel temperature reveals the exponent $\beta = 0.667$ instead of $\beta \approx 0.3$ (Supplementary Fig. 42). This is an indication of the onset of finite size effects in this sample [31] and is in agreement with other antiferromagnets, where the finite size effects arise in samples of thickness of the order of 10 nm [32, 33]. Therefore, in our discussion on the flexomagnetic effect, we focus on the data taken of the samples with the thickness of larger than 30 nm, where finite size effects are not pronounced. For further discussions of finite size effects in Cr_2O_3thin films, we refer to [34–36].

The presence of Cr_2O_3 in the stack in addition to the Pt thin film necessarily affects the thermal evolution of the Hall signal measured using the Pt top electrode [1, 37, 38]. The Hall resistance measured on Pt is modified by the underlying Cr_2O_3 due to the finite spin decay length in Cr_2O_3, which allows to alter the Pt conductance by the magnetic ordering of Cr_2O_3.

Furthermore, we note that the samples have different Pt layer thickness. The samples with the Cr_2O_3 layer with the thickness of 15, 30, 50, 100 nm are capped with a 5-nm-thick Pt layer. The 250-nm-thick Cr_2O_3 film and reference single crystal have Pt(3 nm). This explains the difference in the slope for the normal Hall effect in Supplementary Figs. 28 and 44.

The different thickness of Pt layer does not affect the results of our analysis on the Néel temperature. In the Cr_2O_3/Pt system, the measured transversal signal consists of the normal Hall effect of platinum and spin Hall effect of the stack Cr_2O_3/Pt. The contribution from the normal Hall effect in Pt is estimated for each sample individually and is subtracted from the analyzed data.

Supplementary Note 6. Multiscale modelling

Previous theoretical studies have shown the impact of the epitaxial strain on ferro- and antiferromagnetic order parameters in Cr_2O_3 [39]. Here, we employed a multiscale approach to evaluate the effect of an asymmetric load on magnetic properties at a microscopic level (Supplementary Fig. 39). Note that the symmetry of the load prevents the periodic boundary conditions. Furthermore, the experimentally relevant length scale along the sample thickness is several 10s of nm. Therefore, we carry out the structural determination relying on classical potentials such as Buckingham or ReaxFF [40, 41]. However, the proper theoretical framework for the local anisotropy calculations would require the complete active space SCF method (CASSCF) treatment [42].

We have developed a fully atomistic model to assess the strain propagation through the interior of the thin films. The original corundum block of 40Å×40Å×60Å in size (Supplementary Fig. 39a) was strained by $\sim 4\%$ at the bottom interface. The decrease of the compression in the xy-plane across the film thickness is assumed to be linear, and the slope is adjusted to achieve strain free conditions at the top interface. As there is no translational symmetry, this supercluster was simulated in the $60\times60\times100\,\text{Å}^3$ boxes without periodic boundary conditions using ReaxFF potentials and model as an implement in the Lammps code [43]. Also, the outer atomic layers (except the top surface) were fixed throughout the simulation, forming a border shell. These border constraints prevent a significant surface reconstruction during the molecular dynamics run. At the same time, the free condition on the top surface makes it possible for the system to buckle along z-axis to relieve part of the excess tension. The cross-section of the optimized structure (equilibrated at 10 K) is shown in Supplementary Fig. 39b.

Early DFT studies have shown that the local anisotropy potentially can change the sign (*easy*-axis to *easy*-pane transition). At the same time, the effective couplings (exchange) and the Dzyaloshinskii-Moriya interaction (DMI) change linearly with the epitaxial strain, so at 4% strain, the system gains 25% in the coupling strength [39]. Therefore, the unloaded (or symmetrically loaded) Cr_2O_3 has alternating moments along the c-axis to keep the AFM $\{+-+-\}$ order, and so it is fully compensated for a defect-free block. However, for the asymmetrically strained case (Supplementary Fig. 39), the $[CrO_6]$ polyhedrals are differently deformed along the $z(c)$-axis, which affects the local anisotropy, as well as the orientation for the relative magnetic frame and the moment projections (Supplementary Figs. 39b, 39f). These changes can be followed at CASSCF/ANO-RCC-MB/RASSI-SO level using the OpenMolcas code [42]. The middle yz-cut region contains roughly 1000 $[CrO_6]$ polyhedrals from top to bottom, as shown in Supplementary Fig. 39e (one layer is shown for clarity). For every single polyhedral in this slab, the ^4F multiple structures were evaluated including anisotropy, magnetic frame orientation, and g-tensor for the two lowest Kramers SOC-doublets (pseudospin $\tilde{S} = 1/2$ model). The point charge approximation around the Cr^{3+} ion was used. The charges on the oxygen positions were optimized such, that for the bottom layer of Cr atoms have the second-order zero-field splitting parameter (D) close to the experimental D-value for the ruby ($Cr^{3+}@Al_2O_3$). This experimental value is $-6.0\,\text{GHz}$ ($= -0.2\,\text{cm}^{-1} = -0.025\,\text{meV}$) [44, 45], the theoretical value $D_{\text{th}} = -0.3\,\text{cm}^{-1}$.

Supplementary Figs. 39c, 39d combines scatter plots in yz-plane colorized according to l_z (on-site momentum) and D values for different Cr^{3+} sites and as function of z coordinate. From the bottom to the top, the g_z values form a banded front (levels) with the biggest $l_z = 2.8\mu_\text{B}$ with the observed minimum of $\sim 2.0\mu_\text{B}$ in the top layers. The second-order zero-field splitting parameter D in the bottom layer has a mean value around $-0.3\,\text{cm}^{-1}$ and moves towards $-0.1\ \text{cm}^{-1}$ as the strain lessens with z. Above $z \sim 40\,\text{Å}$ linearity is wavering with D approaching $> -0.05\,\text{cm}^{-1}$ mark. These changes are even more pronounced in the topmost (surface) layer; such behavior is a subject for further studies. Undoubtedly due to the same effects, we find that with z the deviation of g_z within each layer grows, as shown in Supplementary Fig. 39d. Nevertheless, one can derive the mean slope of 0.03 $(\pm4\%)\,\mu_\text{B}\,\text{nm}^{-1}$. Moreover, taking into account definitions (S23), (S25) and the fact that the flexomagnetism-induced magnetization M_fm is roughly $\sim \Delta l_z t/\Delta V$ while enforced strain of 4%, we estimate the flexomagnetic coefficient as

$$\mu_0^g = \frac{\Delta l_z}{S(\Delta u_{xx} + \Delta u_{zz})} = (12.0 \pm 0.5)\,\mu_\text{B}\,\text{nm}^{-2}, \tag{S9}$$

where S is unstrained Cr_2O_3 unit cell surface area in the ab-plane, ΔV its volume and $t \sim 4\,\text{nm}$ is the region where the slope is strictly linear.

Further insight can be governed by following a relative rotation of the magnetic frame for each CrO_6 polyhedral (Supplementary Figs. 39c, 39e). The buckling of the structure apparently moves the local anisotropy towards an easy-plane solution (the angle between M_z and M_z' close to $90°\pm10°$). The value (S9) is comparable with the flexomagnetic coefficient of Mn_3GaN $\mu_0^{g'} \sim 4\,\mu_\text{B}\,\text{nm}^{-2}$ theoretically predicted by Lukashev and Sabirianov [46]. According to the temperature-dependent electron spin resonance (ESR) data for the ruby system, the local anisotropy and g-tensor stays unchanged up to 575 K [47]. It comes as the next multiple E_2 lies in $\sim 10000\,\text{cm}^{-1}$ and thermal expansion in this range is small [47]. Although it would not be fully accurate to extrapolate this stability on all possible deformed cases of $[CrO_6]$ (as exact $D(T)$ and the thermal expansion coefficients are unknown), it would be fair to assume that

the μ_0^g of $12\,\mu_{\rm B}\,{\rm nm}^{-2}$ ($\pm 4\%$) will be valid for temperatures of the order of $O(10^2)$ K and covers experimentally relevant range.

Supplementary Note 7. Model of an inhomogeneously strained thin Cr_2O_3 film

A. Piezo- and flexomagnetic effects

Depending on the symmetry of the atomic lattice, the mechanical strain and its gradients can influence magnetic and thermodynamic properties of the crystal. Within the conventional phenomenological approach, it is convenient to expand the density of the thermodynamic potential $\Phi = \int \phi \mathrm{d}\mathbf{r}$ into series based on the strain tensor

$$u_{ij} = \frac{1}{2}\left(\frac{\partial u_i}{\partial x_j} + \frac{\partial u_j}{\partial x_i}\right) \tag{S10}$$

with u_i, $i = x, y, z$ being components of the displacement field, magnetization $\mathbf{M}$, their derivatives $\partial u_{ij}/\partial x_k$ and $\partial M_i/\partial x_j$, and magnetic field $\mathbf{H}$. The main term in the series stands for the piezomagnetic effect. This term is linear in field and strain and reads [48]

$$\phi_{\text{piezo}} = -\widetilde{\lambda}_{ijk} H_i u_{jk}, \quad i, j, k = x, y, z. \tag{S11}$$

The piezomagnetic effect constitutes the appearance of a finite magnetization when the sample is exposed to a mechanical strain,

$$M_i^{\text{piezo}} = \widetilde{\lambda}_{ijk} u_{jk}. \tag{S12}$$

with $\widetilde{\lambda}_{ijk}$ being the piezomagnetic coupling tensor. The relation (S11) is often introduced via the stress tensor $\sigma_{ij} = C_{ijkl} u_{kl}$, $i, j, k, l = x, y, z$, where C_{ijkl} is the elasticity tensor. Physical quantities such as the magnetization of the piezomagnetic origin and the Néel temperature at the coordinate $\mathbf{r}$ are determined by the value of the strain tensor $u_{ij}(\mathbf{r})$.

The spatial inhomogeneity of u_{ij} is taken into account by the subsequent terms in the expansion of the thermodynamic potential series with the spatial derivatives of the magnetization and strain tensors [49],

$$\phi_{\text{flex}} = \widetilde{\mu}_{ijkl}\left(M_k \frac{\partial u_{ij}}{\partial l} - u_{ij}\frac{\partial M_k}{\partial l}\right), \quad i, j, k, l = x, y, z \tag{S13}$$

with $\widetilde{\mu}_{ijkl}$ being the flexomagnetic coupling tensor. The flexomagnetic effect is the appearance of a finite magnetization

$$M_k^{\text{flex}} = -\widetilde{\mu}_{ijkl} \partial u_{ij}/\partial l \tag{S14}$$

due to the spatial inhomogeneity of the strain. The constant shift of u_{ij} with respect to the unstrained sample does not influence M_k^{flex}. We note that other terms in the thermodynamic potential, e.g., bilinear in strain or magnetization, are not mentioned here, being irrelevant for this discussion. Still, we note that their presence is expected to change the expressions for M_k^{flex} and M_k^{piezo}.

To illustrate the difference between piezo- and flexoeffects, let us consider a model example of the uniaxial strain $u = u_0 + wz$ with u_0 characterizing a hydrostatic pressure and the coefficient w determining the strain gradient, which impacts magnetism of the sample. To illustrate the difference between piezo- and flexoeffects, let us consider a model example of the uniaxial strain $u = u_0 + wz$ with u_0 characterizing a hydrostatic pressure and the coefficient w determining the strain gradient, which impacts magnetism of the sample. Then, the total magnetic moment induced by the piezomagnetism is proportional to the mean value of strain:

$$\mathrm{M}^{\text{piezo}} \propto \int (u_0 + wz)\mathrm{d}\mathbf{r} \propto \langle u \rangle. \tag{S15}$$

The magnetic moment of the flexomagnetic origin is determined by w only:

$$\mathrm{M}^{\text{flex}} \propto \int w \mathrm{d}\mathbf{r} = wV \tag{S16}$$

with V being the sample volume. In this way, while the piezoeffect related to the strain itself is determined by the average strain, the flexoeffect is determined by the strain gradient. We stress that piezoeffects are determined by the value of strain u itself, which may vary from point to point, and flexoeffects are determined by the spatial derivatives of u. Furthermore, we emphasize on the difference between the thickness-dependent effects and the effects, where a given physical quantity is dependent on the coordinate z along the thickness of the sample. Mathematically, the thickness-dependent effects are described as the dependence of a physical quantity y on the thickness t, i.e. $y = f(t)$. For example, the piezomagnetic effect has a thickness dependence. This means that the strength of the piezomagnetism in samples of different thickness is different. In contrast, the effects, where a physical quantity y is dependent on the coordinate z along the thickness are described as $y = f(z; t = t_0)$ for the specific thickness of the sample t_0. This can be taken as a description of flexoeffects, which are in the focus of this work.

B. Distribution of the Néel temperature

We consider a thin film of thickness t under compressive strain, which is uniform in xy-plane. Structural characterization (Fig. 1 of the main text; see also Supplementary Notes 1–4) shows that there is a gradient of c/a along the thickness of thin films. We assume that the lattice parameters read

$$\begin{aligned} a(z) &= a_0 - \Delta a(z), \qquad c(z) = c_0 + \Delta c(z),\\ \Delta a(z) &= a_1 - a_2 z, \qquad \Delta c(z) = c_1 - c_2 z, \end{aligned} \tag{S17}$$

where $a_0 = 4.96\,\text{Å}$ and $c_0 = 13.59\,\text{Å}$ [11] are the lattice parameters of bulk Cr_2O_3, the coefficients $a_{1,2}, c_{1,2} > 0$ and the $\hat{z}$ axis is directed perpendicularly to the film. Here, pairs (a_1, c_1) and (a_2, c_2) are responsible for the homogeneous (piezomagnetic effect) and inhomogeneous (flexomagnetic effect) strain, respectively. The Néel temperature is a linear function of the c/a parameter [50, 51]. Thus, we can write [52]

$$T_{\text{N}} \approx T_0 + \frac{\partial T}{\partial\left(\Delta\frac{c}{a}\right)}\left(\Delta\frac{c}{a}\right) = T_0 + \Delta T_{\text{piezo}} + \Delta T_\zeta(z) = T_0 + T_{\text{N}}^{\text{top}} + \zeta\times(t-z), \tag{S18}$$

where $T_0 = 307\,\text{K}$ (35°C) is the Néel temperature of bulk Cr_2O_3 [53]. Our thin films with thicknesses of 30 and 50 nm possess an inhomogeneous strain. According to the definition (S15), we associate ΔT_{piezo} in Eq. (S18) with the *average* strain in the film. The last term in the expression for T_{N} [Eq. (S18)] describes the change of the Néel temperature due to the inhomogeneous strain distribution along the film thickness. We define (S18) in such a way that the coefficient ζ is non-negative and measured in units of $^\circ\text{C}\,\text{nm}^{-1}$. In the following, we will use notations $T_{\text{N}}^{\text{top}} = T_{\text{N}}(t)$ and $T_{\text{N}}^{\text{bot}} = T_{\text{N}}(0)$ for values of the Néel temperature at the top and bottom interfaces of the thin film, respectively. An estimation of the piezomagnetic enhancement of the Néel temperature can be done using the results of the DFT and Monte Carlo calculations, provided in Kota *et al.* [50]:

$$\Delta T_{\text{piezo}}\left(\left\langle\frac{c}{a}\right\rangle\right) \approx 251.7\left[\left\langle\frac{c}{a}\right\rangle - \frac{c_0}{a_0}\right]\,^\circ\text{C}. \tag{S19}$$

Eq. (S19) allows us to estimate the piezomagnetic contribution to the Néel temperature based on the *average* c/a ratio, which is known with a good accuracy from the experiment and provides an estimate of strain in the film (Fig. 1h in the main text). The calculations corresponding to the Eq. (S19) are shown in Supplementary Fig. 41 with purple symbols. We note that these predictions are comparable with the measured enhancement of the transition temperature in magnetotransport experiment (blue symbols) and estimated average Néel temperature (green symbols). The average Néel temperature is calculated as $(T_{\text{N}}^{\text{top}} + T_{\text{N}}^{\text{bot}})/2$ (see Supplementary Note 7 F). The Néel temperature at the top surface decreases with thickness due to decrease in strain. This is in agreement with the misfit-induced-strain models predicting $\langle u_{ij}\rangle \propto 1/t$ [54–57].

The thickness of the film, which remains in the antiferromagnetic state at the specific temperature reads

$$t_{\text{AFM}} = \begin{cases} t, & T < T_{\text{N}}^{\text{top}},\\ \dfrac{T_{\text{N}}^{\text{top}} + \zeta t - T}{\zeta}, & T_{\text{N}}^{\text{top}} < T < T_{\text{N}}^{\text{bot}},\\ 0, & T > T_{\text{N}}^{\text{bot}}. \end{cases} \tag{S20}$$

C. Thermodynamic potential

The macroscopic properties of Cr_2O_3 can be described by the primary antiferromagnetic order parameter, i.e., the Néel vector $\mathbf{L} = \overline{\mathbf{M}}_1 - \overline{\mathbf{M}}_2 + \overline{\mathbf{M}}_3 - \overline{\mathbf{M}}_4$ with $\overline{\mathbf{M}}_i$, $i = \overline{1,4}$ being the sublattice magnetizations and the total magnetization being given by $\mathbf{M} = \overline{\mathbf{M}}_1 + \overline{\mathbf{M}}_2 + \overline{\mathbf{M}}_3 + \overline{\mathbf{M}}_4$. Each of the sublattice magnetizations follows the temperature dependence (S4) [28].

The bulk Cr_2O_3 possesses $\mathbf{M} = 0$ and the stray fields detectable by the nitrogen vacancy (NV) magnetometry are associated with the uncompensated magnetic moments at the sample's interface (0 0 0 1) [2, 30, 58]. In addition, the crystal structure of Cr_2O_3 allows for the Dzyaloshinskii-like response [59–61] (appearance of the magnetization in the presence of an inhomogeneous antiferromagnetic order parameter; the term of similar structure is also called as the flexoantiferromagnetic term in [61]) and the flexomagnetic response (appearance of the magnetization in the presence of an inhomogeneous strain) [61, 62]. We consider a magnetic state of the Cr_2O_3 film close to the transition temperature to the paramagnetic state in the absence of strong magnetic fields and neglect the relativistic effects on

$\mathbf{L}$ in the thermodynamic potential, which does not change the following conclusions qualitatively. Thus, we can write the following density of the thermodynamic potential for the ferro- and antiferromagnetic order parameters $\mathbf{M}$ and $\mathbf{L}$, respectively:

$$\phi = \phi_0 - AL^2 + CL^4 + DM^2 + \widetilde{\nu}_{kz}\left(M_k \frac{\partial L}{\partial z} - L\frac{\partial M_k}{\partial z}\right) + \widetilde{\mu}_{ijkz}\left(M_k \frac{\partial u_{ij}}{\partial z} - u_{ij}\frac{\partial M_k}{\partial z}\right), \quad i,j,k = x,y,z. \tag{S21}$$

Here, ϕ_0 contains non-magnetic and weaker magnetic contributions, $A(T) > 0$ and $C > 0$ determine the length L of the antiferromagnetic order parameter $\mathbf{L}$ within the Landau theory of phase transitions ($A > 0$ if $T < T_{\text{N}}$ and $A \equiv 0$ otherwise), $D > 0$ preserves zero magnetization ($|\mathbf{M}| = M = 0$) in the ground state of a bulk material. The term with the tensor $\widetilde{\nu}_{ij}$, $j = z$ determines the Dzyaloshinskii-like response of the sample, which links changes of $\mathbf{L}$ and $\mathbf{M}$. In a general case of an anisotropic antiferromagnet, this is the third-rank tensor. However, in the isotropic case, one of the indices can be omitted. The last term, with the flexocoupling tensor $\widetilde{\mu}_{ijkl}$, $l = z$, determines the flexomagnetic response induced by the gradient of the strain tensor u_{ij}. Assuming $M \ll L$, the equilibrium values of the order parameters in the antiferromagnetic phase read

$$L \approx \sqrt{\frac{A}{2C}} = 4M_0 f(T), \tag{S22a}$$

$$M_k = \underbrace{-\nu_{kz}\frac{\partial L}{\partial z}}_{M_{\text{d-l}}} \underbrace{-\mu_{ijkz}\frac{\partial u_{ij}}{\partial z}}_{M_{\text{fm}}}, \quad i,j,k = x,y,z, \tag{S22b}$$

where M_0 is the sublattice magnetization at $T = 0\,\text{K}$, $\nu_{kz} \equiv \frac{\widetilde{\nu}_{kz}}{D}$ and $\mu_{ijkz} \equiv \frac{\widetilde{\mu}_{ijkz}}{D}$ are tensors of the Dzyaloshinskii-like response and flexomagnetism, respectively, c.f. Eq. (S14). The magnetization (S22b) is absent in the paramagnetic state [62]. We note that formally, the mechanisms of the appearance of a finite $\mathbf{M}$ discussed in [60] and [61] can be accommodated in the tensor ν_{ij}, $i,j = x,y,z$. However, they have different symmetries. In particular, the flexoantiferromagnetic mechanism leads to $M_z \neq 0$ only via finite in-plane gradients of $\mathbf{L}$, while the bulk magnetization of the exchange nature predicted in [59, 60] is described by the first-rank tensor. All mechanisms leading to the magnetization of Cr_2O_3 films are summarized in schematics shown in Supplementary Figs. 37, 40, 49, 50, 51 (see also Methods section of the main text).

The approach (S21) states the presence of specific energy terms via the symmetry considerations of the given crystal only and does not derive such coefficients like ν_{ij} or μ_{ijkl} quantitatively. The latter should be done considering microscopic models of the given medium, e.g. as it is discussed in Supplementary Note 6.

D. Flexomagnetically induced magnetization

The average absolute value of the flexomagnetic coefficient determining the induced ferromagnetic moment by the strain gradient reads

$$\mu = \langle \mu_{ijkz} \rangle = \frac{M_{\text{fm}}}{\langle \partial u_{ij}/\partial z \rangle}. \tag{S23}$$

The average strain gradient $\langle \partial u_{ij}/\partial z \rangle$ can be estimated as follows. According to (S17), the ratio between c and a lattice parameters reads

$$\frac{c}{a} \approx \frac{c_0}{a_0}\left(1 + |u_{xx}| + u_{zz}\right), \quad u_{xx} = u_{yy} = -\frac{\Delta a}{a_0}, \quad u_{zz} = \frac{\Delta c}{c_0}. \tag{S24}$$

with the minus sign originating from the definition of Δa in (S17). Then, the strain gradient reads

$$\left\langle \frac{\partial u_{ij}}{\partial z} \right\rangle = \frac{1}{t}\frac{\left|(\frac{c}{a})|_{\text{top}} - (\frac{c}{a})|_{\text{bot}}\right|}{(\frac{c_0}{a_0})} = \frac{\left|(|u_{xx}| + u_{zz})|_{\text{top}} - (|u_{xx}| + u_{zz})|_{\text{bot}}\right|}{t} = \frac{\Delta u_{xx} + \Delta u_{zz}}{t}, \tag{S25}$$

where $\Delta u_{xx} = \left|u_{xx}|_{\text{top}} - u_{xx}|_{\text{bot}}\right|$ and $\Delta u_{zz} = \left|u_{zz}|_{\text{top}} - u_{zz}|_{\text{bot}}\right|$. Using the estimation (S9) for μ, this allows us to expect that the ferromagnetic moment of the flexomagnetic origin in the 50-nm-thick film is of the order $0.7\,\mu_{\text{B}}\,\text{nm}^{-2}$,

see Supplementary Table 4. We note that there is no independent strain along different axes. With the compressive strain originating from the Al_2O_3 substrate, the Cr_2O_3 lattice adopts the c lattice parameter if all degrees of freedom of the lattice are taken into account [63]. Thus, having the in-plane strain components $u_{\rm ip} := u_{xx} = u_{yy}$, which are determined by the Al_2O_3 substrate, the out-of-plane strain component $u_{zz} = u_{zz}(u_{\rm ip})$ and the strain tensor u_{ij} is a function of a single variable, which can be chosen as the c/a ratio.

The flexomagnetic tensor for $\bar{3}'m'$ crystals including Cr_2O_3 has 10 non-zero independent components out of 81 ones (37 non-zero components in total) [62]. This allows to predict the total flexomagnetic response within the phenomenological theory based on symmetry considerations. Having the strain gradient along c-axis (z axis is aligned with c-axis, x and y axes are in c plane), the induced magnetic moment reads

$$\begin{aligned} M_{\mathrm{fm}\,x} &= -\mu_{xxxz}\left(\frac{\partial u_{xx}}{\partial z} - \frac{\partial u_{yy}}{\partial z}\right) - 2\mu_{xzxz}\frac{\partial u_{xz}}{\partial z}, \\ M_{\mathrm{fm}\,y} &= 2\mu_{xxxz}\frac{\partial u_{xy}}{\partial z} - 2\mu_{xzxz}\frac{\partial u_{yz}}{\partial z}, \\ M_{\mathrm{fm}\,z} &= -\mu_{xxzz}\left(\frac{\partial u_{xx}}{\partial z} + \frac{\partial u_{yy}}{\partial z}\right) - \mu_{zzzz}\frac{\partial u_{zz}}{\partial z}, \end{aligned} \tag{S26}$$

see Supplementary Fig. 50. In absence of shear ($u_{xz} \equiv u_{xy} \equiv u_{xz} \equiv 0$) and uniform in-plane strain, the magnetization is proportional to the coefficients μ_{xxzz} and μ_{zzzz}, and aligned with the c-axis. In the following, we label the contribution to the flexomagnetism from the distortion of the g-tensor only (Supplementary Note 6) via superscript g. Note, that in Eliseev *et al.* [62], the last two indices of the flexomagnetic tensor have the opposite order, i.e., coordinate–(magnetization component) instead of (magnetization component)–coordinate used in expression (S21).

It is instructive to consider the case of the strain gradient applied along the x axis (e.g., for the Cr_2O_3 epitaxially grown in such a way to have c-axis parallel to the substrate and the x being out-of-plane perpendicular to the interface). Then the flexomagnetic response reads

$$\begin{aligned} M_{\mathrm{fm}\,x} &= -\mu_{xxxx}\frac{\partial u_{xx}}{\partial x} - \mu_{xxyy}\frac{\partial u_{yy}}{\partial x} - \mu_{zzxx}\frac{\partial u_{zz}}{\partial x} - 2\mu_{xzxx}\frac{\partial u_{xz}}{\partial x}, \\ M_{\mathrm{fm}\,y} &= (\mu_{xzxx} + \mu_{xzzx})\frac{\partial u_{yz}}{\partial x} - 2\mu_{xyyx}\frac{\partial u_{xy}}{\partial x}, \\ M_{\mathrm{fm}\,z} &= -2\mu_{xzzx}\frac{\partial u_{xz}}{\partial x} - \mu_{xxzx}\left(\frac{\partial u_{xx}}{\partial x} - \frac{\partial u_{yy}}{\partial x}\right). \end{aligned} \tag{S27}$$

Unlike the case (S26), even in absence of shear, there is the magnetization component aligned with the gradient ($M_{\mathrm{fm}\,x}$) and along the c-axis ($M_{\mathrm{fm}\,z}$). They are determined by μ_{xxxx}, μ_{xxyy}, μ_{zzxx}, and by μ_{xxzx}, respectively. Thus, the magnitude and direction of the flexomagnetic response is strongly dependent on the way how the strain inhomogeneity is applied to the sample.

E. Thermally induced magnetization and flexomagnetic effect

The spatial distribution of the Néel temperature along the film thickness (S18) leads to the scaling of the sublattice magnetization along the z coordinate even for the homogeneously heated sample at a temperature T. To describe this effect, we consider a unit cell of Cr_2O_3 of volume V and four classical magnetic moments $\mathbf{m}_i$, $i = \overline{1,4}$ arranged along the c-axis. The i-th magnetization is linked with the respective magnetic moment as $\overline{\mathbf{M}}_i = \mathbf{m}_i/V$. The length of these magnetic moments is determined by the sample's temperature and position in the lattice,

$$\begin{aligned} \mathbf{m}_1 &= \mathbf{m}_0\left(T, z + 3\Delta z/2\right), \\ \mathbf{m}_2 &= -\mathbf{m}_0\left(T, z + \Delta z/2\right), \\ \mathbf{m}_3 &= \mathbf{m}_0\left(T, z - \Delta z/2\right), \\ \mathbf{m}_4 &= -\mathbf{m}_0\left(T, z - 3\Delta z/2\right), \end{aligned} \tag{S28}$$

where Δz is the vertical distance between the nearest Cr ions, which is assumed to be the same for all of them for simplicity, see Supplementary Fig. 37. Here, $\mathbf{m}_0(T,z) \equiv \mathbf{m}_0[T/T_{\rm N}(z)] \equiv \mathbf{m}_0(z)$, which renders the different length of $\mathbf{m}_i$, $i = \overline{1,4}$. Expanding (S28) into Taylor series up to the second order by Δz, we find the uncompensated magnetic moment per unit cell together with the magnetization:

$$\Delta\mathbf{m} \approx 2\frac{\partial \mathbf{m}_0(z)}{\partial z}\Delta z, \quad \mathbf{M}(z) = \frac{\Delta\mathbf{m}}{V} \approx \frac{2}{S}\frac{\partial \mathbf{m}_0(z)}{\partial z}, \tag{S29}$$

where $S = V/\Delta z$. Taking into account, that $\mathbf{L} \approx 4\mathbf{m}_0/V$, the magnetization (S29) reads

$$\mathbf{M}(z) = \frac{\Delta z}{2}\frac{\partial \mathbf{L}}{\partial z}. \quad \text{(S30)}$$

Taking into account the explicit dependency of the Néel vector on temperature (S22a) and the functional dependency of the Néel temperature on strain, $T_{\rm N} \equiv T_{\rm N}(u_{ij}(z))$, the thermally induced magnetization reads

$$\mathbf{M}(z) = \frac{\mathbf{L}}{L} \underbrace{\frac{2M_0\Delta z}{[1 - T^3/T_{\rm N}(z)^3]^{2/3}}\frac{T^3}{T_{\rm N}^4}\frac{\partial T_{\rm N}}{\partial u_{ij}}}_{\substack{\mu^{\zeta}_{ij(x,y,z)z}\text{, contribution to the}\\ \text{tensor of flexomagnetism}}} \times \frac{\partial u_{ij}}{\partial z}. \quad \text{(S31)}$$

We note that this expression follows the definition of the flexomagnetic effect (S14) and, therefore, contributes to the flexomagnetic effect. Since the rotation of the unit vector $\mathbf{L}/L$ inside the domain walls should follow the in-plane crystallographic axes, the symmetry of $\mathbf{M}(z)$ (S31) is the same as $\mathbf{M}_{\rm fm}$ (S26).

Following Supplementary Note 6 and Eq. (S31), the flexomagnetic effect has two contributions, μ^g_{ijkl} and μ^ζ_{ijkl}

$$M_{{\rm fm},i} = (\mu^g_{jjiz} + \mu^\zeta_{jjiz})\frac{\partial u_{jj}}{\partial z}, \quad i,j = x,z, \quad M_{{\rm fm},x,y} = 0. \quad \text{(S32)}$$

The inhomogeneous mechanical deformation of the crystal lattice transforms the CrO_6 polyhedra and thus modifies the g-tensor of Cr-ions of the lattice grid, which gives $\langle \mu^g_{ijkl}\rangle \sim \mu^g_0$ (see Eq. (S9)). This contribution does not follow the direction of $\mathbf{L}$ and cannot be detected by the NV magnetometry. The thermal reduction of the sublattice magnetization is inhomogeneous due to the spatial distribution of the strain tensor $u_{ij}(z)$ and the respective distribution of $T_{\rm N}(z)$. To estimate $\langle \mu^\zeta_{ijkl}\rangle$, we use $\zeta = 0.76°{\rm C\,nm}^{-1}$ determined for the sample with the film thickness of 50 nm (Supplementary Table 3), the magnetic moment of the sublattice $2.8\,\mu_{\rm B}$ [64], volume of the unit cell $V = 0.29\,{\rm nm}^3$ [11] and $\Delta z = c_0/4$. The derivative $\langle \partial T_{\rm N}/\partial u_{ij}\rangle \sim 251.7c_0/a_0$ (see Eq. (S19)). Then, at $T = 20°$ C, the average of the flexomagnetic coefficient, which comes from the thermal reduction of the sublattice magnetization, along the film thickness is $\langle \mu^\zeta_{ijkl}\rangle \sim 15\,\mu_{\rm B}\,{\rm nm}^{-2}$, see Supplementary Fig. 38. We note that the large value of $\langle \mu^\zeta_{ijkl}\rangle$ is determined by the closeness to the temperature of the antiferromagnet-paramagnet transition and $\langle \mu^\zeta_{ijkl}\rangle \ll \mu^g_0$ at low temperatures.

To get the total magnetic moment per unit area of the film, we integrate (S30) using the explicit functional dependency of $\mathbf{m}_0$ on the profile of the Néel temperature (S18) and obtain

$$M_\zeta(T) = 2M_0 \int_0^{t_{\rm AFM}} \frac{1}{[1 - T^3/T_{\rm N}(z)^3]^{2/3}}\frac{T^3}{T_{\rm N}^4}\frac{\partial T_{\rm N}}{\partial z}{\rm d}z. \quad \text{(S33)}$$

Being determined by $\mathbf{L}$, $\mathbf{M}_\zeta$ changes the direction within the oppositely oriented antiferromagnetic domains together with the surface uncompensated moments and cannot be directly extracted from the analysis of the NV measurements as carried out in this work.

Far from $T^{\rm top}_{\rm N}$, sublattices are almost compensated, see Supplementary Fig. 52 and brown curve in Fig. 4d of the main text. In the vicinity of $T^{\rm top}_{\rm N}$, the surface magnetization at the top surface almost vanishes. At the same time, the bottom interface is still far from the transition temperature. This difference in the magnetic state at the top and bottom interfaces leads to a rapid increase in the total magnetic moment of the film close to $T^{\rm top}_{\rm N}$, see Supplementary Fig. 52 and red curve in Fig. 4d of the main text. For temperatures $T^{\rm top}_{\rm N} < T < T^{\rm bot}_{\rm N}$ with $T^{\rm bot}_{\rm N}$ being the Néel temperature at the bottom interface, M_ζ is reduced with temperature because of the reduction of the thickness of the antiferromagnetically ordered part of the film, see Fig. 4a–c of the main text.

In the following, we consider the spatial variation of M_ζ. The expression (S30) also follows the symmetry of the mechanism discussed for Cr_2O_3 in Andreev [60] (Supplementary Fig. 51):

$$\mathbf{M}_{\rm A} = \nu_i \frac{\partial(\mathbf{L}/L)}{\partial x_i}, \quad i = x,y,z \quad \text{(S34)}$$

with ν being a vector of the exchange nature. The difference is that the expression (S34) considers only the spatial variation of the direction of $\mathbf{L}$ (vector $\mathbf{L}/L$ is the unit one), while in our case (S30), the finite magnetization originates from the variation of the length of $\mathbf{L}$. Thus, being similar by symmetry, these mechanisms (S30) and (S34) have different physical origin.

We emphasize that the discussed mechanism is different by the symmetry also from the flexoantiferromagnetic effect discussed by Kabychenkov and Lisovskii [61] (Supplementary Fig. 51). The magnetic moment (S33) follows the spatial texture of $\mathbf{L}$, while flexoantiferromagnetic magnetization, if present, is mainly determined by the in-plane components of $\mathbf{L}$ [61]. Also, M_ζ contributes to the NV signal in the same way as the uncompensated spins at the top and bottom film interfaces. We note that the magnetization originating at the lattice defects [1] follows the direction of $\mathbf{L}$ similar to the thermally induced one.

Both terms of Andreev [60] and Kabychenkov and Lisovskii [61] are expected to be small. Indeed, by symmetry, the respective magnetic moment is similar to the moment, which is generated by the non-collinear textures in one-dimensional antiferromagnetic spin chains [65, 66] where it is of the order of $a/\ell \times (7/2)\mu_{\text{B}}$ with a being the lattice constant and ℓ being the magnetic length. To assess the contribution of this mechanism, we refer to the recent work by Wörnle *et al.* [58], where the domain wall profile was fitted without the mentioned contributions. The fit of the stray fields produced by the domain wall in Cr_2O_3 is very well described by the profile, which considers rotation of $\mathbf{L}$ only. This can be used as a hint that the contributions provided by the terms of Andreev [60] and Kabychenkov and Lisovskii [61] are not large enough to be seen in the NV measurements. Still, a more detailed investigation of these contributions should be done in the future.

F. Access to $T_{\text{N}}^{\text{bot}}$ via NV magnetometry

The strength of stray fields detected by the NV magnetometry consists of the uncompensated magnetization (magnetization of the one antiferromagnetic sublattice) at the top and bottom film surfaces, $M_{\text{top}} = (L/4)|_{z=t}$ and $M_{\text{bot}} = (L/4)|_{z=0}$. These sources should be supplemented by the thermally-induced magnetization (S33). To determine the paramagnetic phase transition of the film, we track the difference of the signals between the oppositely ordered antiferromagnetic domains, see Eq. (1, main text), , which vanishes when the film becomes paramagnetic. To determine the contribution from the oppositely ordered antiferromagnetic domains, we averaged the signal from red and blue domains within the window of the measurement.

At relatively low temperatures, $T < T_{\text{N}}^{\text{top}}$, the entire film is antiferromagnetically ordered. In this regime, we detect the magnetotransport signal (Fig. 2c of the main text). At $T > T_{\text{N}}^{\text{top}}$, only the uncompensated magnetization of the thin film and the bottom surface of the film contributes to B_{NV}. The thermally induced magnetization contributes to the stray fields up to $T = T_{\text{N}}^{\text{bot}}$, when the entire film becomes paramagnetic. In this way, the the magnitude of stray fields measured by NV magnetometry below the antiferromagnet-paramagnet phase transition reads

$$B_{\text{NV}}(T) = \underbrace{b_1\left[1-\left(\frac{T}{T_{\text{N}}^{\text{top}}+\zeta t}\right)^3\right]^{1/3}}_{\text{bottom surface}} + \underbrace{b_2\left[1-\left(\frac{T}{T_{\text{N}}^{\text{top}}}\right)^3\right]^{1/3}}_{\text{top surface}} + \underbrace{b_3\int_0^{t_{\text{AFM}}}|\mathbf{M}_\zeta(T,z)|\mathrm{d}z}_{\text{via reduction of }\mathbf{L}}, \quad \text{(4, main text)}$$

where $b_{1,2,3}$ are values that reflect the scaling of the NV signal (1, main text) with distance and are used as fitting parameters, see Supplementary Fig. 36. The last fitting parameter, ζ is used to determine the Néel temperature at the bottom film interface, $T_{\text{N}}^{\text{bot}} = T_{\text{N}}^{\text{top}} + \zeta t$ and respective scaling of M_ζ. There are the following constraints on the fitting parameters [67]. The values of b_1 and b_2 should be of opposite sign as they come from the oppositely magnetized (0 0 0 1) Cr_2O_3 surfaces. The parameter b_3 being explicitly written outside the integral simplifies the numerical fitting process. Alternatively, the scaling of $\mathbf{M}_\zeta$ with distance can be determined from the NV signal at low temperatures, where the influence of $\mathbf{M}_\zeta$ itself is negligible and provide an additional function of z under the integral. Thus, b_3 is limited by the absolute value of b_1. For the determination of ζ, we do not include the opposite directions of magnetic moments at the top and bottom surfaces into $b_{1,2}$ explicitly. Still, we note that the fits to the NV data (Fig. 3b,d and Supplementary Fig. 36) provide the expected opposite sign for the coefficients b_1 and b_2 , as well as $b_3 < b_1$ (Supplementary Fig. 52).

Having $T_{\text{N}}^{\text{top}}$ from the transport measurements (Supplementary Note 5), we determine $T_{\text{N}}^{\text{bot}}$ and parameters of the flexomagnetic contribution to the Néel temperature (S18), see Supplementary Table 3. The second and third columns in the table show the Néel temperature at the top and bottom surfaces of the film, respectively. Their values coincide for strain-free samples (single crystal and thin film with the thickness of 250 nm) and the homogeneously strained thin film with the thickness of 100 nm. We note that the NV data taken of the 30-nm-thick sample contains very limited number of datapoints above the $T = T_{\text{N}}^{\text{top}}$ because $T_{\text{N}}^{\text{top}}$ is close to the maximal achievable temperature in the NV magnetometer. Therefore, to estimate the $T_{\text{N}}^{\text{bot}}$ for the 30-nm-thick sample, we used ζ determined for the 50-nm-thick sample (Supplementary Fig. 52c; Supplementary Table 3).

We note that NV imaging is an indirect method to measure magnetization. The latter is necessary for our approach to access the interior of the film to address thermally induced flexomagnetism. Namely, the Fourier map of stray

fields integrated from the sample of thickness t reads

$$B(\mathbf{k}) = \int\limits_0^t B_0(\mathbf{k},z) k e^{-k(d+t-z)} \mathrm{d}z,$$
$$B_0(\mathbf{k},z) = \frac{\mu_0 M_\mathrm{S}(z)}{2} \begin{pmatrix} -\frac{k_x^2}{k^2} & -\frac{1}{2}\sin\left(2\arctan\frac{k_y}{k_x}\right) & -\frac{ik_x}{k} \\ -\frac{1}{2}\sin\left(2\arctan\frac{k_y}{k_x}\right) & -\frac{k_y^2}{k^2} & -\frac{ik_y}{k} \\ -\frac{ik_x}{k} & -\frac{ik_y}{k} & 1 \end{pmatrix} \times \begin{pmatrix} m_x(\mathbf{k},z) \\ m_y(\mathbf{k},z) \\ m_z(\mathbf{k},z) \end{pmatrix}, \tag{S35}$$

where $\mathbf{k} = \{k_x, k_y, 0\}$ with $k = |\mathbf{k}|$ is the wave vector of the Fourier transform, d is the distance from the top surface to the NV tip, $M_\mathrm{S}(z)$ and $\mathbf{m}(\mathbf{k},z) = \{m_x, m_y, m_z\}$ are the saturation magnetization and the unit vector characterizing the magnetic texture at the distance z from the bottom film interface, respectively. The exact shape of $\mathbf{m}(\mathbf{k},z)$ is not known in contrast to the problems, where the stray fields of a single skyrmion or a domain wall are considered. That's why we use an approximation stated as Eq. (5) of the main text, which contains the key contributions to the measured value of stray fields.

To unambiguously solve the inverse problem of finding the magnetization from the measured stray fields distribution above the sample's surface, one needs to access a 3D distribution of the Néel order parameter at different temperatures, which is not established for antiferromagnets. Being available, such a technique could be able to provide a direct measurement of both, $T_\mathrm{N}^\mathrm{top}$ and $T_\mathrm{N}^\mathrm{bot} = T_\mathrm{N}^\mathrm{top} + \zeta t$ by a sequence of measurements of 3D domain structure. In this case, the gradient of the Néel temperature and thermal contribution to the flexomagnetic effect can be accessed directly without the need of combination of magnetotransport and NV magnetometry.

Supplementary Figures

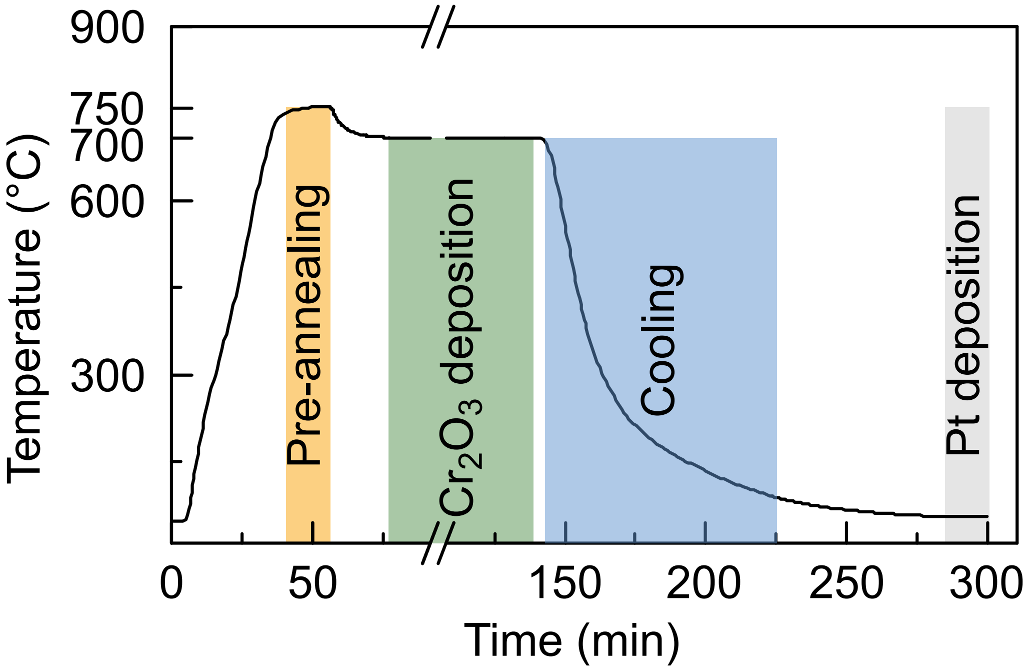

Supplementary Fig. 1. **Time evolution of the temperature during the deposition process of Cr_2O_3 films.** The graph shows time variation of the temperature during the sample fabrication with relevant processing steps highlighted with color bars (pre-annealing, Cr_2O_3 deposition, cooling, Pt electrode deposition). Prior to the deposition, a Al_2O_3 substrate is heated to 750°C under Ar gas pressure of 5×10^{-3} mbar. The substrate is annealed at 750°C for about 15 min to clean off adsorbates and improve adhesion of the film. Further, the substrate is cooled down to 700°C, at which Cr_2O_3 is deposited. With the deposition rate of 1.72 nm min^{-1}, the growth of a 50-nm-thick film takes about 30 min. A high deposition temperature ensures the coherent epitaxial growth of Cr_2O_3 on (0 0 0 1) sapphire substrates. After the deposition, the sample cools down with a cooling rate of 25°C min^{-1} under the same argon atmosphere. A thin film Pt electrode is deposited at room temperature without breaking the vacuum.

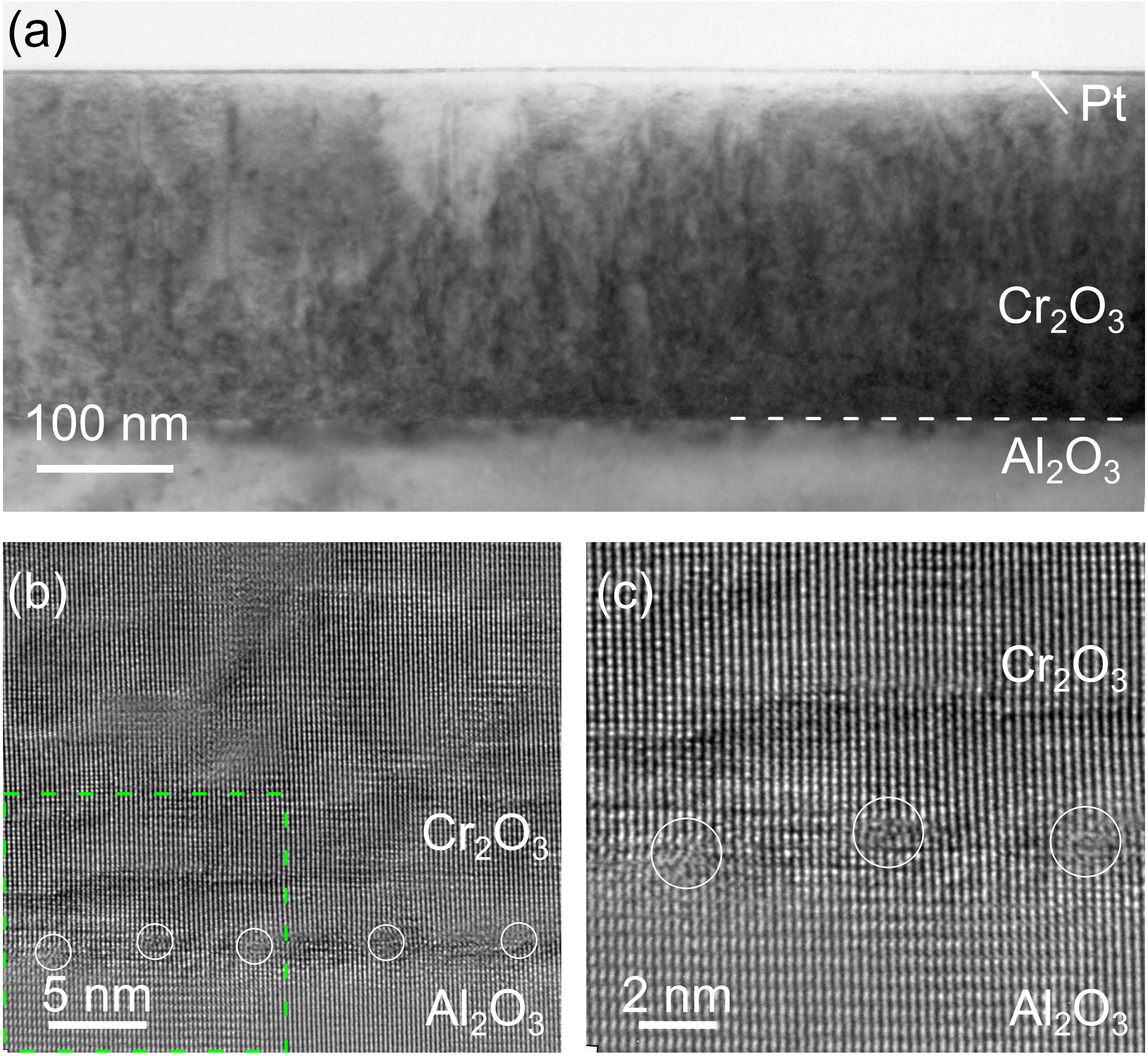

Supplementary Fig. 2. **Transmission electron microscopy imaging of a 250-nm-thick Cr_2O_3 thin film.** (a) TEM image of a 250-nm-thick Cr_2O_3 thin film prepared by magnetron sputtering. The dashed horizontal line indicates the interface between the Cr_2O_3 thin film and the Al_2O_3 substrate. The position of the Pt top layer is indicated as well. (b) High-resolution TEM image of the Al_2O_3/Cr_2O_3 interface region. Misfit dislocations distributed at the interface with a period of about 5 nm are highlighted with circles. (c) A close-up image of the Al_2O_3/Cr_2O_3 interface region, highlighted with a green dashed box in panel (b).

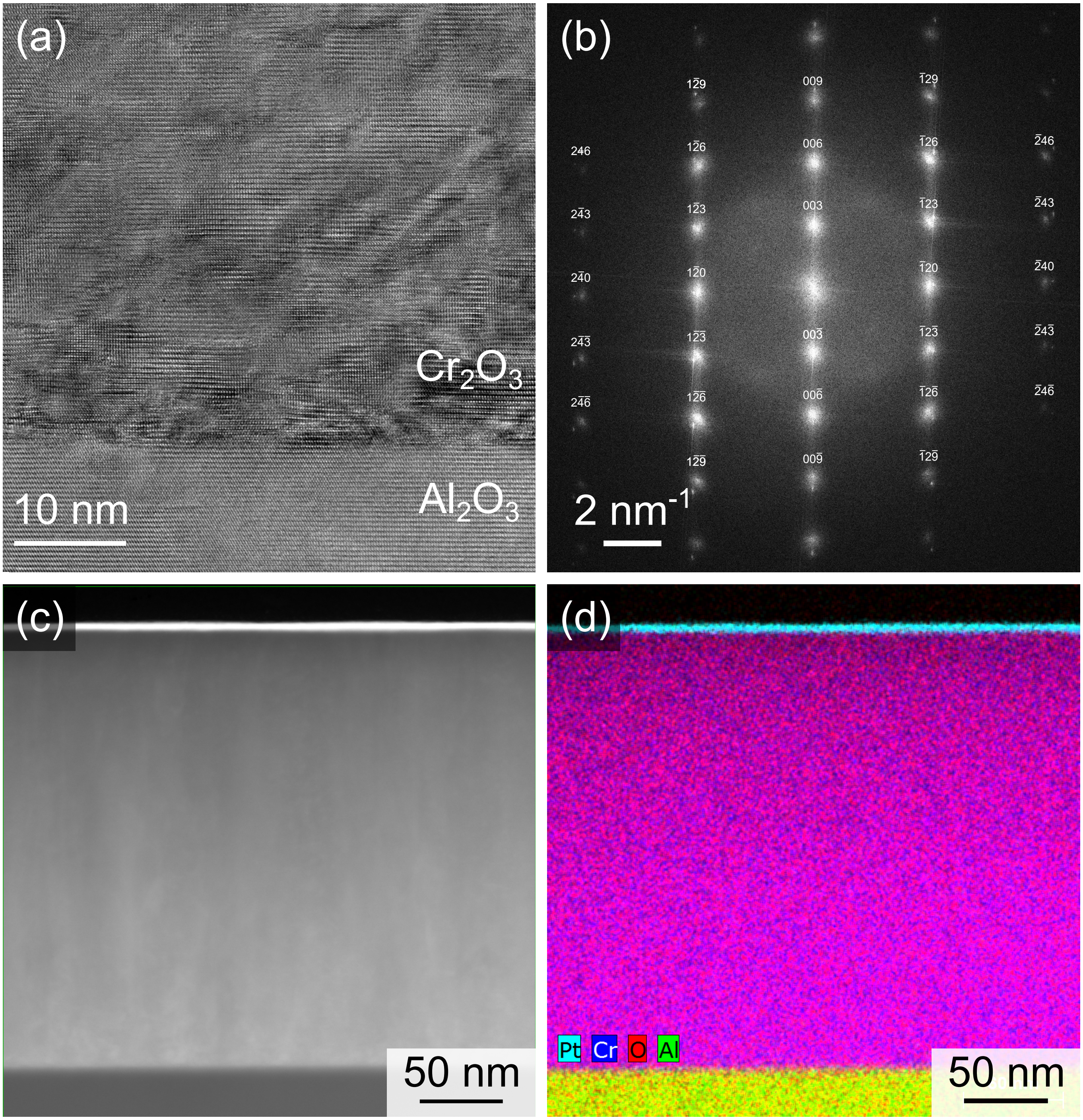

Supplementary Fig. 3. **Transmission electron microscopy imaging of a 250-nm-thick Cr_2O_3 thin film.** (a) High-resolution TEM image of the Cr_2O_3/Al_2O_3 interface. (b) The fast Fourier transform (FFT) of the image from panel (a) confirms the phase purity and epitaxial relation of the Cr_2O_3 thin film and the Al_2O_3 substrate. Indexing is based on the corundum structure in [2 1 0] zone axis geometry. (c) HAADF-STEM image of a 250-nm-thick Cr_2O_3 thin film sample and (d) the corresponding EDX-based element map recorded for the region shown in panel (c). No other chemical elements are identified, except for Al, Cr, O, and Pt. No intermixing at the Cr_2O_3/Pt and Cr_2O_3/Al_2O_3 interfaces is observed.

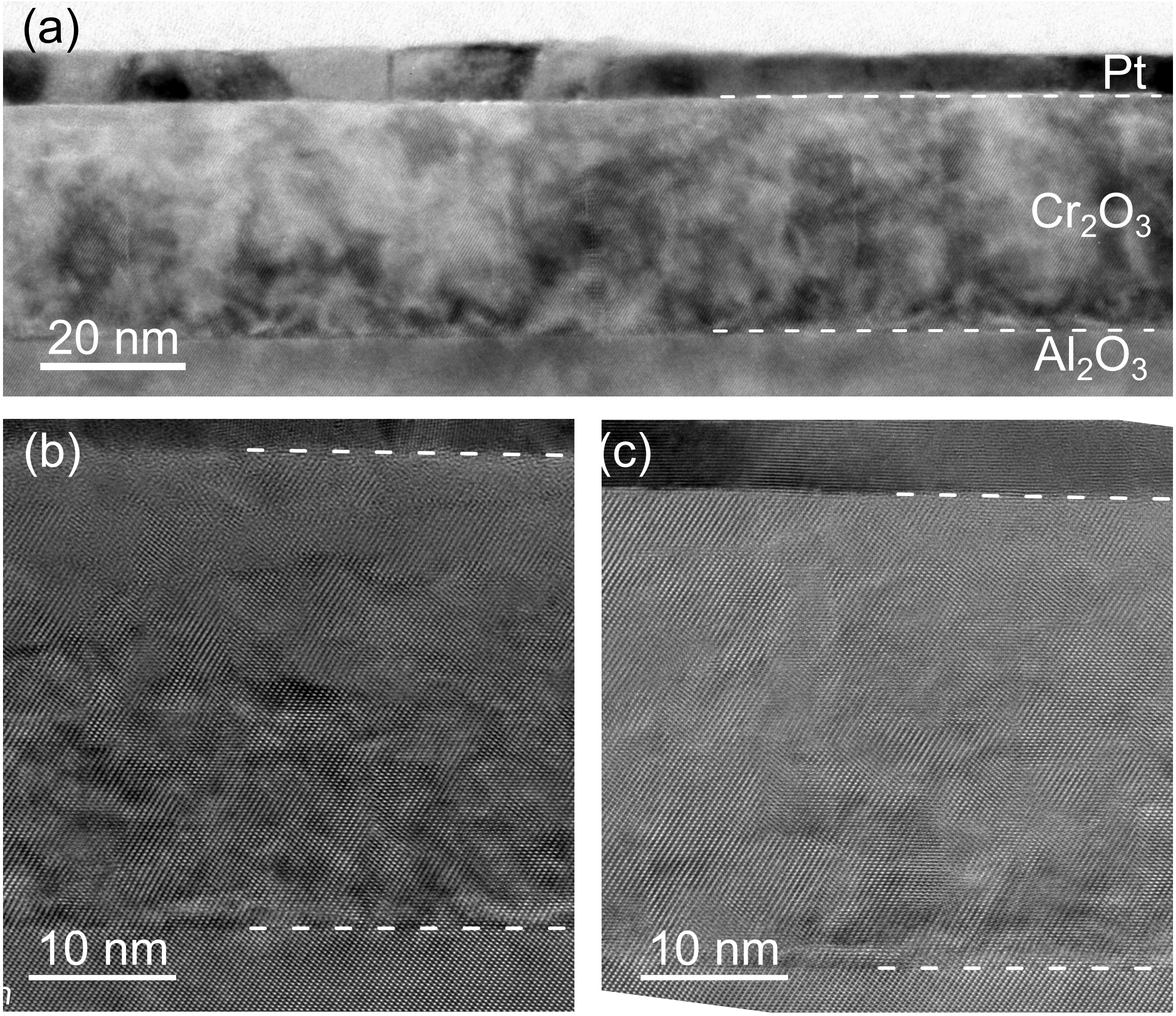

Supplementary Fig. 4. **Transmission electron microscopy imaging of a 30-nm-thick Cr_2O_3 thin film.** (a-c) TEM images of a 30-nm-thick Cr_2O_3 thin film. The dashed horizontal lines indicate the interface between the Cr_2O_3 thin film and the Al_2O_3 substrate, as well as the Cr_2O_3 thin film and the Pt top layer.

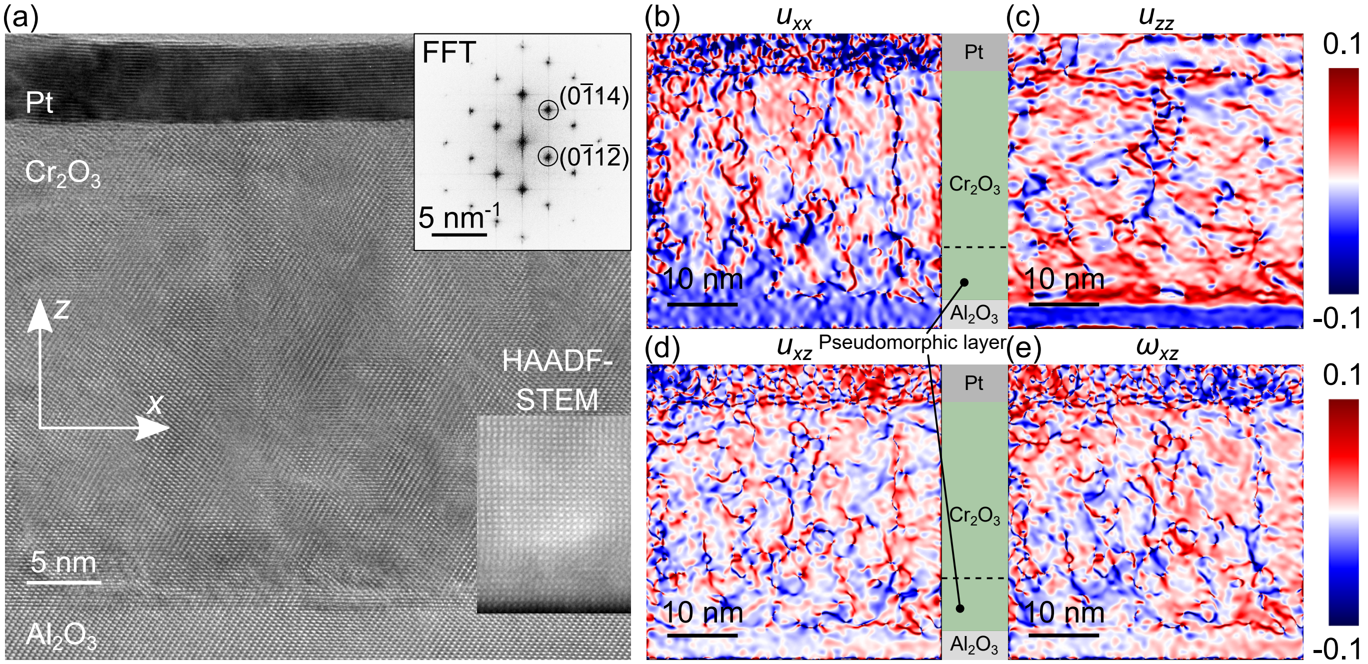

Supplementary Fig. 5. **High-resolution TEM analysis of the strain state in a 30-nm-thick Cr_2O_3 thin film.** (a) Large field of view high-resolution TEM image taken at $(2\,\bar{1}\,\bar{1}\,0)$ zone axis. Top-right inset displays the Fourier transform, including the two systematic reflections $(0\,\bar{1}\,1\,\bar{2})$ and $(0\,\bar{1}\,1\,4)$ employed for geometric phase strain analysis. Bottom-right inset shows a high-resolution HAADF-STEM image taken at $(1\,2\,\bar{3}\,0)$ crystal orientation of the interface region. Note, the distortion of the atomic column positions in the lower part of the HAADF-STEM image are due to image drift. (b-e) 2D strain maps of the region displayed in (a), representing different components of the strain tensor: (b,c) diagonal components u_{xx} and u_{zz}, (d) shear component u_{xz} and (e) rotation component w_{xz}. Lines of diverging strain in the strain maps are indicated with circles.

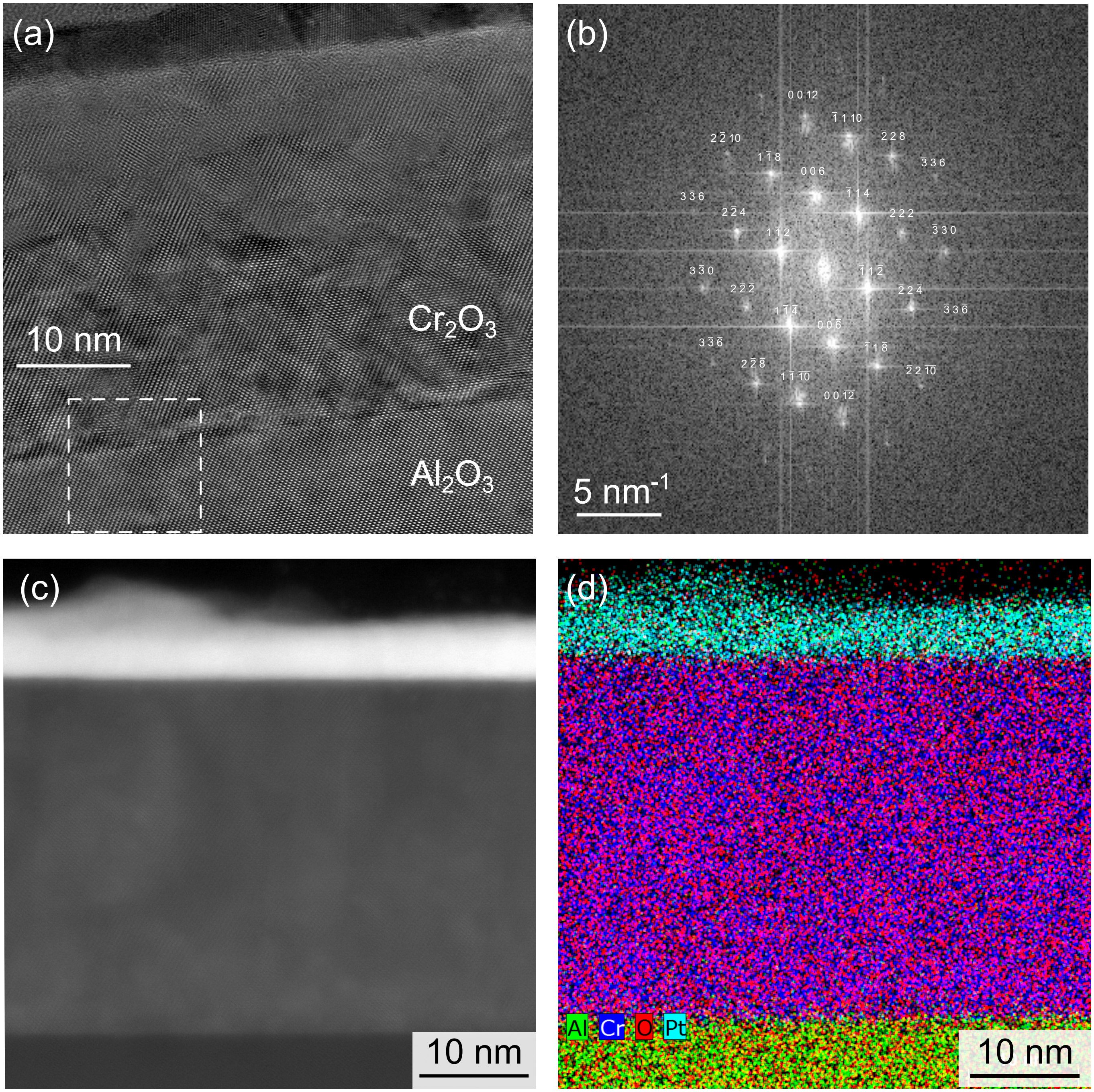

Supplementary Fig. 6. **Transmission electron microscopy imaging of a 30-nm-thick Cr_2O_3 thin film.** (a) High-resolution TEM image of a 30-nm-thick Cr_2O_3 thin film prepared by magnetron sputtering. (b) The FFT of the Cr_2O_3/Al_2O_3 interface region highlighted with white dashed box in panel (a) confirms the phase purity and epitaxial relation of the Cr_2O_3 thin film and the Al_2O_3 substrate. Indexing is based on the corundum structure in [1 1 0] zone axis geometry. (c) HAADF-STEM image and (d) the corresponding EDX-based element map for the region shown in panel (c). No other chemical elements are identified, except for Al, Cr, O, and Pt. No intermixing at the Cr_2O_3/Pt and Cr_2O_3/Al_2O_3 interfaces is observed.

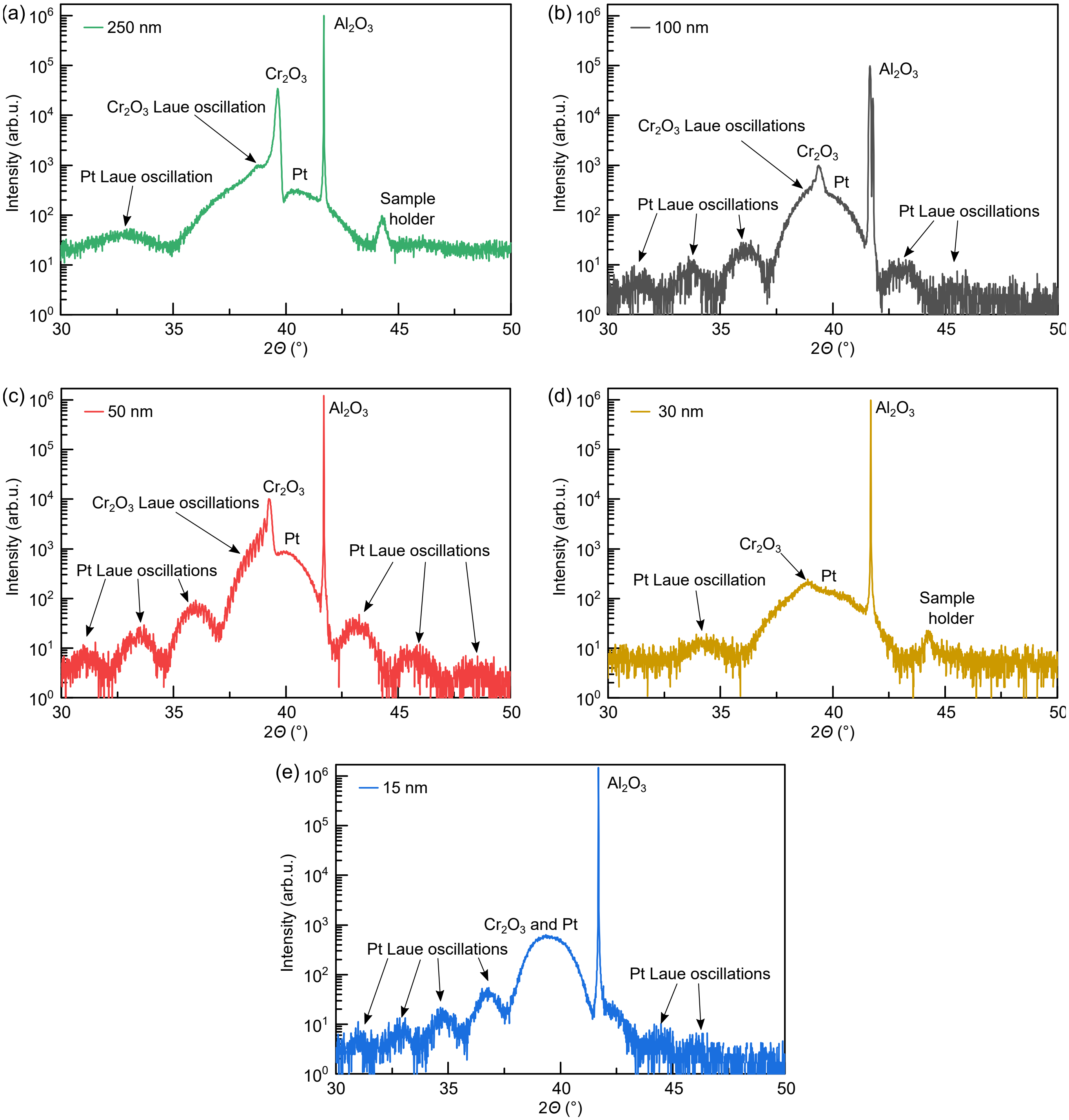

Supplementary Fig. 7. **Phase purity of the Cr_2O_3 thin films prepared on Al_2O_3 single crystalline substrates.** (a-e) X-ray diffraction patterns of the Cr_2O_3 thin films of different thickness measured around the (0 0 0 6) reflection. The X-ray diffraction patterns reveal broad reflections from the Cr_2O_3 and Pt thin films, as well as a sharp reflection from the Al_2O_3 substrate. Positions of Laue oscillations of Cr_2O_3 and Pt layers are indicated with arrows.

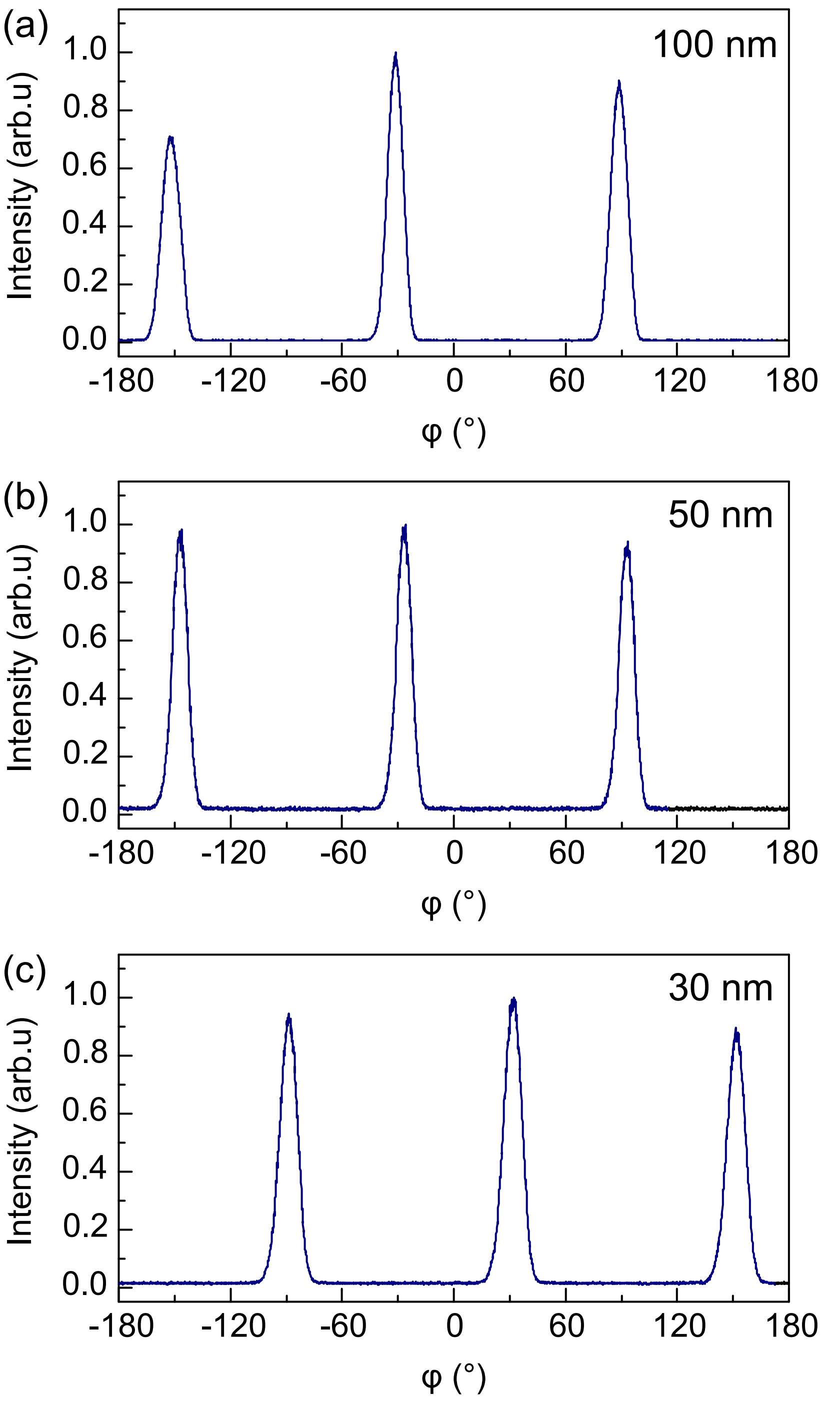

Supplementary Fig. 8. ϕ **scans of the** $(10\bar{1}10)$ **reflections of $\mathbf{Cr_2O_3}$ magnetron sputtered thin films.** The presence of only three peaks is in accordance with the three-fold symmetry (for rhombohedral syngony) of the [0 0 0 1] axis, confirming epitaxial growth of the Cr_2O_3 thin film on the Al_2O_3 substrate.

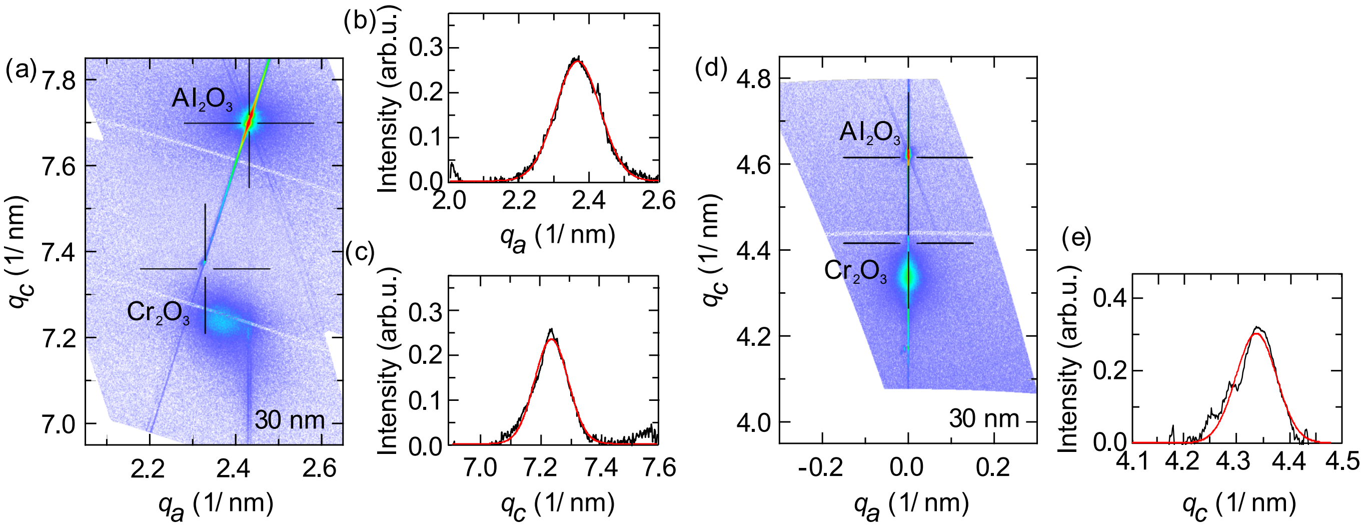

Supplementary Fig. 9. **Calculation of the average lattice parameters for 30-nm-thick Cr_2O_3 film.** (a) Experimental RSM data of the asymmetric $(1\,0\,\bar{1}\,10)$ reflection. Line scans of the averaged intensity of the $(1\,0\,\bar{1}\,10)$ reflection along the q_a and q_c axes are shown in panels (b) and (c), respectively (integration includes the rectangular region around Cr_2O_3 peak). The line scans are fitted using a single Gaussian function to extract the central position of the largest peak, which is taken as the average values of $q_{a,\mathrm{av}}$ and $q_{c,\mathrm{av}}$. The average value $q_{a,\mathrm{av}}$ is used to determine the average in-plane lattice parameter a_{av} (Fig. 1f of the main text). The average value $q_{c,\mathrm{av}}$ is used to determine the average out-of-plane lattice parameter c_{av} (Fig. 1g of the main text). (d) Experimental RSM data of the symmetric $(0\,0\,0\,6)$ reflection. (e) The line scan of the averaged intensity of the $(0\,0\,0\,6)$ reflection along the q_c axis. The line scan is fitted using a single Gaussian function to extract the central position of the peak, which is taken as the average value of $q_{c,\mathrm{av}}$. The average value $q_{c,\mathrm{av}}$ is used to determine the average out-of-plane lattice parameter c_{av} (Fig. 1g of the main text). The values of c_{av} determined from the analysis of both reflections coincide. We note that the pseudomorphic layer is excluded from this analysis. Black crosses in panels (a) and (d) represent positions of the corresponding reflections in the strain-free material.

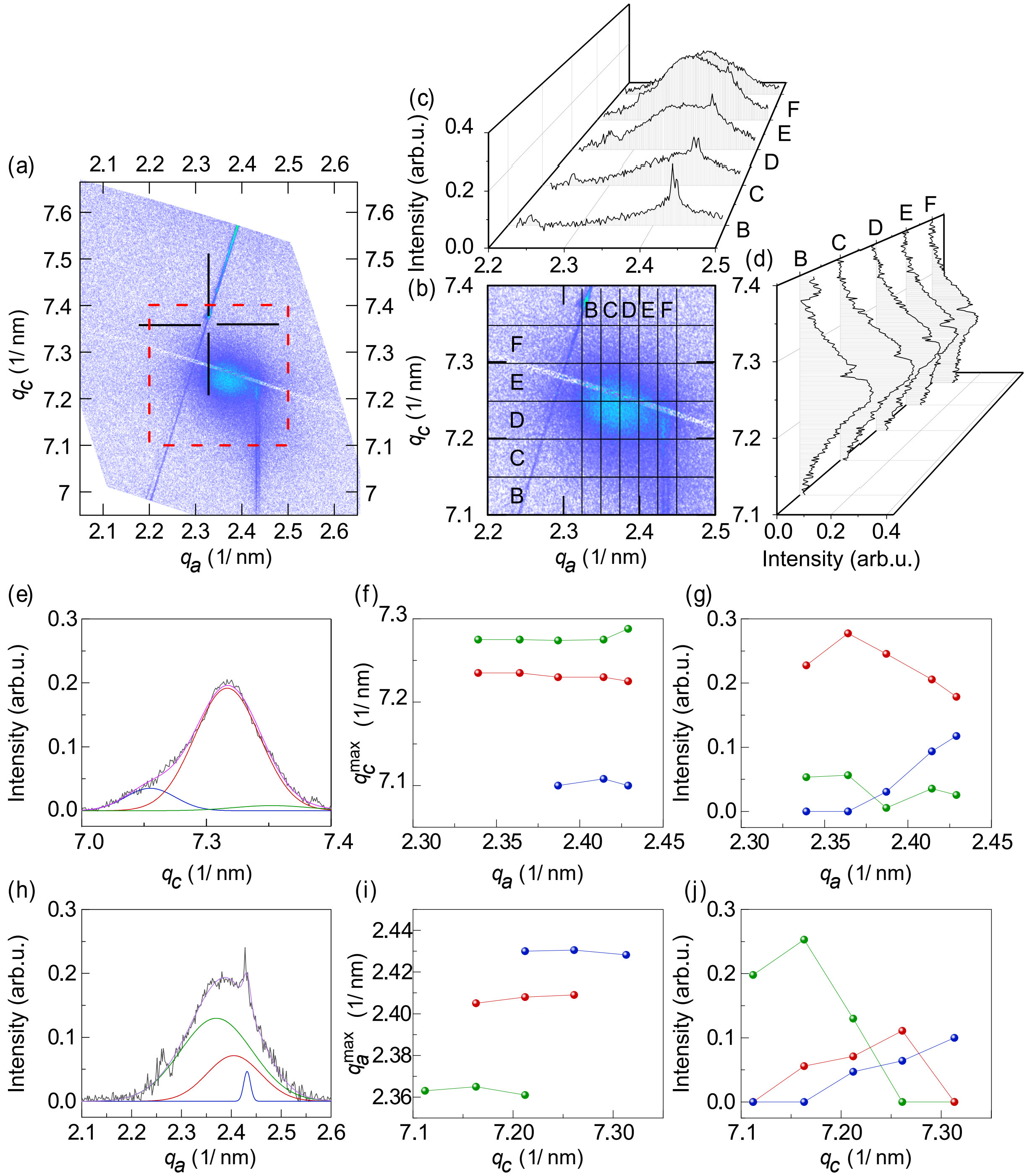

Supplementary Fig. 10. **Structural inhomogeneity of the 30-nm-thick Cr_2O_3 thin film.** (a) Experimental RSM image of the asymmetric $(10\bar{1}10)$ reflection. The area, which is used for this analysis, is indicated with a red box. We note that it includes also the pseudomorphic layer (vertical streak parallel to the q_c axis at q_a of about 2.43 nm^{-1}). Black cross represents position of the corresponding reflection in the strain-free Cr_2O_3 material. The reflection is analyzed by slicing it along the q_a and q_c axes along the black lines shown in panel (b). The interslice separation is 0.025 nm^{-1} along the q_a axis and 0.05 nm^{-1} along the q_c axis. Each slice is indicated with a capital letter. (c) The set of line scans showing the change of the intensity of the reflection within the slice area along the q_a axis. (d) The set of line scans showing the change of the intensity of the reflection within the slice area along the q_c axis. The line scans of the averaged intensity of the $(10\bar{1}10)$ reflection along q_a and q_c axes are shown with black curves in panels (e) and (h), respectively. Each of the line scans (black curves) in panels (c) and (d) are fitted using a multipeak Gaussian function (S1). The best fit to the experimental data can be done with 3 Gaussian peaks, which are indicated with red, green and blue curves in panels (e) and (h). The q_a and q_c positions corresponding to the centers of the three peaks are used as a guide to fit the line scans shown in panels (c) and (d), respectively. The triple Gaussian fit to the data shown in (c) and (d) allows to determine the peak intensity, position and width. While the position of each of the three peaks is found to be the same for all slices (f) and (i), the intensity contribution of each of the peaks to the total intensity is changed. The intensity of each of the three peaks for different q_a and q_c are shown for different slices in panels (g) and (j), respectively.

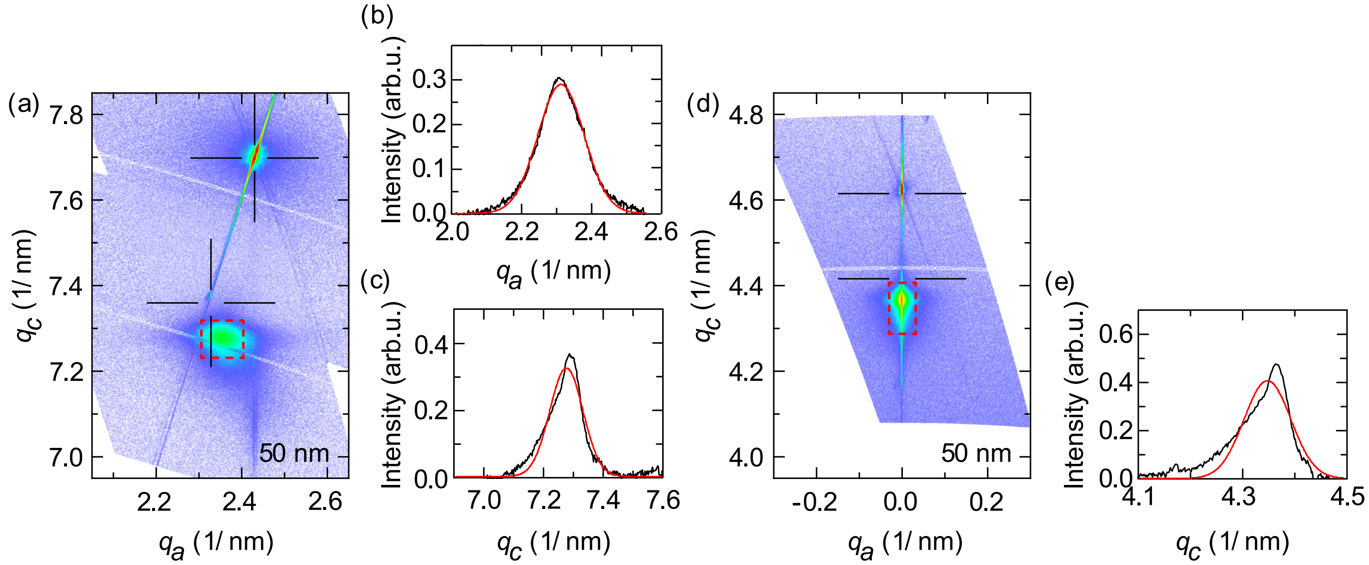

Supplementary Fig. 11. **Calculation of the average lattice parameters for 50-nm-thick Cr_2O_3 film.** (a) Experimental RSM data of the asymmetric $(1\,0\,\bar{1}\,10)$ reflection. Line scans of the averaged intensity of the $(1\,0\,\bar{1}\,10)$ reflection along the q_a and q_c axes are shown in panels (b) and (c), respectively (integration includes the rectangular region around Cr_2O_3 peak). The line scans are fitted using a single Gaussian function to extract the central position of the largest peak, which is taken as the average values of $q_{a,\mathrm{av}}$ and $q_{c,\mathrm{av}}$. The average value $q_{a,\mathrm{av}}$ is used to determine the average in-plane lattice parameter a_{av} (Fig. 1f of the main text). The average value $q_{c,\mathrm{av}}$ is used to determine the average out-of-plane lattice parameter c_{av} (Fig. 1g of the main text). (d) Experimental RSM data of the symmetric $(0\,0\,0\,6)$ reflection. (e) The line scan of the averaged intensity of the $(0\,0\,0\,6)$ reflection along the q_c axis. The line scan is fitted using a single Gaussian function to extract the central position of the peak, which is taken as the average value of $q_{c,\mathrm{av}}$. The average value $q_{c,\mathrm{av}}$ is used to determine the average out-of-plane lattice parameter c_{av} (Fig. 1g of the main text). The values of c_{av} determined from the analysis of both reflections coincide. We note that the pseudomorphic layer is excluded from this analysis. Black crosses in panels (a) and (d) represent positions of the corresponding reflections in the strain-free material.

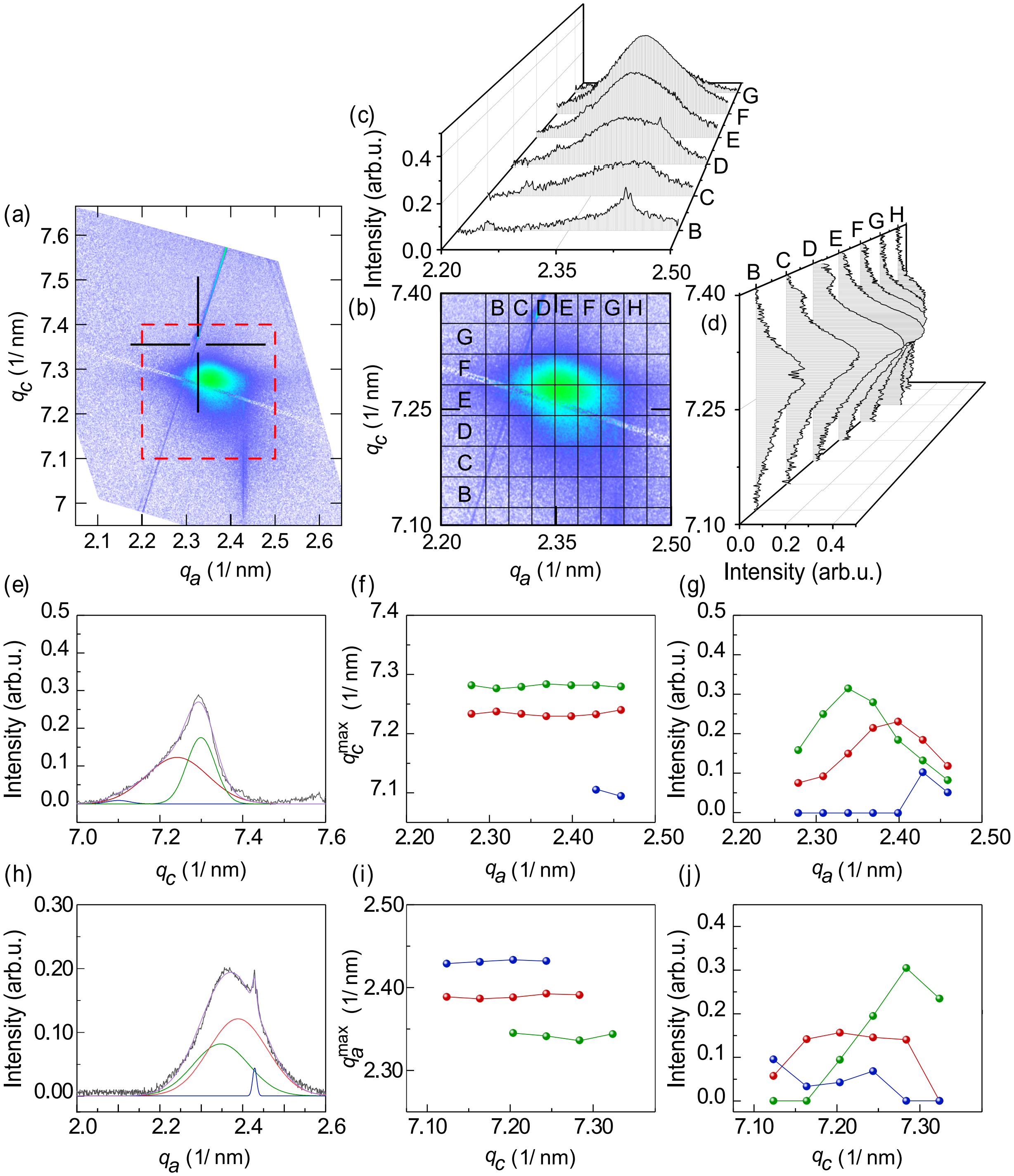

Supplementary Fig. 12. **Structural inhomogeneity of the 50-nm-thick Cr_2O_3 thin film.** (a) Experimental RSM image of the asymmetric $(1\,0\,\bar{1}\,10)$ reflection. The area, which is used for this analysis, is indicated with a red box. We note that it includes also the pseudomorphic layer (vertical streak parallel to the q_c axis at q_a of about 2.43 nm^{-1}). Black cross represents position of the corresponding reflection in the strain-free Cr_2O_3 material. The reflection is analyzed by slicing it along the q_a and q_c axes along the black lines shown in panel (b). The interslice separation is 0.03 nm^{-1} along the q_a axis and 0.04 nm^{-1} along the q_c axis. Each slice is indicated with a capital letter. (c) The set of line scans showing the change of the intensity of the reflection within the slice area along the q_a axis. (d) The set of line scans showing the change of the intensity of the within the slice area along the q_c axis. The line scans of the averaged intensity of the $(1\,0\,\bar{1}\,10)$ reflection along q_a and q_c axes are shown with black curves in panels (e) and (h), respectively. Each of the line scans (black curves) in panels (c) and (d) are fitted using a multipeak Gaussian function (S1). The best fit to the experimental data can be done with 3 Gaussian peaks, which are indicated with red, green and blue curves in panels (e) and (h). The triple Gaussian fit to the data shown in (c) and (d) allows to determine the peak intensity, position and width. While the position of each of the three peaks is found to be the same for all slices (f) and (i), the intensity contribution of each of the peaks to the total intensity is changing. The intensity of each of the three peaks for different q_a and q_c are shown for different slices in panels (g) and (j), respectively.

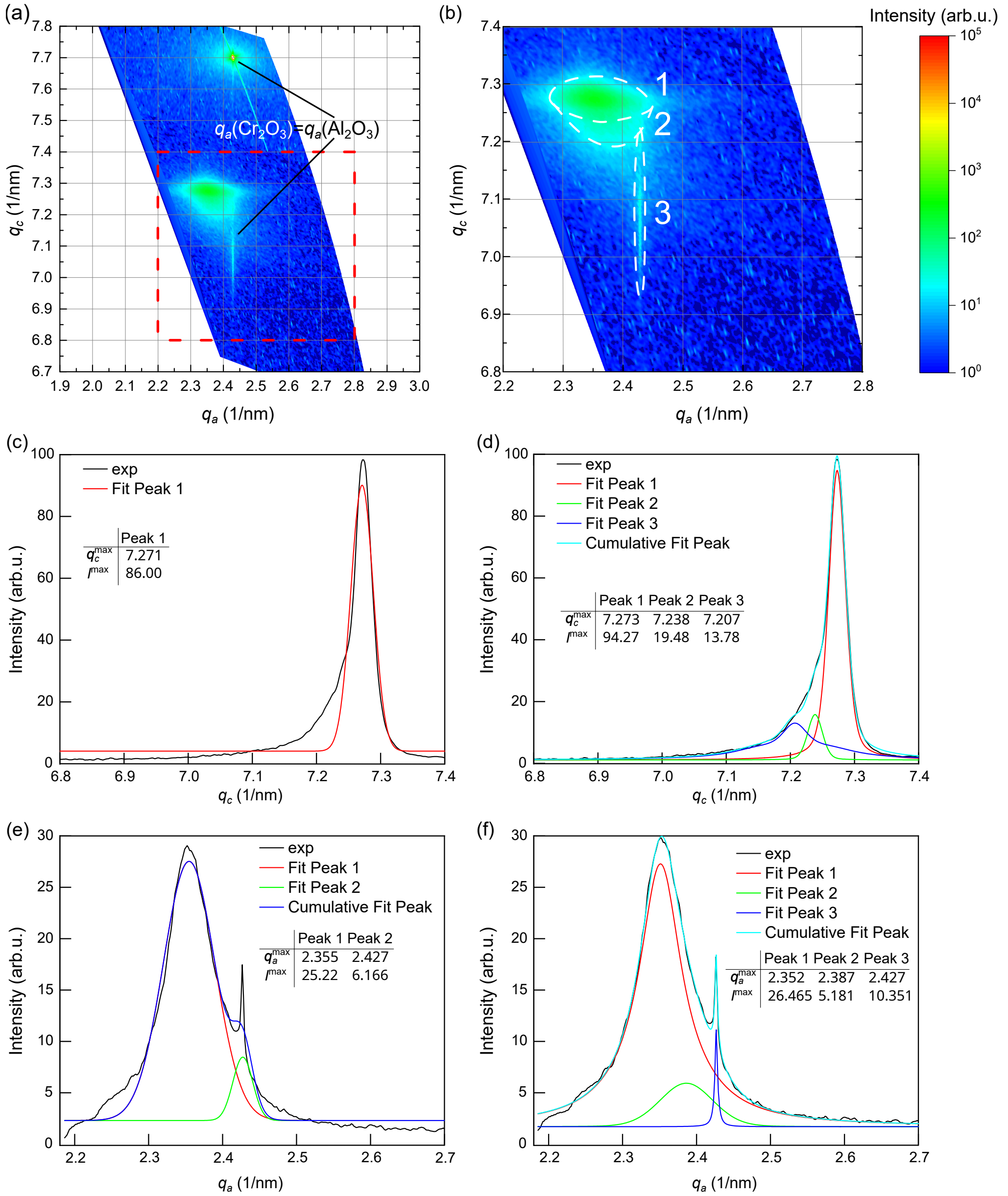

Supplementary Fig. 13. **Calculation of the lattice parameters for 50-nm-thick Cr_2O_3 film.** (a) Experimental RSM data of the asymmetric $(1\,0\,\bar{1}\,10)$ reflection taken of thin film with the thickness of 50 nm. (b) A close up view of the RSM region highlighted with a red dashed box in panel (a). The asymmetric appearance of the reflection suggests the presence of different structural zones within the thickness of the thin film (indicated with dashed lines in panel (b)): Zone 1 – main part of the sample; Zone 2 – intermediate highly strained zone close to the pseudomorphic layer; Zone 3 – pseudomorphic layer. Line scans of the averaged intensity of the $(1\,0\,\bar{1}\,10)$ reflection along the q_c and q_a axes are shown in panels (c-d) and (e-f), respectively. The line scans are fitted using a single Gaussian function to extract the central position of the peak, which is taken as the average values of $q_{c,\text{av}}$ and a double Gaussian function to extract the position of the peaks, which is taken as the average values of $q_{a,\text{av}}$ and position of the pseuomorphic layer peak, which is taken as the values of $q_{a,PM}$. The average value $q_{a,\text{av}}$ is used to determine the average in-plane lattice parameter a_{av} (Fig. 1f of the main text and Supplementary Fig. 26a). The average value $q_{c,\text{av}}$ is used to determine the average out-of-plane lattice parameter c_{av} (Fig. 1g of the main text and Supplementary Fig. 26b). We note that it includes also the pseudomorphic layer (vertical streak parallel to the q_c axis at q_a of about 2.427 nm^{-1}). In addition, each of the line scans (black curves) in panels (c) and (e) are fitted using a multipeak Gaussian function (S1). The best fit to the experimental data can be done with three Gaussian peaks, which are indicated with red, green and blue curves in panels (d) and (f) and corresponds to the evidence of the three different zones in the thin film. The area, which is used for this analysis, is indicated with a red dashed box. Dotted line shows the peak position of the pseudomorphic layer. The fit results (peak position and intensity) are shown as a table in each panel.

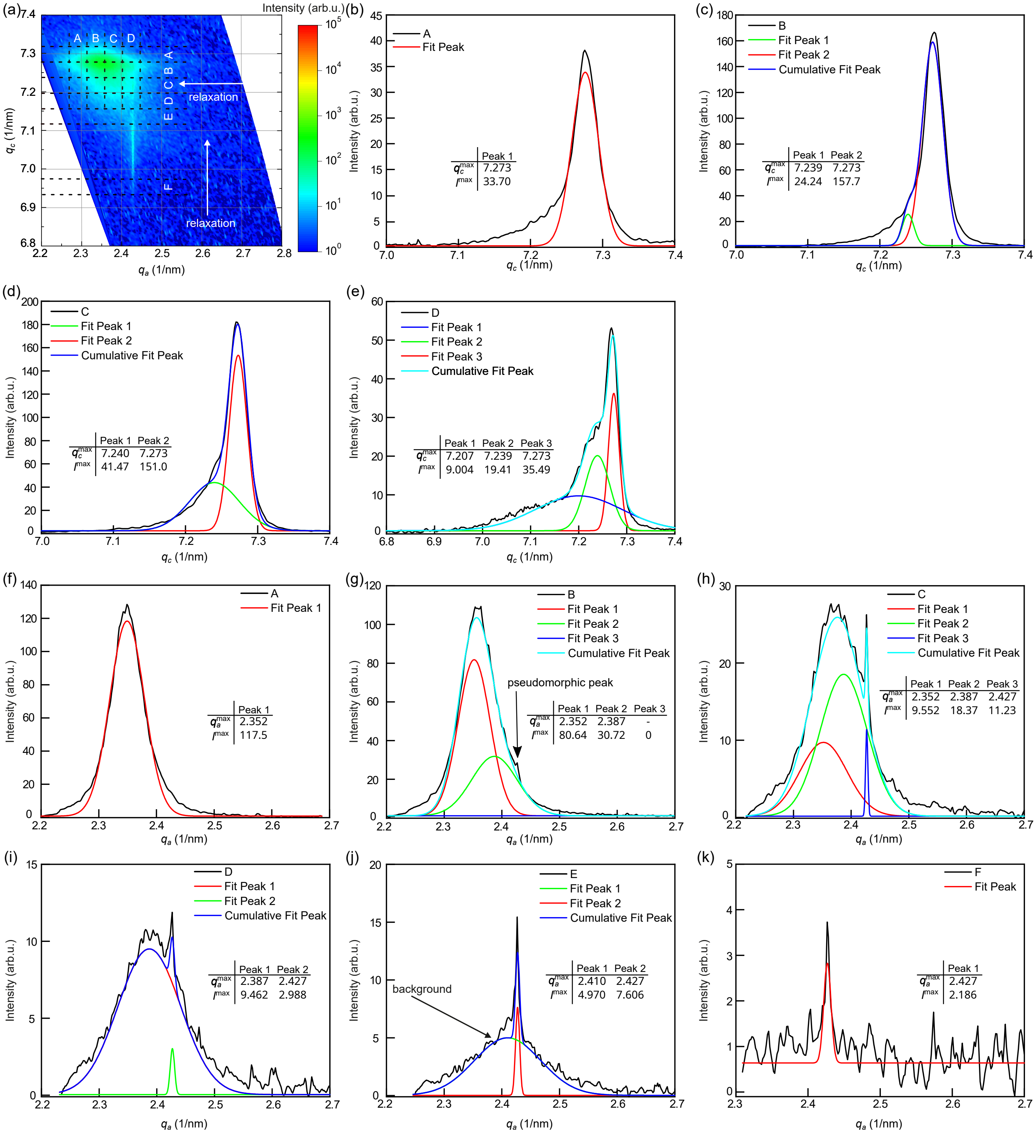

Supplementary Fig. 14. **Structural inhomogeneity of the 50-nm-thick Cr_2O_3 thin film** (a) Part of the experimental RSM image of the asymmetric $(10\bar{1}10)$ reflection. The area, which is used for this analysis, is indicated with a red dashed box in Supplementary Fig. 13. The reflection is analyzed by slicing it along the q_a and q_c axes along the black dotted lines shown in panel (a), respectively. The interslice separation is $0.04\,\mathrm{nm}^{-1}$ along the q_a axis and $0.04\,\mathrm{nm}^{-1}$ along the q_c axis. Each slice is indicated with a capital letter. The set of line scans of the averaged intensity of the $(10\bar{1}10)$ reflection along q_c and q_a axes are shown in panels (b-e) and (f-k), respectively. Each of the line scans (black curves) in panels (b-e) and (f-k) are fitted using a multipeak Gaussian function (S1) with the fixed q_a and q_c values obtained in Supplementary Fig.13d and f. The fit results (peak position and intensity) are shown as a table in each panel.

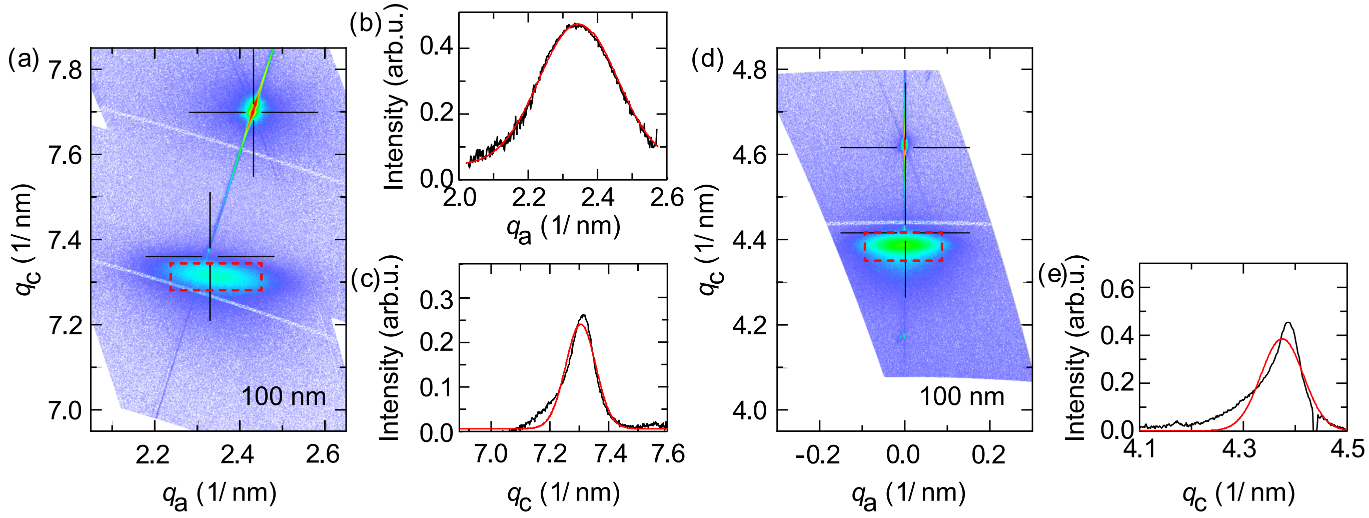

Supplementary Fig. 15. **Calculation of the average lattice parameters for 100-nm-thick Cr_2O_3 film.** (a) Experimental RSM data of the asymmetric $(1\,0\,\bar{1}\,10)$ reflection. Line scans of the averaged intensity of the $(1\,0\,\bar{1}\,10)$ reflection along the q_a and q_c axes are shown in panels (b) and (c), respectively (integration includes the rectangular region around Cr_2O_3 peak). The line scans are fitted using a single Gaussian function to extract the central position of the largest peak, which is taken as the average values of $q_{a,\mathrm{av}}$ and $q_{c,\mathrm{av}}$. The average value $q_{a,\mathrm{av}}$ is used to determine the average in-plane lattice parameter a_{av} (Fig. 1f of the main text). The average value $q_{c,\mathrm{av}}$ is used to determine the average out-of-plane lattice parameter c_{av} (Fig. 1g of the main text). (d) Experimental RSM data of the symmetric $(0\,0\,0\,6)$ reflection. (e) The line scan of the averaged intensity of the $(0\,0\,0\,6)$ reflection along the q_c axis. The line scan is fitted using a single Gaussian function to extract the central position of the peak, which is taken as the average value of $q_{c,\mathrm{av}}$. The average value $q_{c,\mathrm{av}}$ is used to determine the average out-of-plane lattice parameter c_{av} (Fig. 1g of the main text). The values of c_{av} determined from the analysis of both reflections coincide. Black crosses in panels (a) and (d) represent positions of the corresponding reflections in the strain-free material.

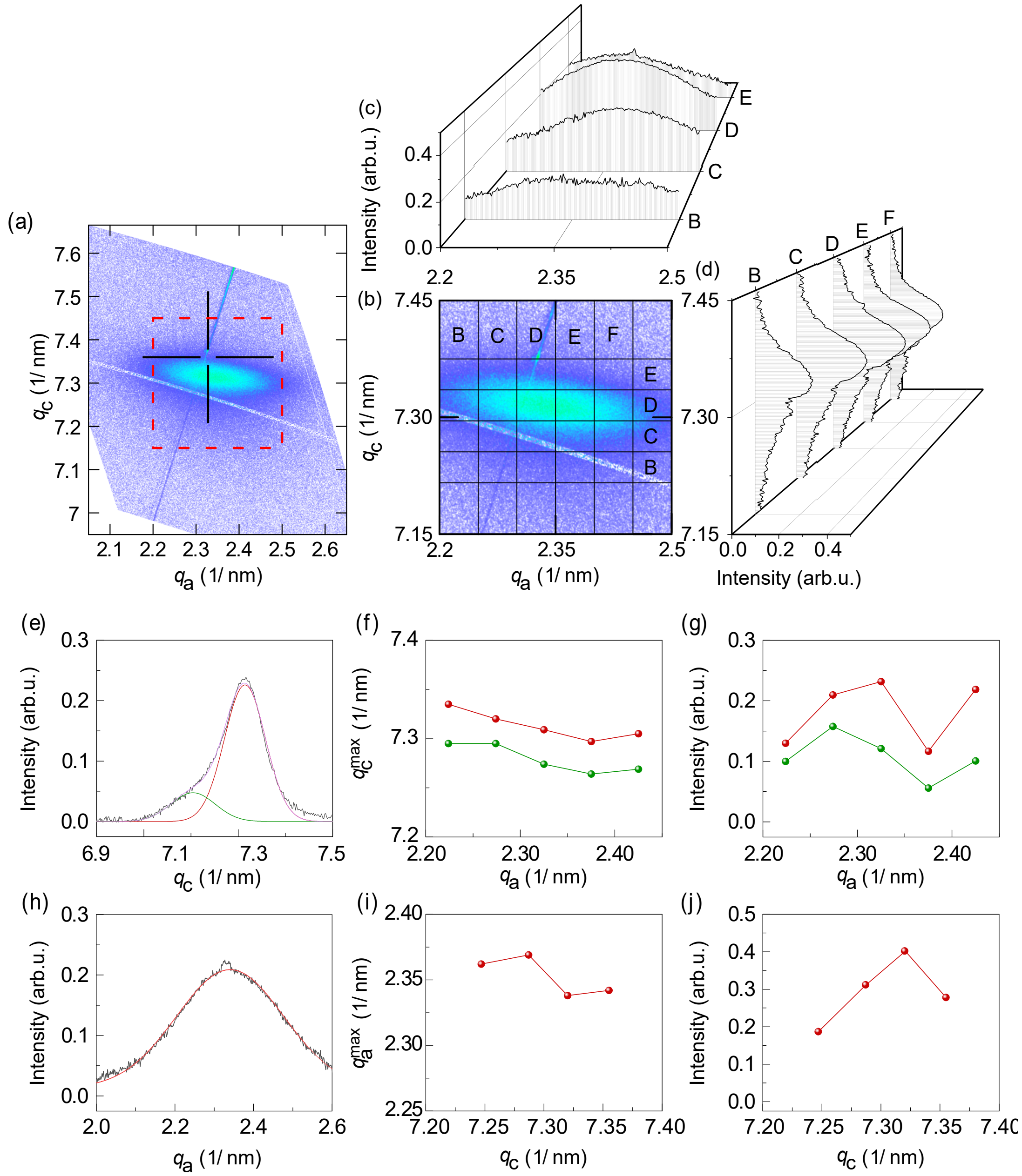

Supplementary Fig. 16. **Structural inhomogeneity of the 100-nm-thick Cr_2O_3 thin film.** (a) Experimental RSM image of the asymmetric $(1\,0\,\bar{1}\,10)$ reflection. The area, which is used for this analysis, is indicated with a red box. We note that for this sample we do not observe a pseudomorphic layer. Black cross in panel represents position of the corresponding reflection in the strain-free Cr_2O_3 material. The reflection is analyzed by slicing it along the q_a and q_c axes along the black lines shown in panel (b). The interslice separation is 0.05 nm^{-1} along the q_a axis and 0.03 nm^{-1} along the q_c axis. Each slice is indicated with a capital letter. (c) The set of line scans showing the change of the intensity of the reflection within the slice area along the q_a axis. (d) The set of line scans showing the change of the intensity of the within the slice area along the q_c axis. The line scans of the averaged intensity of the $(1\,0\,\bar{1}\,10)$ reflection along q_c and q_a axes are shown with black curves in panels (e) and (h), respectively. Each of the line scans (black curves) in panels (c) and (d) are fitted using a multipeak Gaussian function (S1). The best fit to the experimental data can be done with 1 Gaussian peak for q_a (red curve) and 2 Gaussian peaks for q_c (red and blue curves). The Gaussian fit to the data shown in panel (c) with a single peak and in panel (d) with a double peak allows to determine the peak intensity, position and width from the fitting process. In contrast to the thinner films (Supplementary Figs. 10 and 12), not only the intensity but also the position of the peaks is changing (panels (f) and (i)). The intensity of each of the peaks at different q_a and q_c are shown for different slices in panels (g) and (j), respectively.

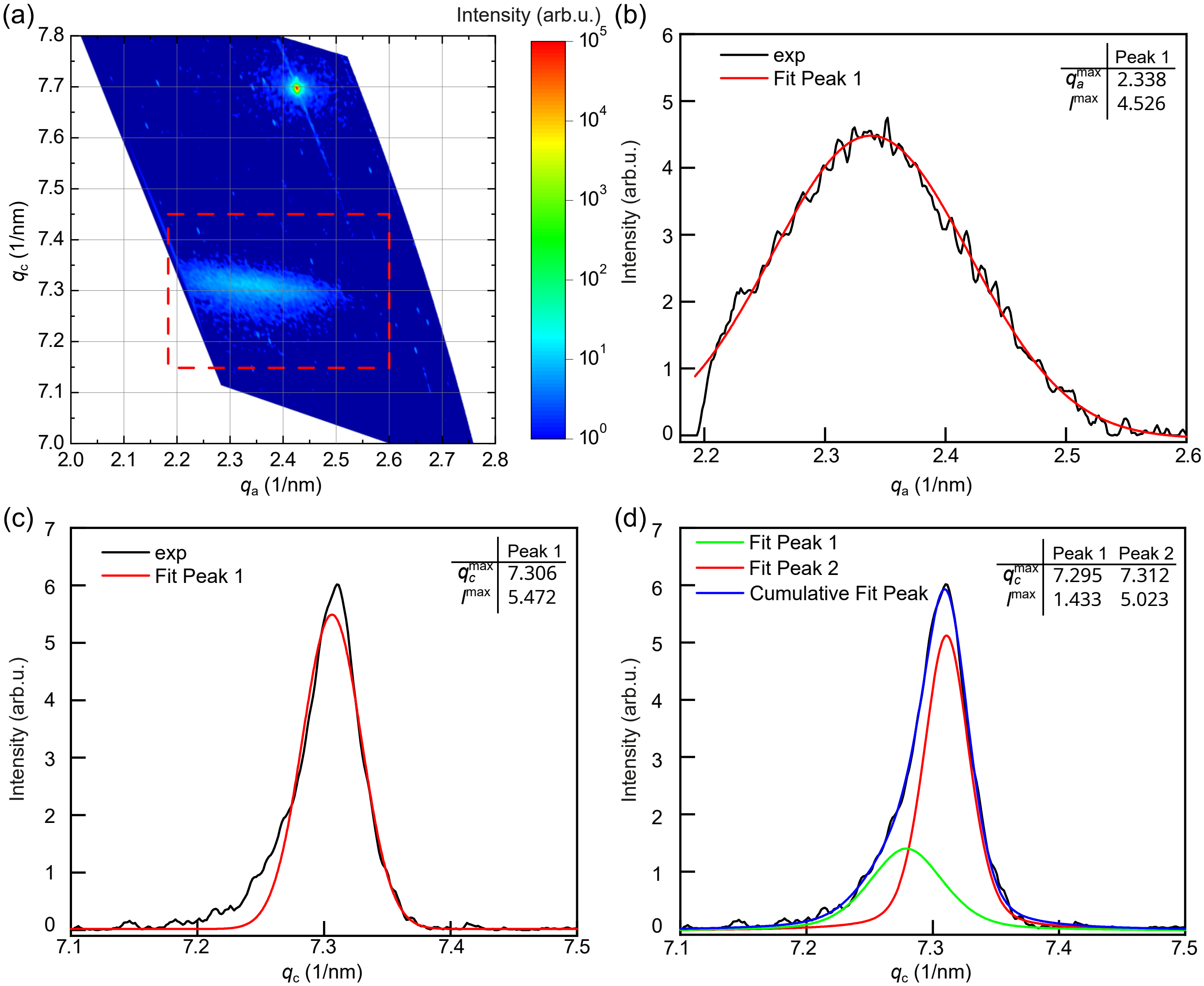

Supplementary Fig. 17. **Calculation of the average lattice parameters for 100-nm-thick Cr_2O_3 film.** (a) Experimental RSM data of the asymmetric $(1\,0\,\bar{1}\,10)$ reflection taken of thin film with the thickness of 100 nm. Line scans of the averaged intensity of the $(1\,0\,\bar{1}\,10)$ reflection along the q_a and q_c axes are shown in panels (b) and (c), respectively. The line scans are fitted using a single Gaussian function to extract the central position of the peak, which is taken as the average values of $q_{c,\mathrm{av}}$ and $q_{a,\mathrm{av}}$. The average value $q_{a,\mathrm{av}}$ is used to determine the average in-plane lattice parameter a_{av} (Fig. 1f of the main text). The average value $q_{c,\mathrm{av}}$ is used to determine the average out-of-plane lattice parameter c_{av} (Fig. 1g of the main text). In addition, the line scan along q_c (black curve) is fitted using a multipeak Gaussian function (S1) in panel (d). The best fit to the experimental data can be done with two Gaussian peaks, which are indicated with red and green curves in panel (d) and indicate the presence of two different zones in the thin film. The area, which is used for this analysis, is indicated with a red dashed box in panel (a). The fit results (peak position and intensity) are shown as a table in each panel.

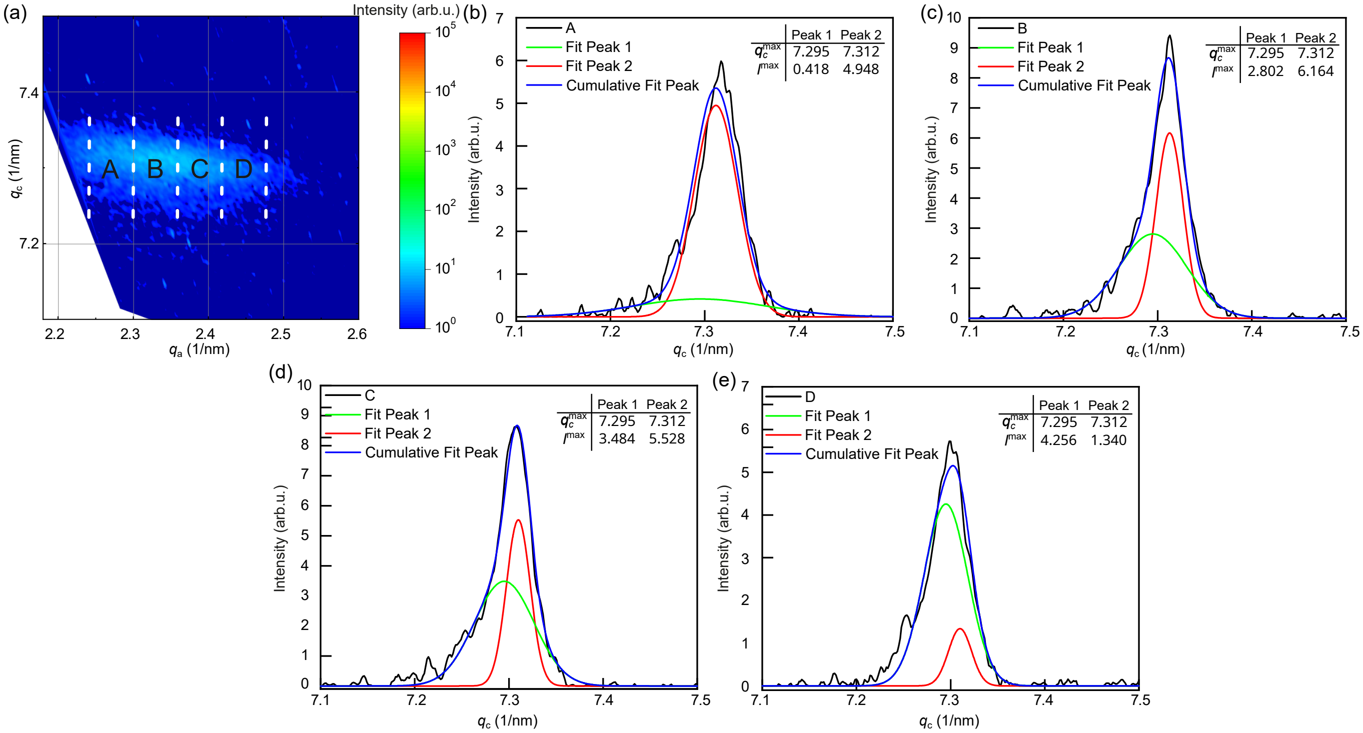

Supplementary Fig. 18. **Structural out-of-plane inhomogeneity of the 100-nm-thick Cr_2O_3 thin film.** (a) Part of the experimental RSM image of the asymmetric $(1\,0\,\bar{1}\,10)$ reflection. The area, which is used for this analysis, is indicated with a red dashed box in the Supplementary Fig. 17. The reflection is analyzed by slicing it along the $q_{c,\mathrm{av}}$ axis along the white dotted lines shown in panel (a). The interslice separation is $0.04\,\mathrm{nm}^{-1}$ along the $q_{c,\mathrm{av}}$ axis. Each slice is indicated with a capital letter. The set of line scans of the averaged intensity of the $(1\,0\,\bar{1}\,10)$ reflection along q_c axes is shown in panels (b-e). Each of the line scans (black curves) in panels (b-e) is fitted using a double peak Gaussian function with the fixed q_c values obtained in Supplementary Fig. 17d. The fit results (peak position and intensity) are shown as a table in each panel.

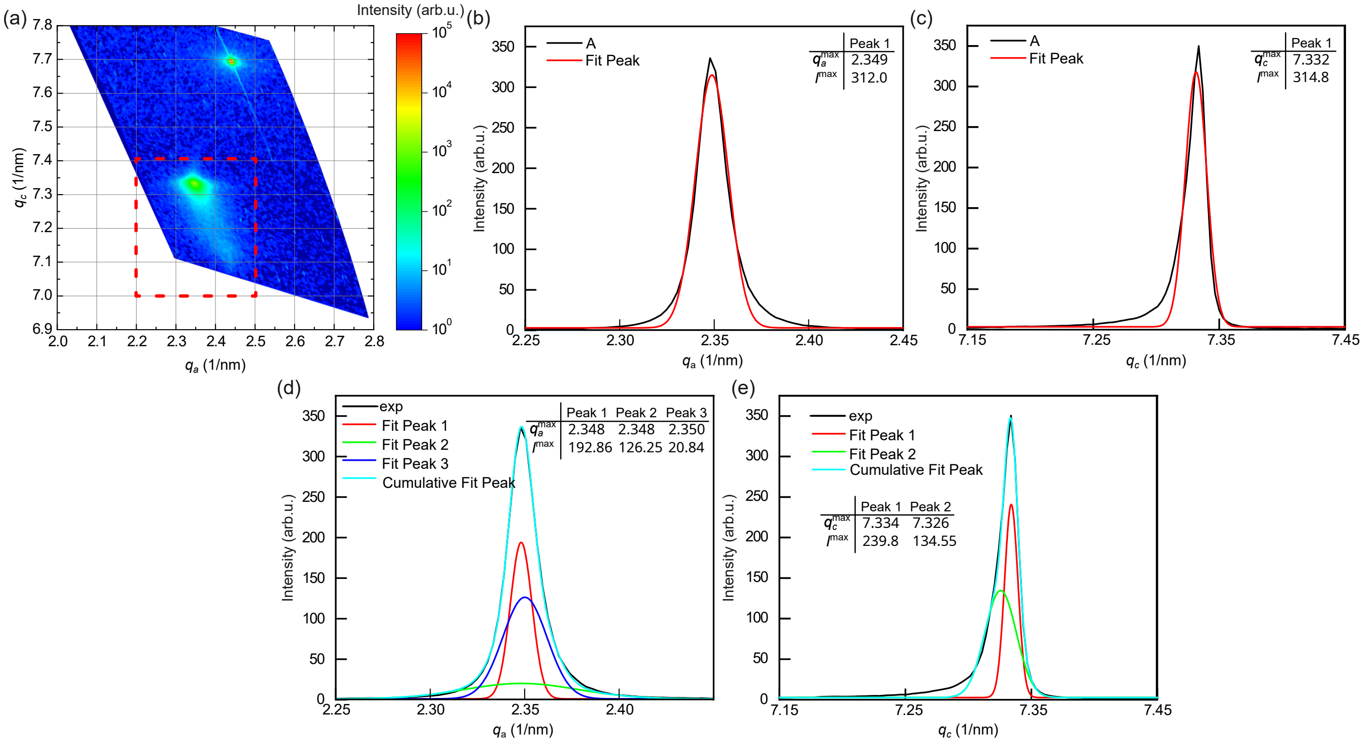

Supplementary Fig. 19. **Calculation of the average lattice parameters for 250-nm-thick Cr_2O_3 film.** (a) Experimental RSM data of the asymmetric $(1\,0\,\bar{1}\,10)$ reflection taken of thin film with the thickness of 250 nm. Line scans of the averaged intensity of the $(1\,0\,\bar{1}\,10)$ reflection along the q_a and q_c axes are shown in panels (b) and (c), respectively. The line scans are fitted using a single Gaussian function to extract the central position of the peak, which is taken as the average values of $q_{c,\mathrm{av}}$ and $q_{a,\mathrm{av}}$. The average value $q_{a,\mathrm{av}}$ is used to determine the average in-plane lattice parameter a_{av} (Fig. 1f of the main text). The average value $q_{c,\mathrm{av}}$ is used to determine the average out-of-plane lattice parameter c_{av} (Fig. 1g of the main text). Panels (d,e) show the line scans of the integral intensity of the $(1\,0\,\bar{1}\,10)$ reflection along the q_a and q_c axes fitted using multipeak Gauss (S1). The fit with triple-Gauss along q_a results in three peaks being at the same position. We use a fit along the q_c direction with double-Gauss resulting in two peaks, which are slightly separated. Considering the extent of the intensity distribution, the inhomogeneity affects a small portion of the film only, as the intensity outside the main peak is relatively low. The fit results (peak position and intensity) are shown as a table in each panel.

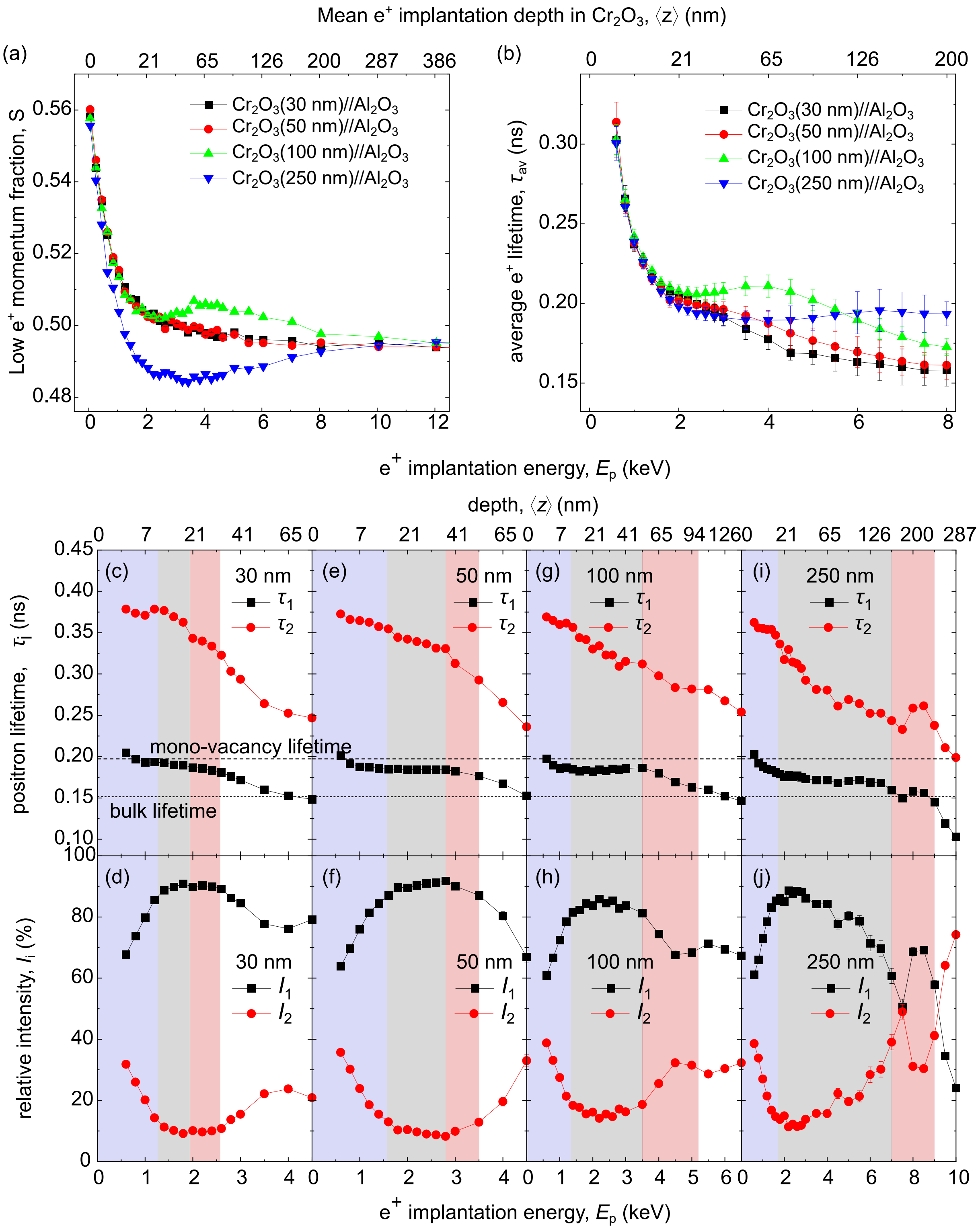

Supplementary Fig. 20. **Positron annihilation spectroscopy of Cr_2O_3 thin films.** (a) DB-VEPAS S-parameter as a function of positron implantation energy E_p and mean positron implantation depth $\langle z \rangle$ for Cr_2O_3 films of different thickness. (b) Average positron lifetime τ_{av} as a function of the implantation energy E_p and the mean positron implantation depth $\langle z \rangle$. (c–j) Depth profiles of positron lifetimes τ_1 and τ_2 and their relative intensities I_1 and I_2 for Cr_2O_3 films of different thickness. The sub-surface, top and interface regions of the film are marked with blue, grey and red background, respectively. The bulk and mono-vacancy positron lifetime are denoted as the dotted and dashed horizontal lines, respectively. Error bars correspond to the standard deviations of the fit.

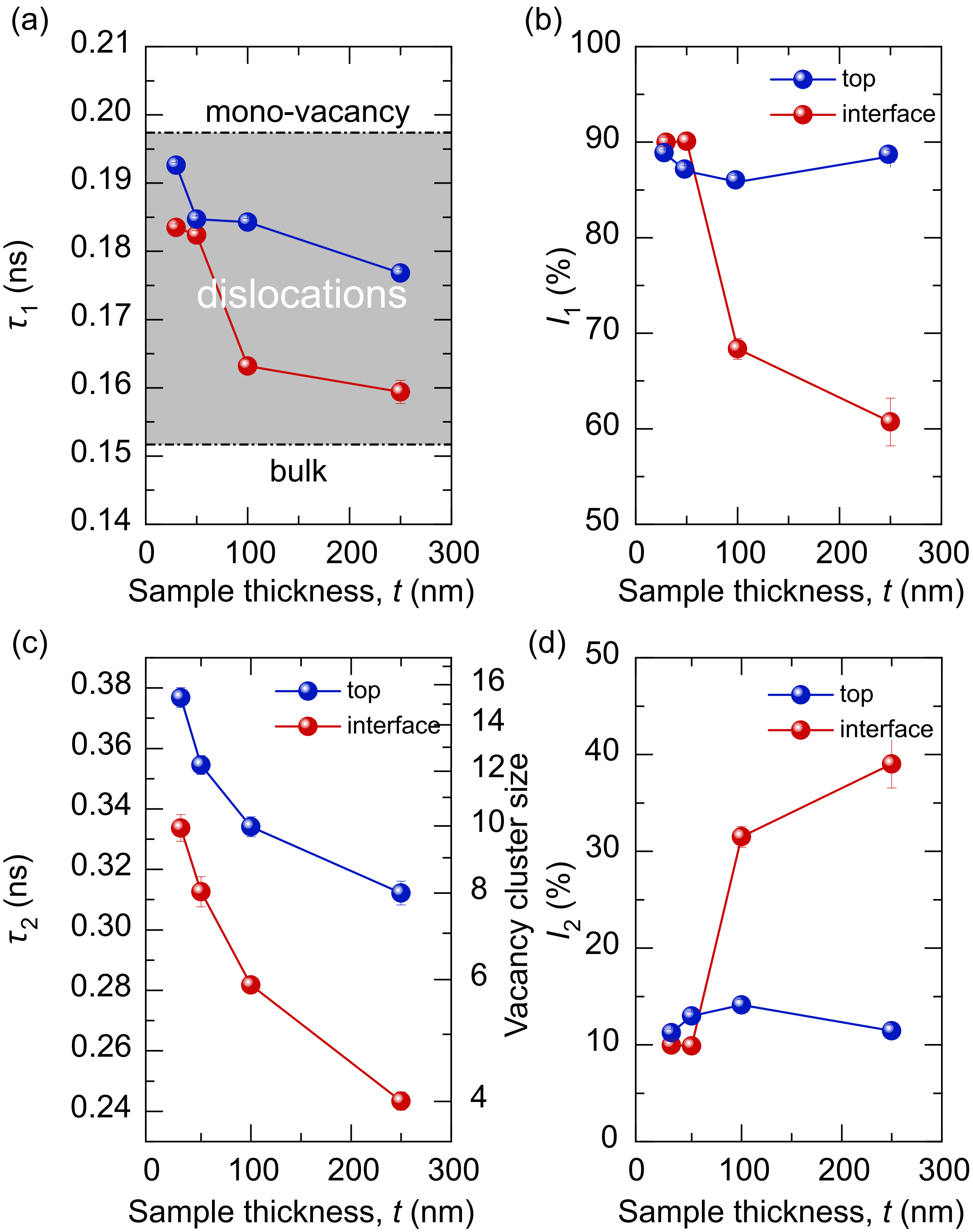

Supplementary Fig. 21. **Positron annihilation spectroscopy of Cr_2O_3 thin films.** Positron lifetime components (a) τ_1 and (c) τ_1 as well as their relative intensities (b) I_1 and (d) I_2 as a function of Cr_2O_3 films thickness estimated for the film top (blue spheres) and interface (red spheres) regions. In (a) a span of positron lifetime values corresponding to dislocations (gray area) is expected between the calculated value for annihilation at bulk and mono-vacancy states. Error bars correspond to the standard deviations of the fit.

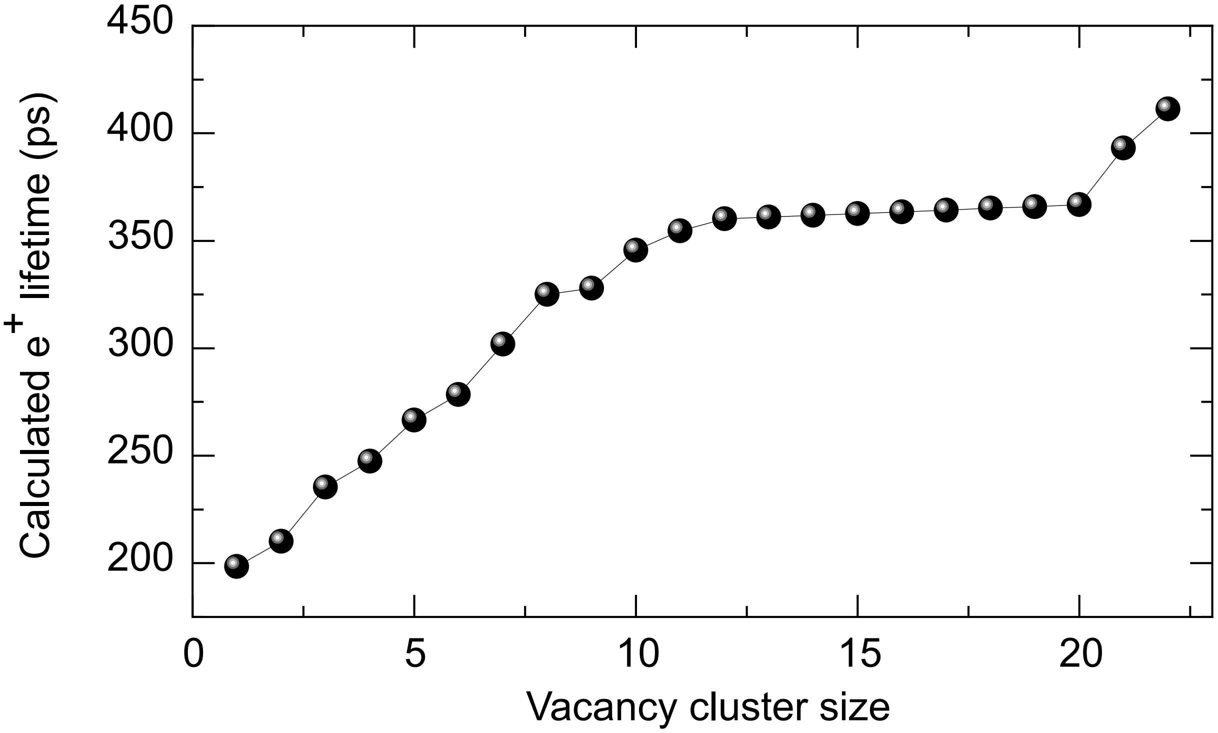

Supplementary Fig. 22. **ATSUP calculations of positron lifetimes as the function of the vacancy cluster size.**

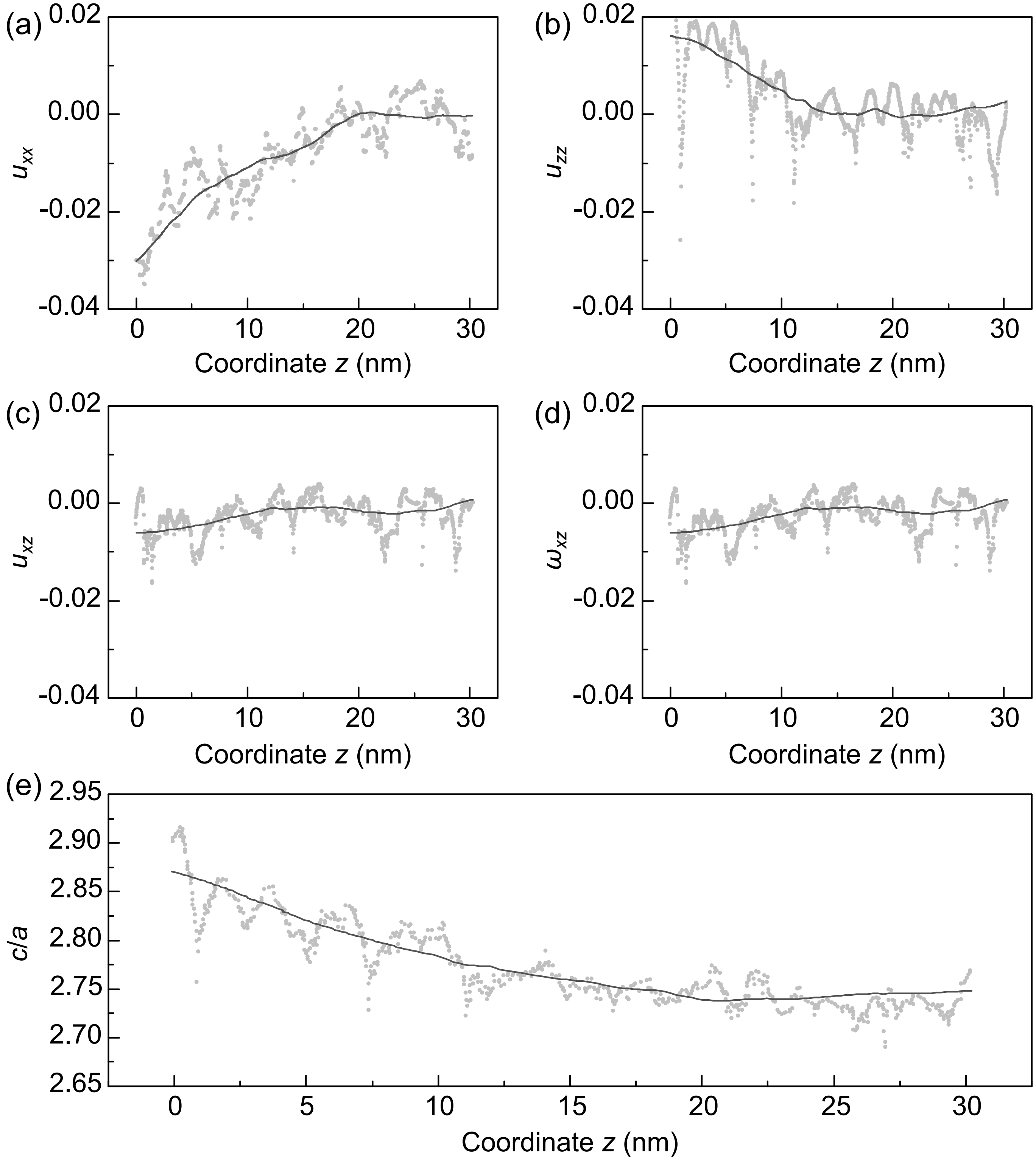

Supplementary Fig. 23. **High-resolution TEM analysis of the strain gradient of a 30-nm-thick Cr_2O_3 thin film.** Diagonal components of the strain tensor (a) u_{xx} and (b) u_{zz} reveal strain gradient from the bottom to the top film interface. (c) Shear component u_{xz} and (d) rotation component w_{xz} are almost constant along the film thickness. (e) Averaged profile of the ratio between the in-plane and out-of-plane lattice parameters (c/a) obtained from the analysis of HAADF-STEM image shown in Supplementary Fig. 5a.

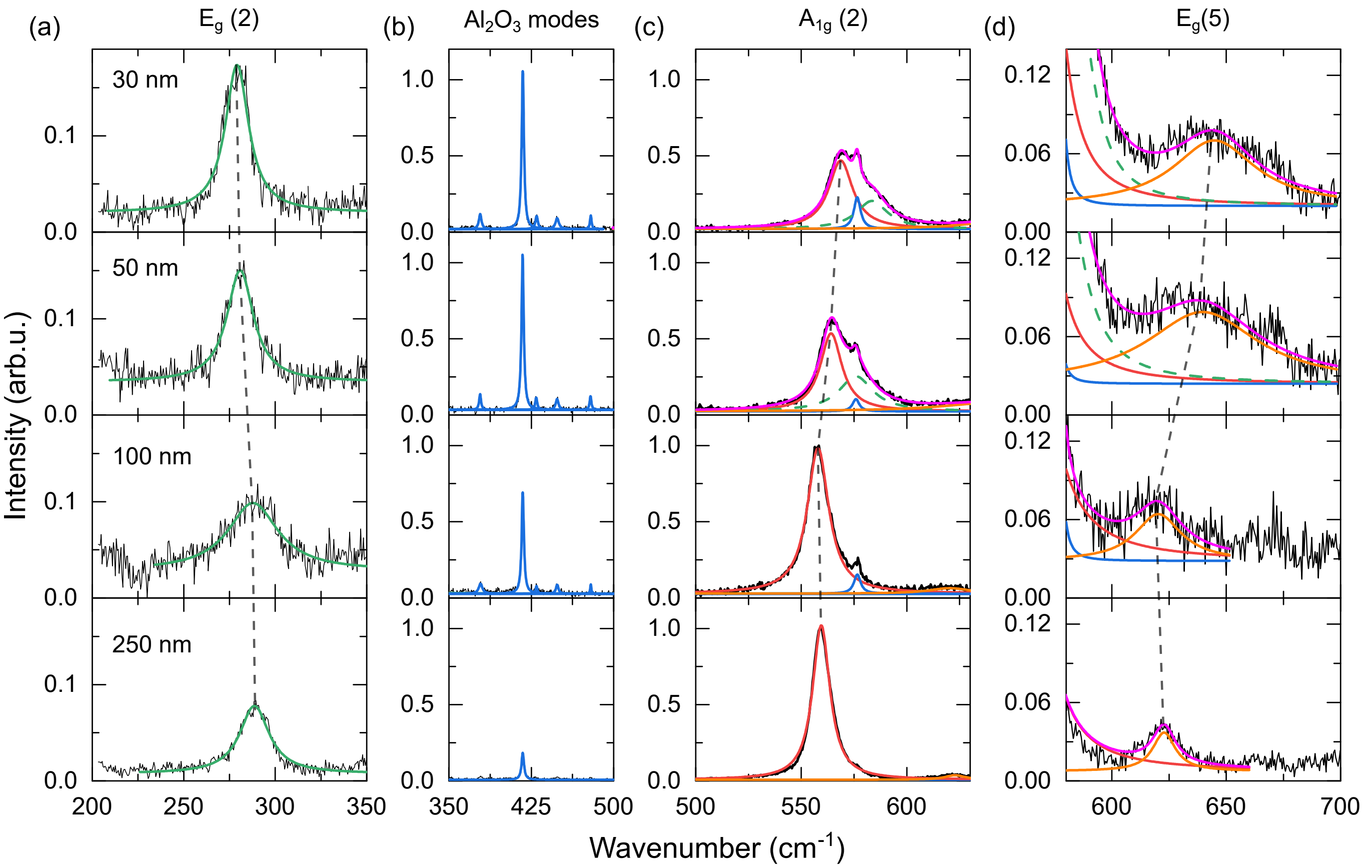

Supplementary Fig. 24. **Raman spectra of Cr_2O_3 thin films.** Raman spectra for the thin film samples with the thickness of 30 nm, 50 nm, 100 nm and 250 nm are shown in the rows from top to the bottom. Columns correspond to the different spectral ranges containing characteristic Raman modes. The black lines show the experimental data, the color lines are the Lorentzian fit of individual phonon modes. The magenta lines represent the total fitting curve for the spectral ranges where multiple peaks overlap. Dashed gray lines show the strain-induced shifts of the Cr_2O_3 phonons.

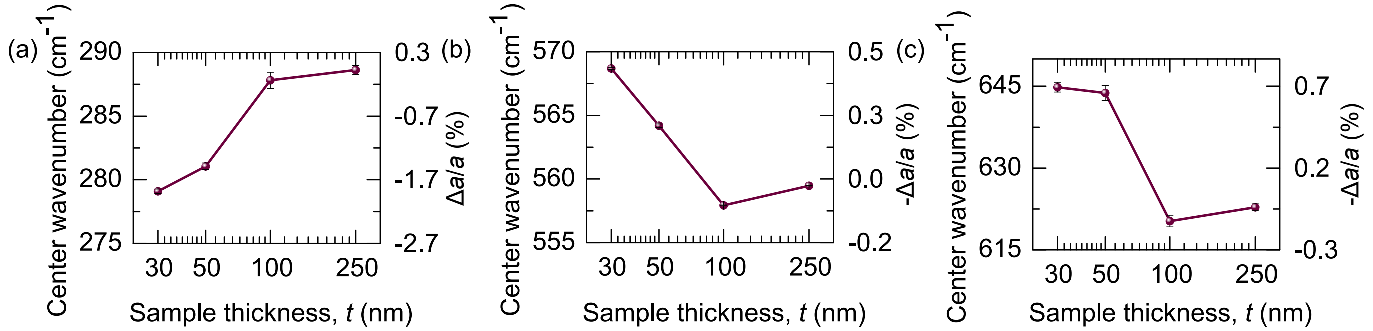

Supplementary Fig. 25. **Strain-induced shift of Raman-active phonons in Cr_2O_3 thin films.** Frequencies of $E_g(2)$, $A_{1g}(2)$ and $E_g(5)$ modes (left axis) and the estimated strain $\Delta a/a$ as a function of the film thickness. Note the inverted sign of the strain axis for the $A_{1g}(2)$ and $E_g(5)$ modes. Error bars for the center wavenumber represent the uncertainty of the peak position fitting of Raman spectra.

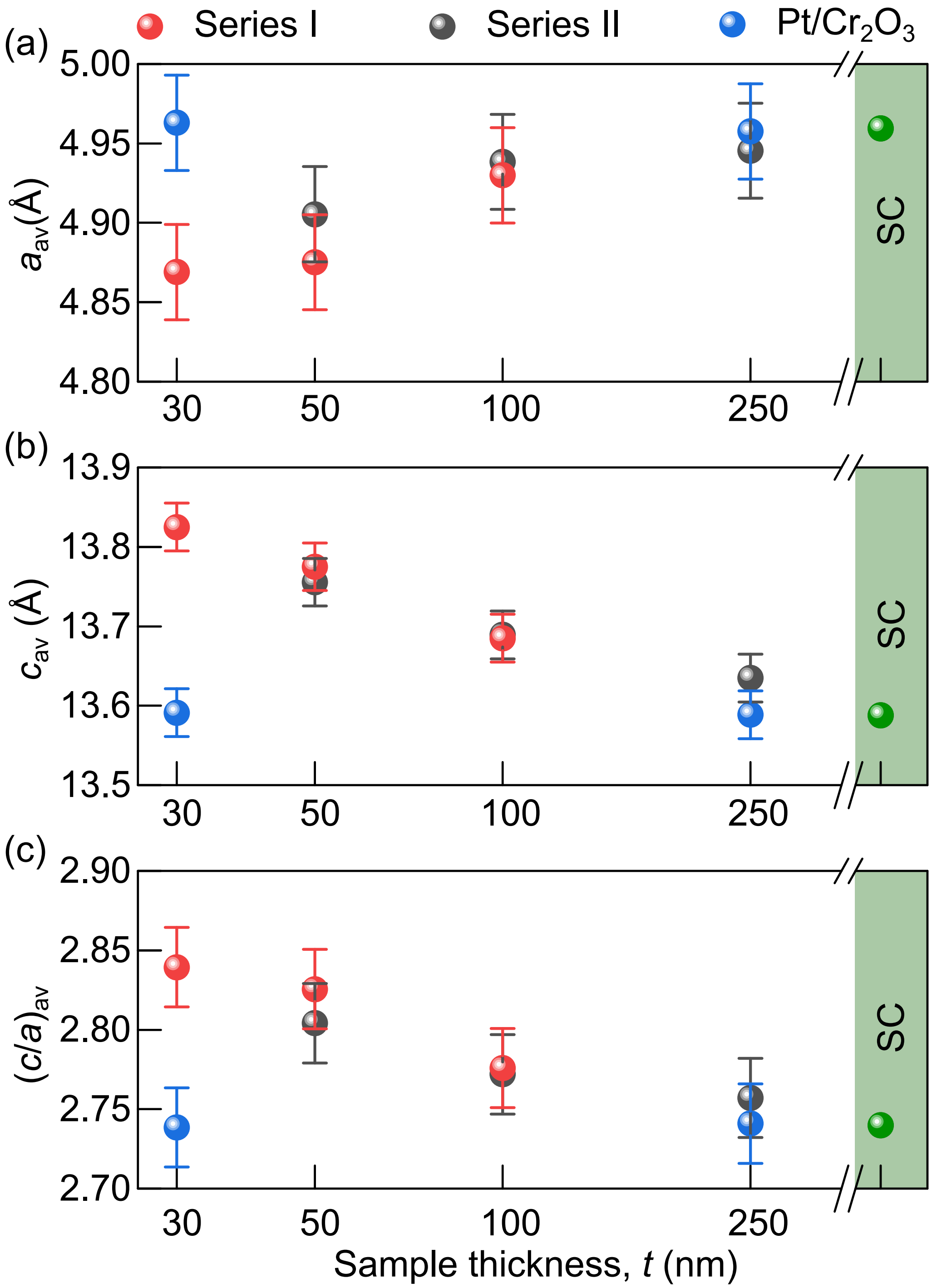

Supplementary Fig. 26. **Average lattice parameters of the Cr_2O_3 thin films.** Average values of the in-plane (a, panel (a)) and out-of-plane (c, panel (b)) lattice parameters and respective c/a ratio of the investigated Cr_2O_3 thin film samples calculated from two series of XRD investigations. Cr_2O_3 thin film samples prepared on Pt(20 nm) seed layer were investigated in the scope of Series II and indicated with blue symbols. "SC" stands for the lattice parameters of the reference Cr_2O_3 single crystal. Error bars of the lattice parameters are estimated from instrumental precision of the diffractometer and from the uncertainty of the peak fitting.

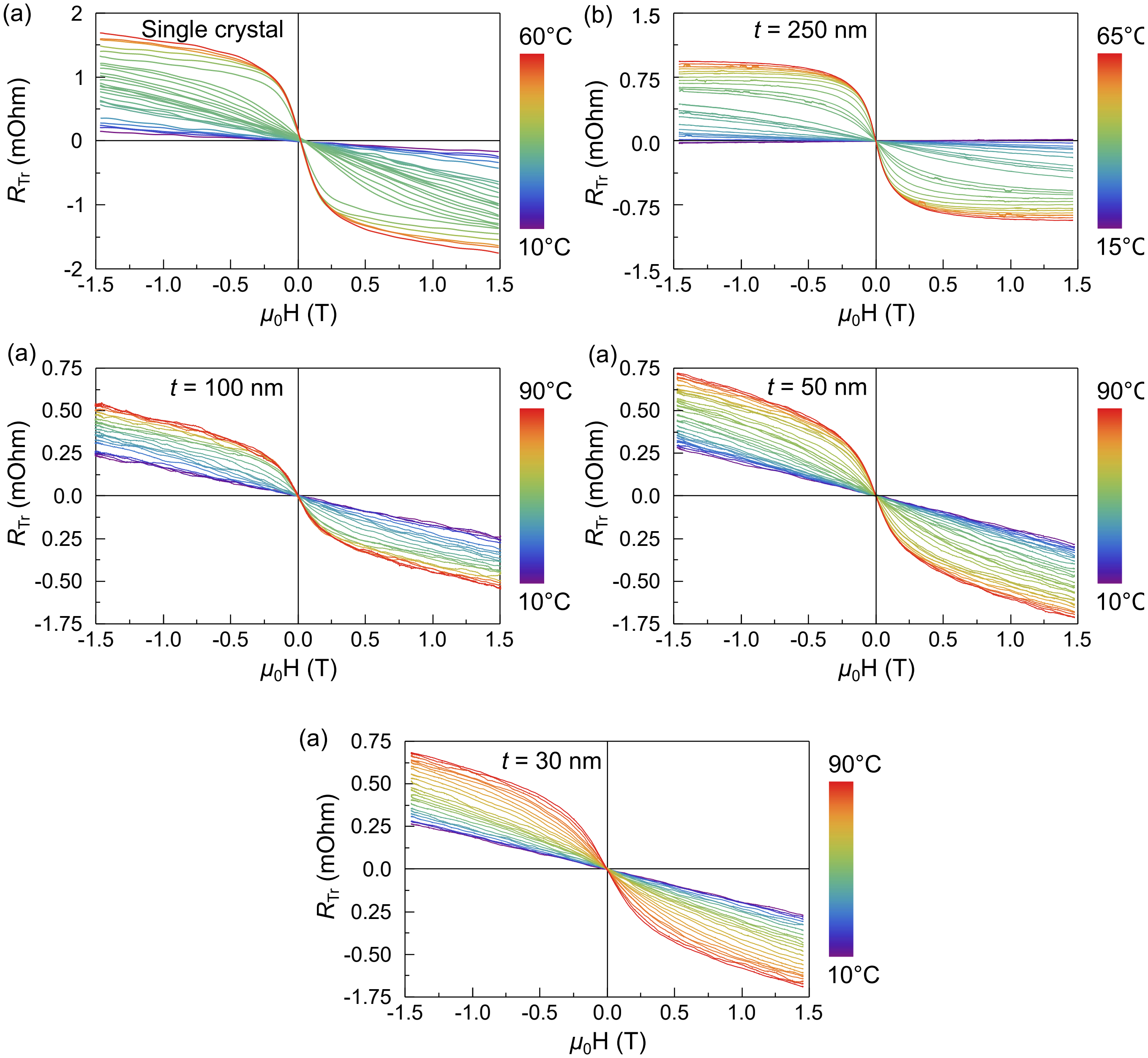

Supplementary Fig. 27. **Magnetotransport characterization of Cr_2O_3 samples.** Transversal resistance *vs* magnetic field $R_{Tr}(H)$ for (a) the reference Cr_2O_3 single crystal and thin films of different thickness: (b) 250 nm, (c) 100 nm, (d) 50 nm and (e) 30 nm. The curves are color coded with respect to the measurement temperature.

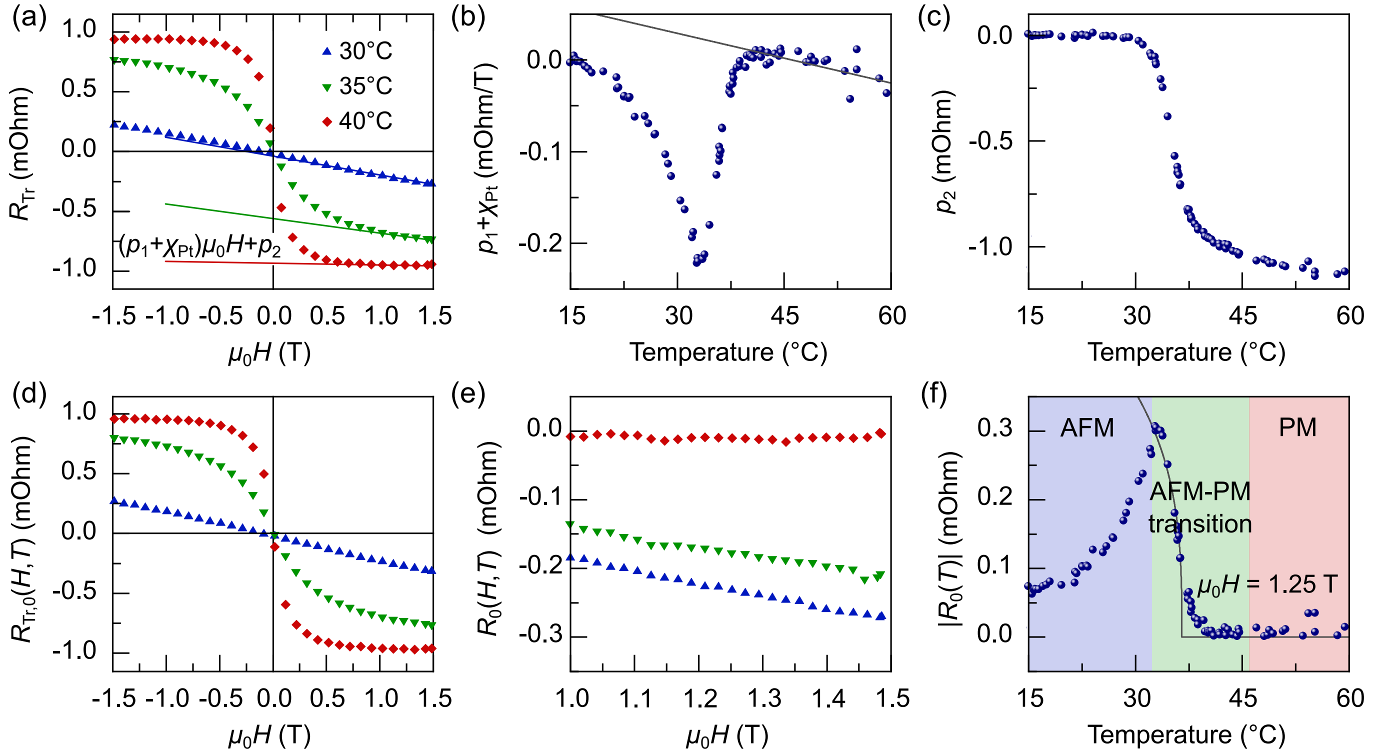

Supplementary Fig. 28. **Determination of the Néel temperature from magnetotransport measurements for a 250-nm-thick sample.** (a) $R_{\mathrm{Tr}}(H,T)$ sweeps at the selected temperatures for the 250-nm-thick Cr_2O_3 film. Symbols correspond to the measured data, and lines represent the linear fit at high fields ($\mu_0 H > 1\,\mathrm{T}$). (b,c) Temperature dependency of the fitting parameters p_1 and p_2 of the linear fit (S3) in the panel (a). (d) $R_{\mathrm{Tr},0}(H,T)$ sweeps with the subtracted normal Hall effect of Pt (see Eq. (S5)). (e) The part of the transversal resistance corresponding to the magnetism of Cr_2O_3, $R_0(H,T)$ (see Eq. (S6)). (f) Temperature dependency of absolute values of $R_0(T)$ at $\mu_0 H = 1.25\,\mathrm{T}$ (see Eq. (S7)). The color code of the background divides the plot into three segments with respect to antiferromagnet-paramagnet (AFM-PM) transition: the blue one indicates the purely antiferromagnetic state; the green background indicates the temperature region in the vicinity of the phase transition (this region is analyzed to assess the Néel temperature); the red background indicates the purely paramagnetic state.

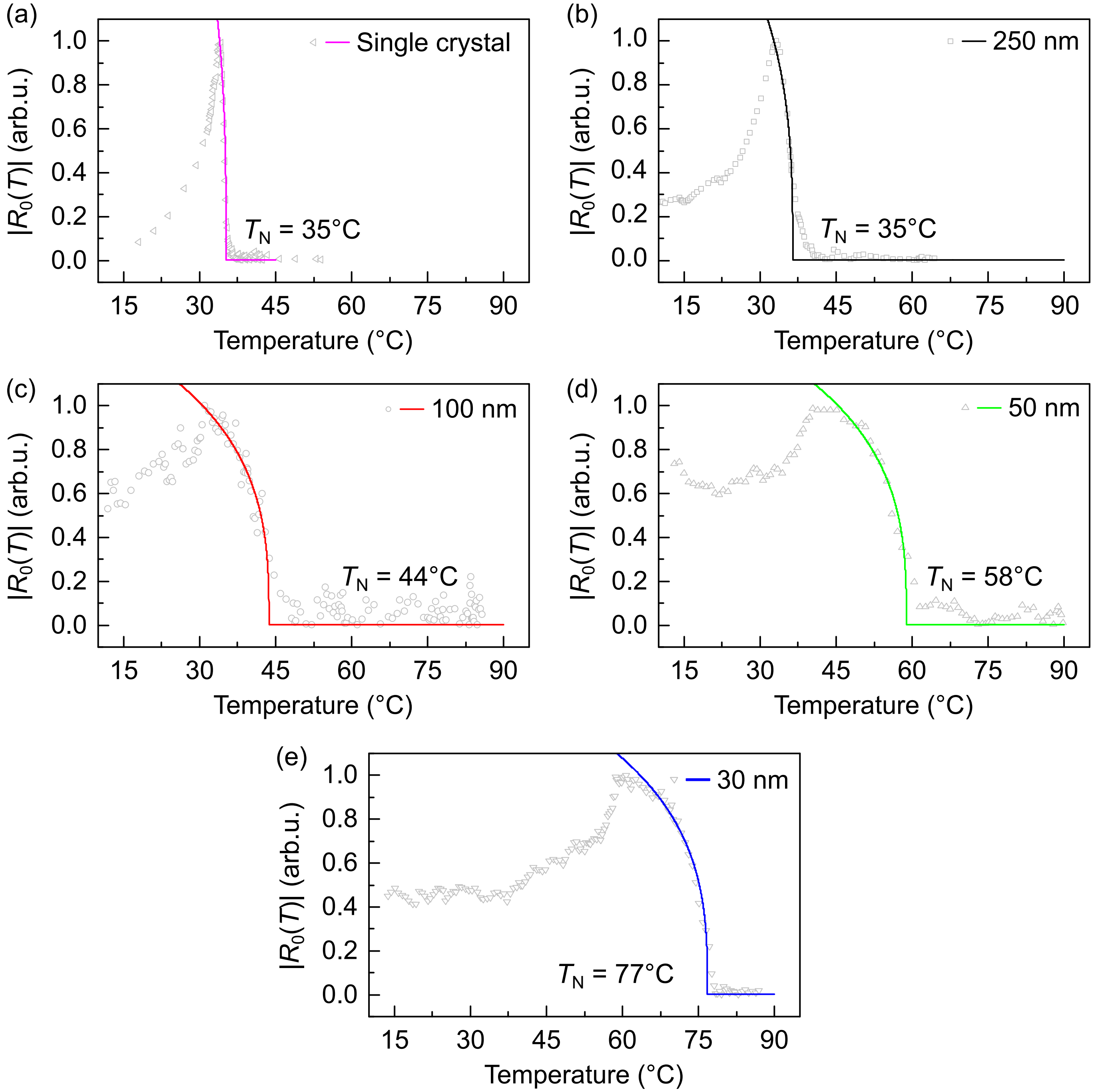

Supplementary Fig. 29. **The Néel temperature T_N^{top} at the top surface of the measured Cr_2O_3 samples.** Normalized variation of the absolute values of $R_0(T)$ with temperature at $\mu_0 H = 1.25\,T$ for the (a) reference Cr_2O_3 single crystal and thin film samples thickness of (b) 250 nm, (c) 100 nm, (d) 50 nm and (e) 30 nm. Symbols and lines correspond to the experimental data and fit (S4).

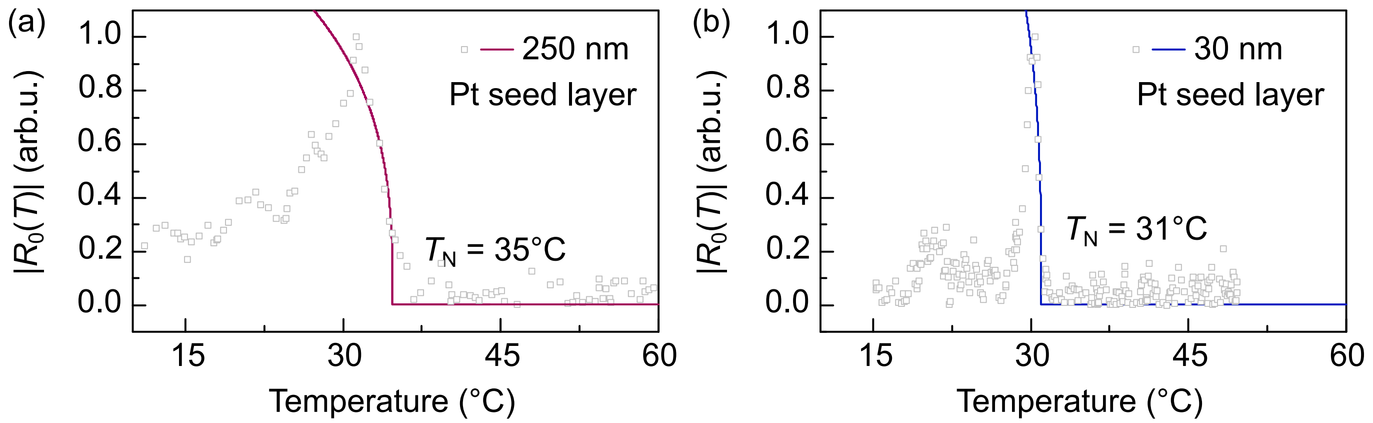

Supplementary Fig. 30. **The Néel temperature $T_{\mathrm{N}}^{\mathrm{top}}$ at the top surface of (a) 250-nm- and (b) 30-nm-thick Cr_2O_3 thin films grown on Al_2O_3 with a 20-nm-thick Pt seed layer.** Thin films of Cr_2O_3 grown on the Pt seed layer are relaxed due to their non-epitaxial growth. This results in values of the Néel temperature being close to those measured on single crystal, independently of the Cr_2O_3 thickness.

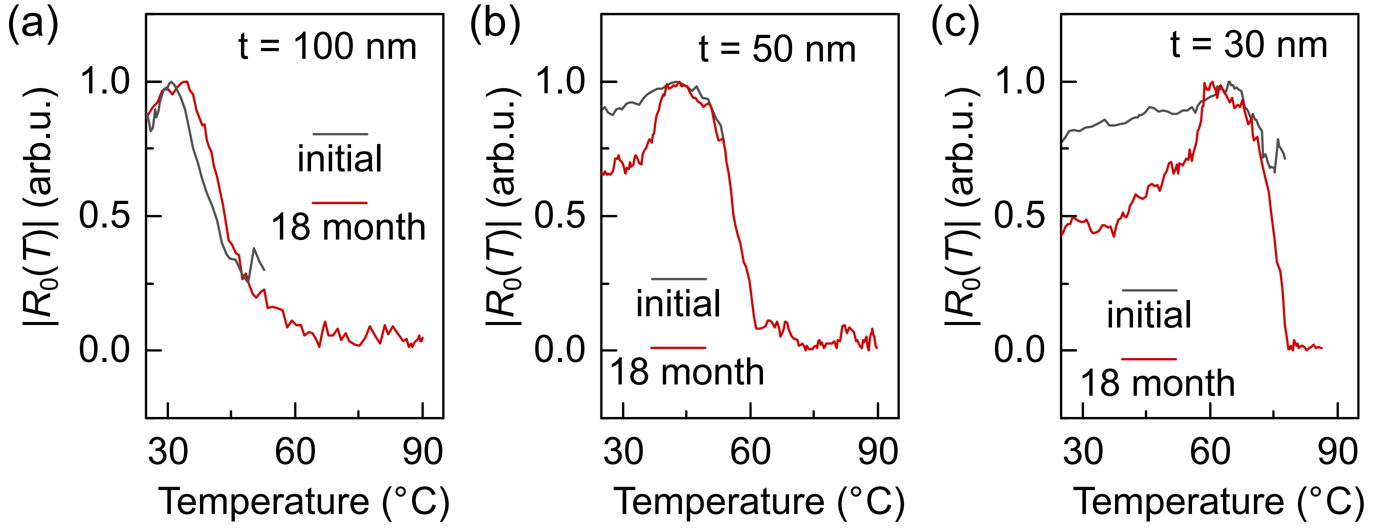

Supplementary Fig. 31. **Stability over time of the top surface Néel temperature $T_{\mathrm{N}}^{\mathrm{top}}$ of Cr_2O_3 thin films**. Temperature evolution of the absolute valeus of $R_0(T)$ dependence at $\mu_0 H = 1.25\,\mathrm{T}$ taken right after deposition and after 18 months.

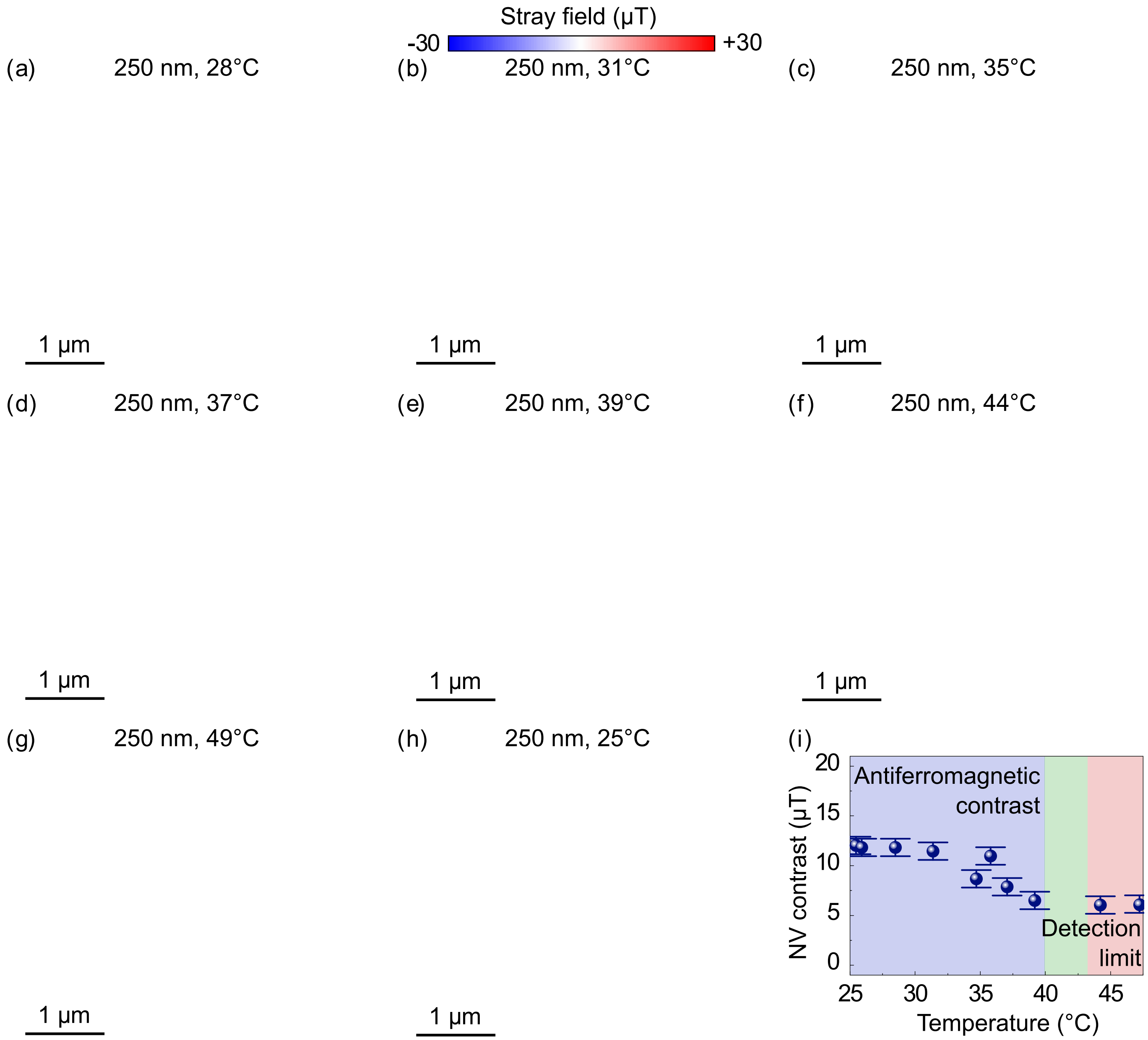

a

Supplementary Fig. 32. **NV magnetometry of 250-nm-thick Cr_2O_3 thin film.** (a-g) A series of NV magnetometry images represents the evolution of the domains contrast in Cr_2O_3 with increasing temperature. The domain contrast decreases as temperature is rising and vanishes at approximately 40 °C. (h) The domain pattern taken at the same spot after cooling the sample down to 25 °C after it was warmed to 50 °C. The qualitative difference in the morphology of the domain pattern observed in panel (a) and (h) indicates that heating the sample to 50 °C was sufficient to bring it to the paramagnetic state. (i) Magnetic stray field contrast calculated from the NV images. The background is color coded according to three temperature regions: blue: antiferromagnetic state, green: vicinity of the phase transition, red: minimum detectable signal due to thermal fluctuations. Error bars correspond to the haldwidth of the distribution.

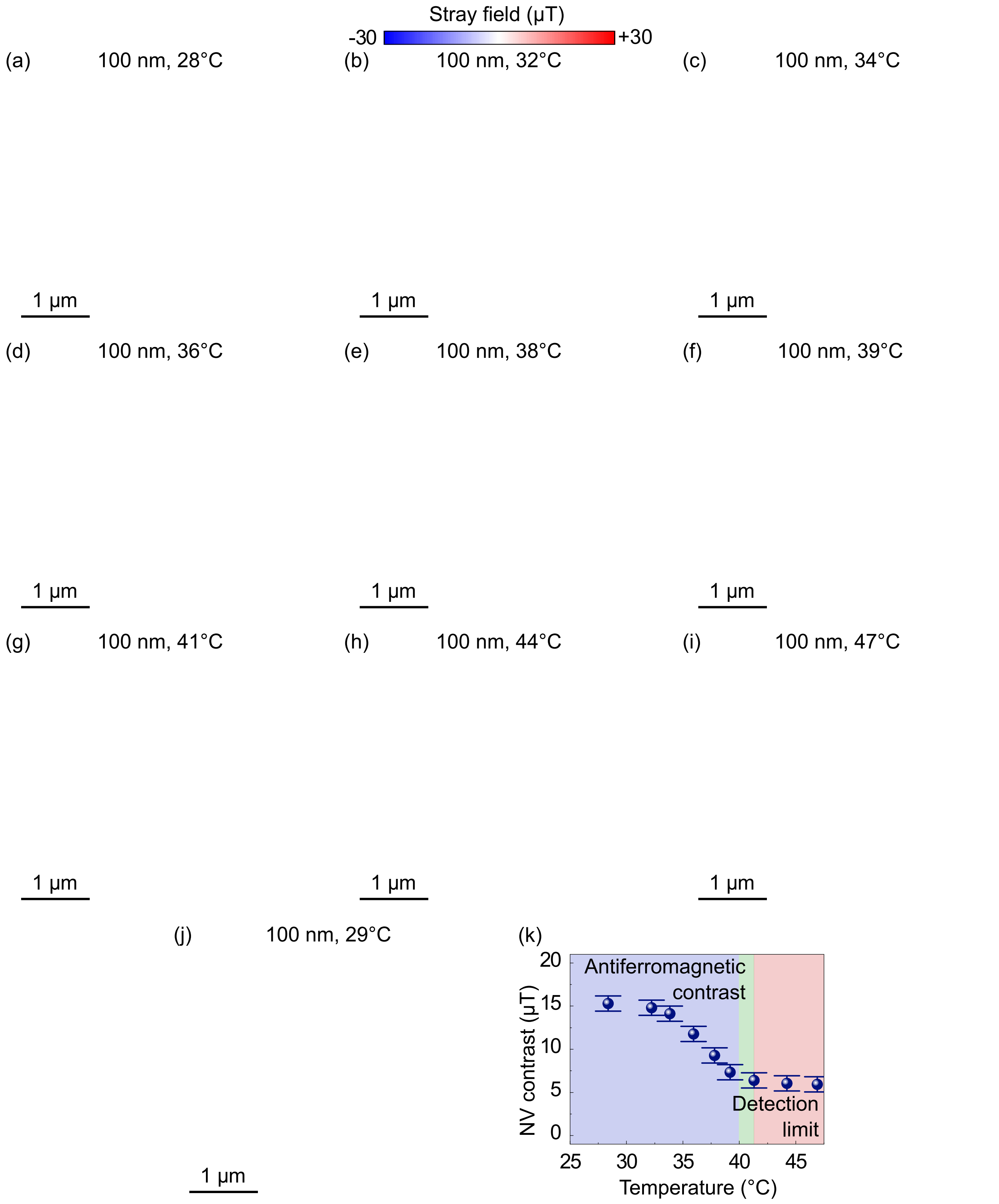

Supplementary Fig. 33. **NV magnetometry of 100-nm-thick Cr_2O_3 thin film.** (a-i) A series of NV magnetometry images represents the evolution of domain contrast in Cr_2O_3 with temperature. The domain contrast decreases as temperature is rising and vanished upon reaching 41°C. (j) The domain pattern taken at the same spot after cooling the sample down to 29 °C after it was warmed to 47 °C. The qualitative difference in the morphology of the domain pattern observed in panel (a) and (j) indicates that heating the sample to 47 °C was sufficient to bring it to the paramagnetic state. (k) Magnetic stray field contrast calculated from the NV images. The background is color coded according to three temperature regions: blue: antiferromagnetic state, green: vicinity of the phase transition, red: minimum detectable signal due to thermal fluctuations. Error bars correspond to the haldwidth of the distribution.

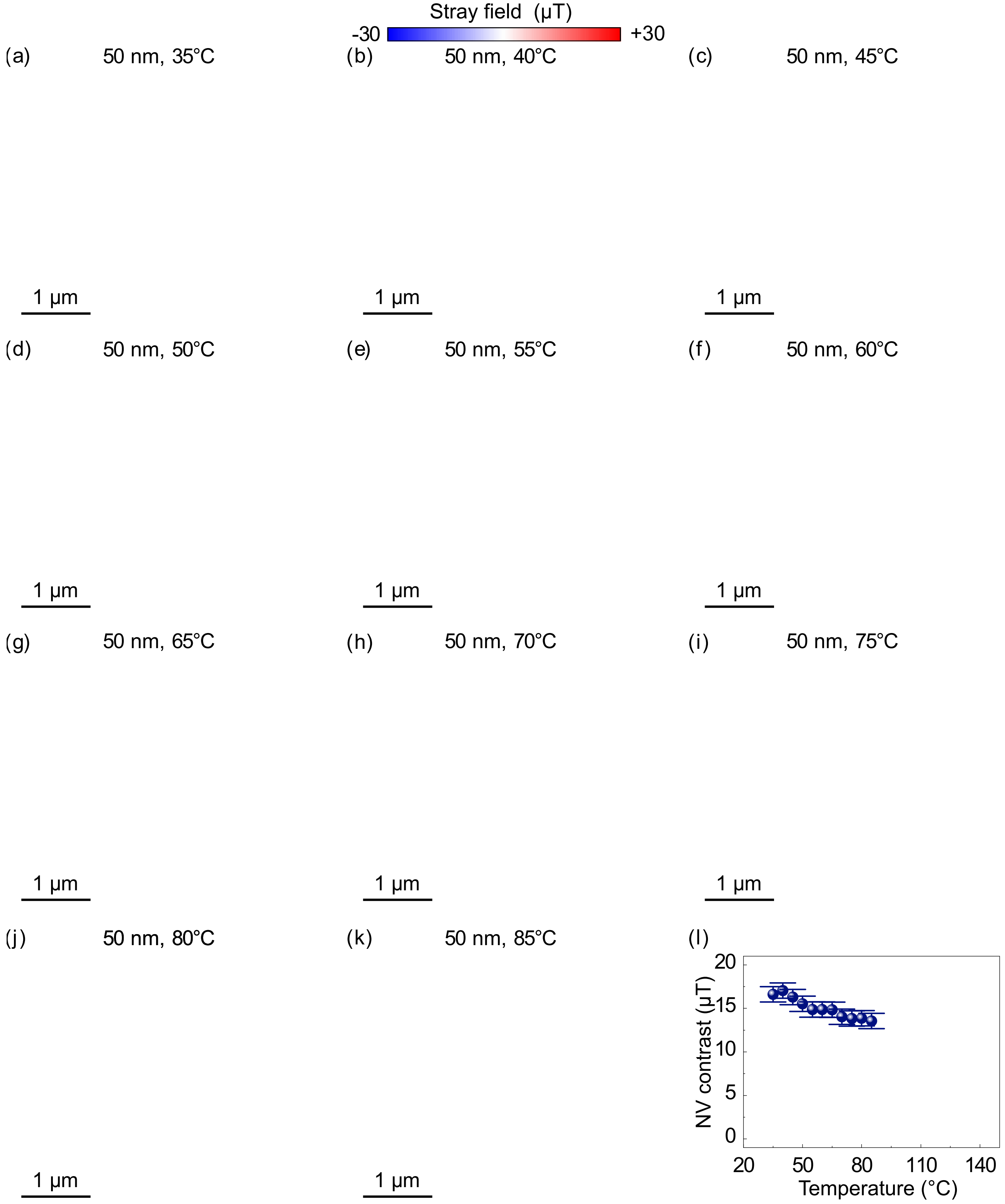

Supplementary Fig. 34. **NV magnetometry of 50-nm-thick Cr_2O_3 thin film.** (a-k) A series of images represents the evolution of domain contrast in Cr_2O_3 with temperature. The domain contrast decreases as temperature is rising and retains at temperatures higher than 57°C, which is determined as T_N^{top} from magnetotransport measurements. The domain contrast retains upon reaching temperature of 85°C, which is the maximum achievable in the setup. (l) Magnetic stray field contrast calculated from the NV images. Error bars correspond to the haldwidth of the distribution.

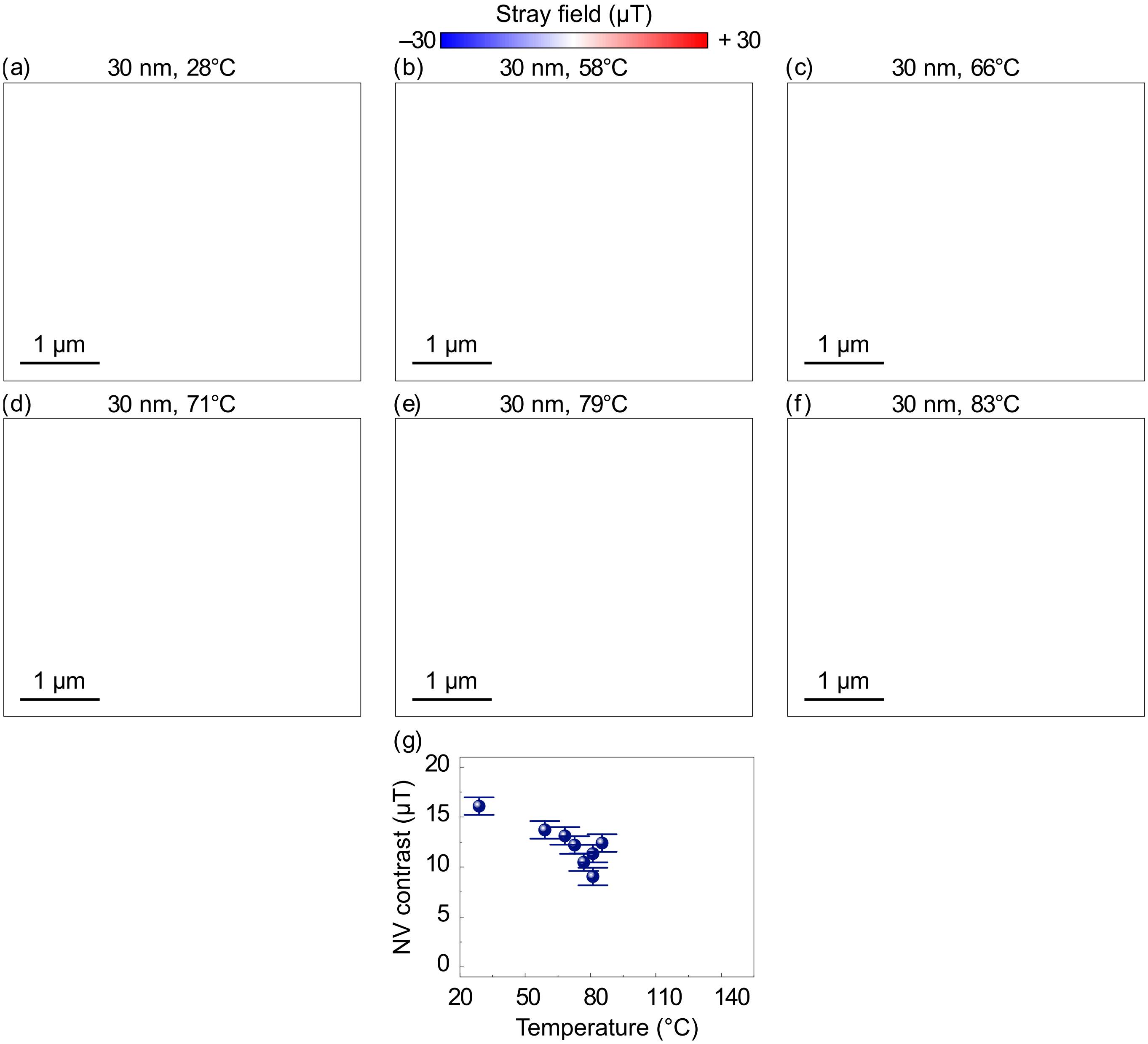

Supplementary Fig. 35. **NV magnetometry of 30-nm-thick Cr_2O_3 thin film.** (a-f) A series of images represents the evolution of domains contrast in Cr_2O_3 with increasing temperature. The domain contrast retains upon reaching the maximum achievable temperature of 83°C. (g) Magnetic stray field contrast calculated from the NV images. Error bars correspond to the haldwidth of the distribution.

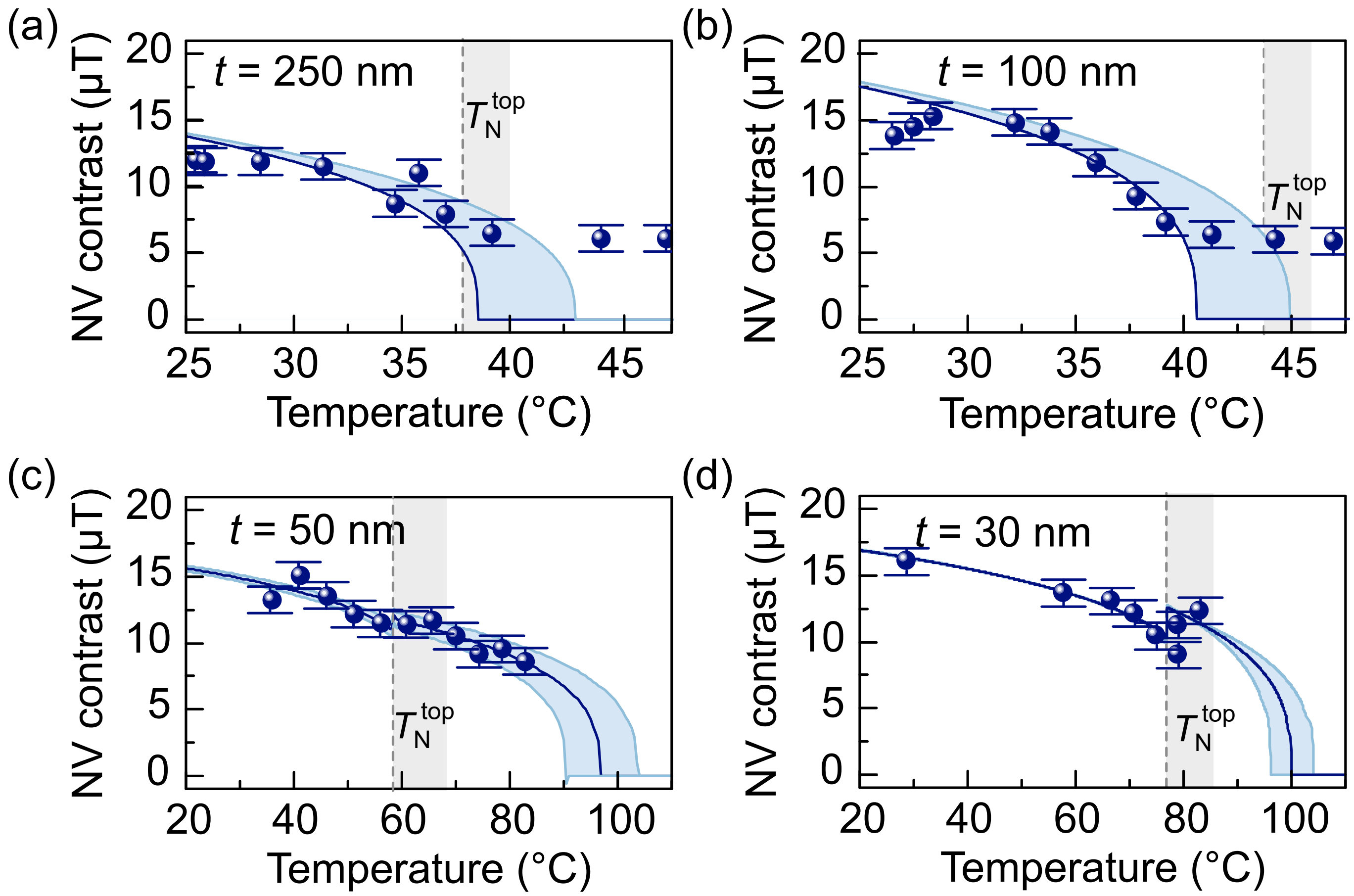

Supplementary Fig. 36. **Evolution of contrast of antiferromagnetic domains with temperature for films of different thickness obtained by means of NV magnetometry.** Blue symbols correspond to the antiferromangetic domain contrast obtained from the NV measurements. Blue line is the fit accordingly to the equation (4, main text) and shaded blue areas are uncertainty estimations for the fit. Vertical dashed gray line shows $T_{\mathrm{N}}^{\mathrm{top}}$ and the regions shaded in gray show uncertainty in the determination of $T_{\mathrm{N}}^{\mathrm{top}}$. Error bars correspond to the haldwidth of the distribution.

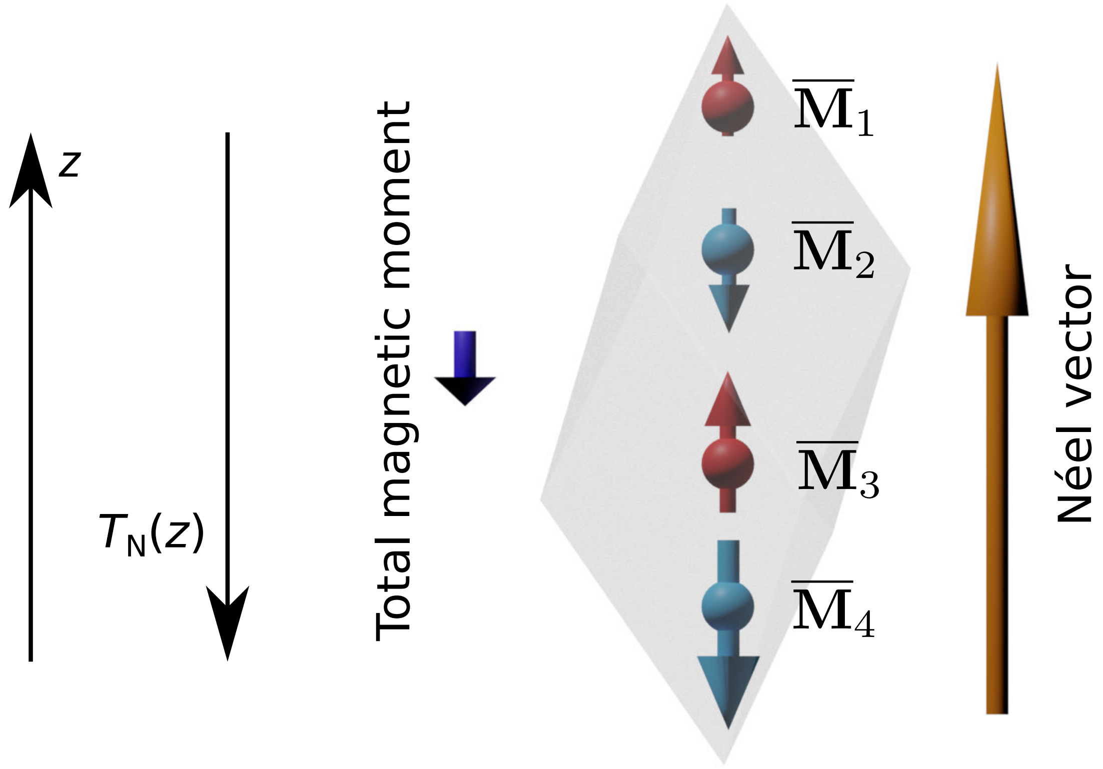

Supplementary Fig. 37. **Ferromagnetism, induced by the inhomogeneous thermal reduction of the sublattice magnetization in Cr_2O_3.** The schematic shows a rhombohedral primitive cell of Cr_2O_3. The four arrows labeled as $\overline{\mathbf{M}}_{1...4}$ represent sublattice magnetizations at the respective vertical positions. Two antiferromagnetic sublattices are highlighted by the arrows of red and light-blue color. The gradient of the Néel temperature $T_N(z)$ along the cell axis at the given sample's temperature T leads to the reduction of the magnetization at the given z slice according to the value of $T/T_N(z)$. This mechanism does not change the direction of the Néel vector (orange arrow) but leads to the emergence of the magnetic moment (blue arrow) in the direction of the gradient of T_N.

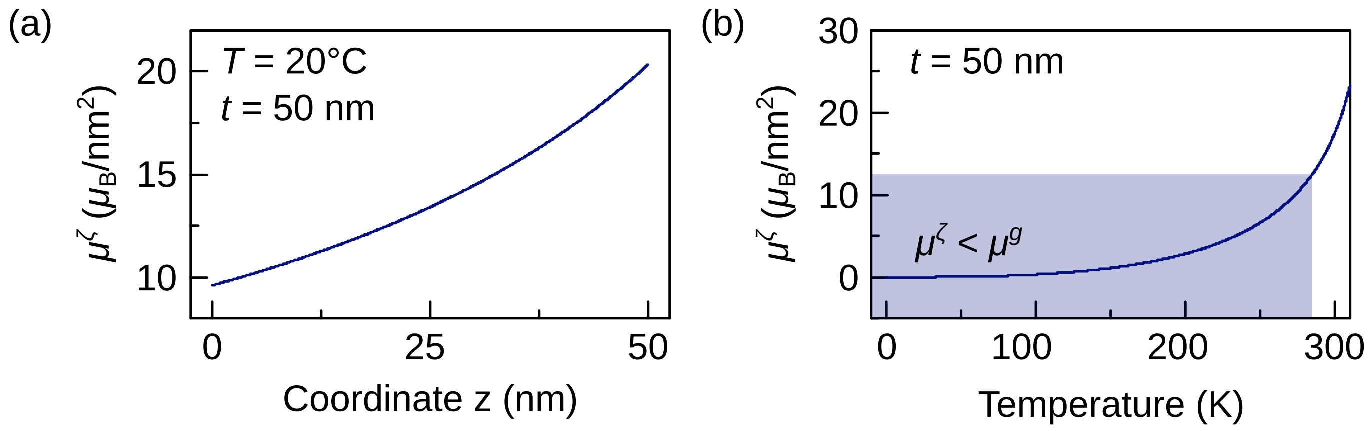

Supplementary Fig. 38. **Flexomagnetic coefficient of thermal origin** $\mu^\zeta = \langle \mu^\zeta_{ijkl} \rangle$**.** (a) Distribution of μ^ζ along film thickness at $T = 20°$ C. (b) The dependence $0.5[\mu^\zeta(z=0) + \mu^\zeta(z=t)]$ as a function of temperature. μ^ζ becomes smaller than μ^g at $T = 283\,$K (highlighted with blue shaded region).

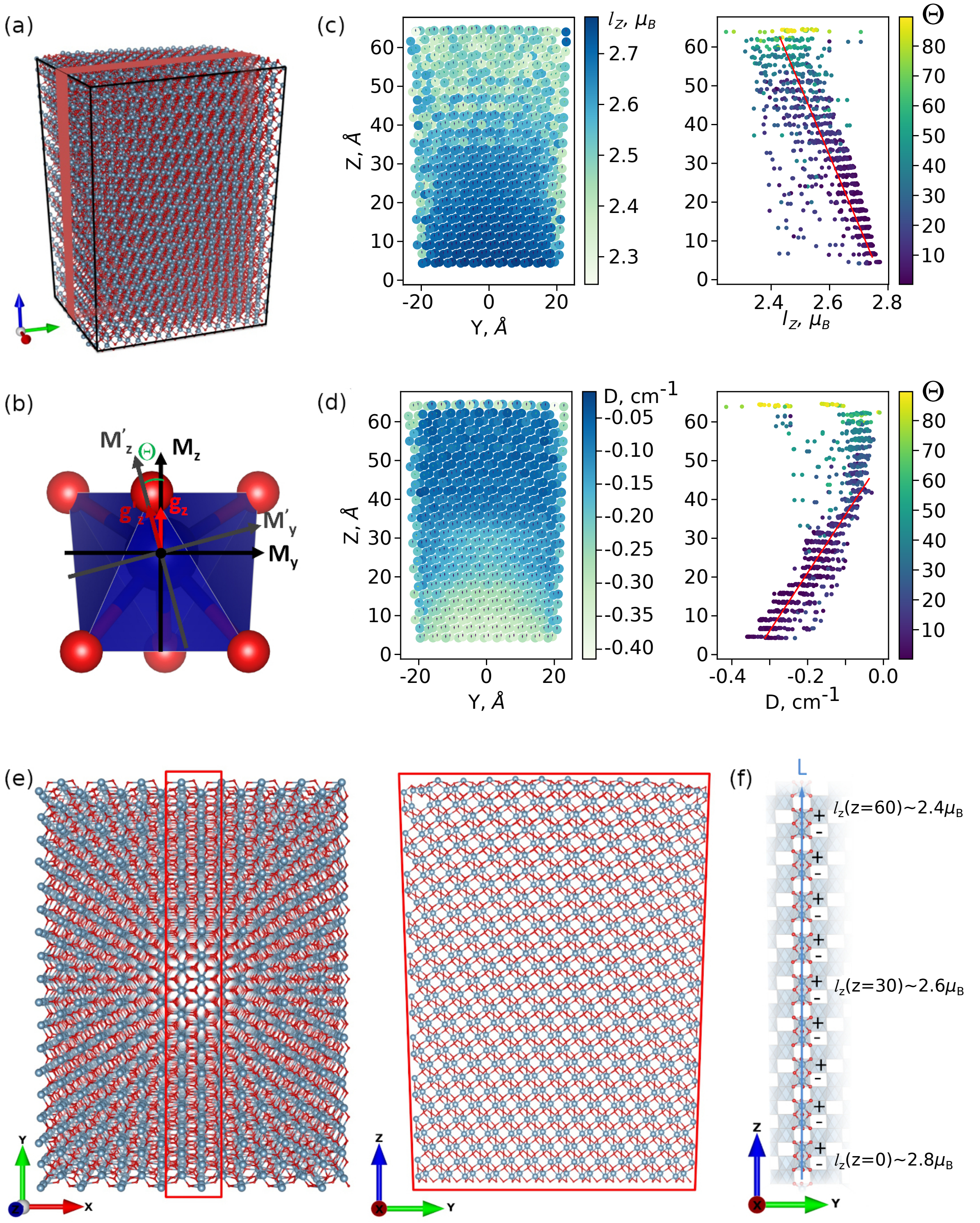

Supplementary Fig. 39. **Multiscale modelling of corundum-type lattice.** (a) Well-equilibrated Cr_2O_3 corundum structure with 40Å×40Å×60Å block size and an indication of the middle point along x-axis for yz-plane cross-section cut (the region where the local anisotropy of every Cr^{3+} was analyzed). (b) Example of $[CrO_6]$ polyhedral geometry with superposed magnetic frame transformation ($M_z \to M'_z$ makes angle Θ) and g_z projections. (c) Colorized scatter plot with l_z values attached to Cr^{3+} site positions in yz-cut and the distribution plot of l_z values as a function of z with a color map of the magnetic frame rotation (angle Θ). (d) Colorized scatter plot with D values attached to Cr^{3+} site positions and the distribution plot as a function of z with a color map of the magnetic frame rotation (angle Θ). (e) Different projections of the systems. (f) A possible orientation of the order parameter $\mathbf{L}$ with AFM $\{+-+-\}$ moment alteration but reduced on-site moment strength as a function of z.

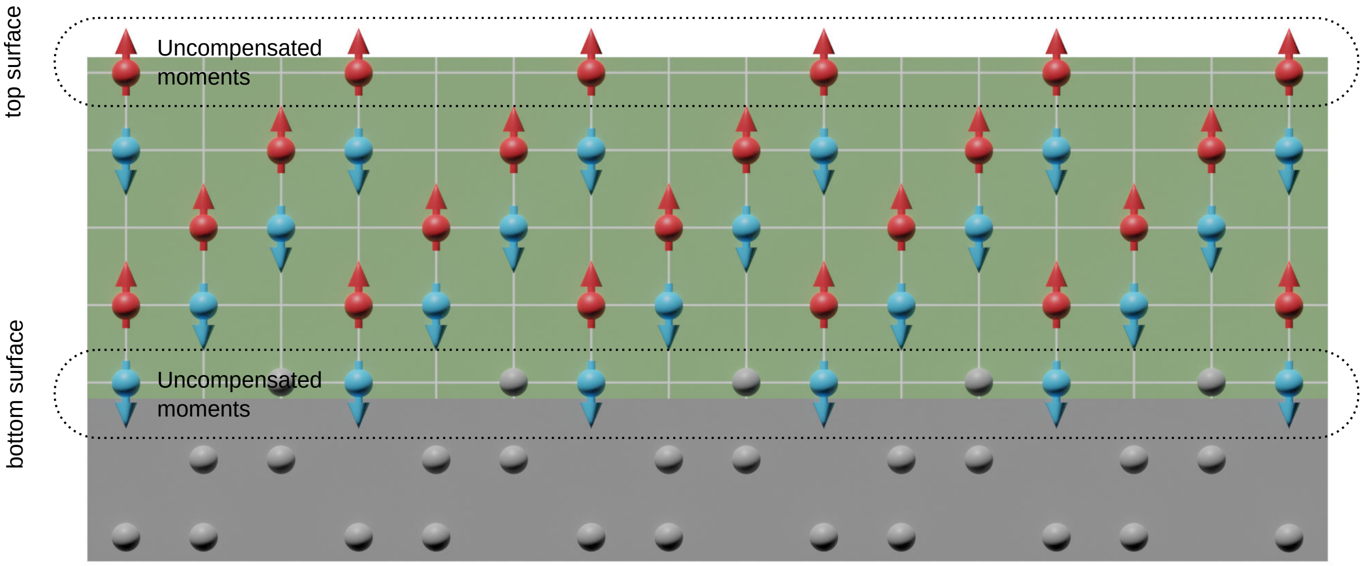

Supplementary Fig. 40. **Uncompensated magnetization on the top and bottom interfaces.** The crystallographic cut (0 0 0 1) possesses an uncompensated magnetization of the areal density $\sigma_{\mathrm{top,bot}} \sim 2.2\,\mu_{\mathrm{B}}\,\mathrm{nm}^{-2}$ at room temperature. The presence of domain walls allows detection of stray fields using NV magnetometry. The green and gray parts of the sample correspond to the Cr_2O_3 and Al_2O_3, respectively.

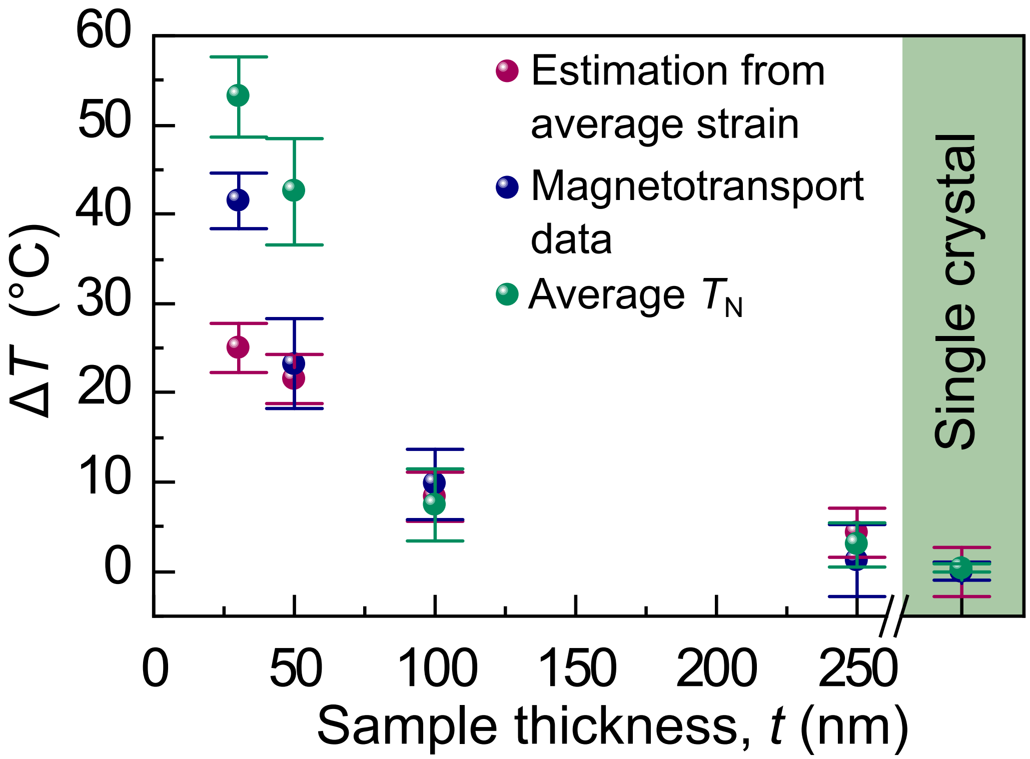

Supplementary Fig. 41. **Effective piezomagnetic contribution $\Delta T_{\mathbf{piezo}}$ to the Néel temperature.** The purple symbols show the enhancement of the Néel temperature T_N due to the homogeneous strain corresponding to the piezoeffect (Eq. (S19), see also [50]), where the average strain value for the film of the given thickness is used. Blue symbols show the Néel temperature T_N^{top} at the top film surface accessed by the magnetotransport. Green symbols show mean value $(T_N^{top} + T_N^{bot})/2$ of the Néel temperature for the given thin film sample. Both curves behave qualitatively in the same way and are close by value. Error bars correspond to uncertaninity of the fit of the magnetotransport and NV data.

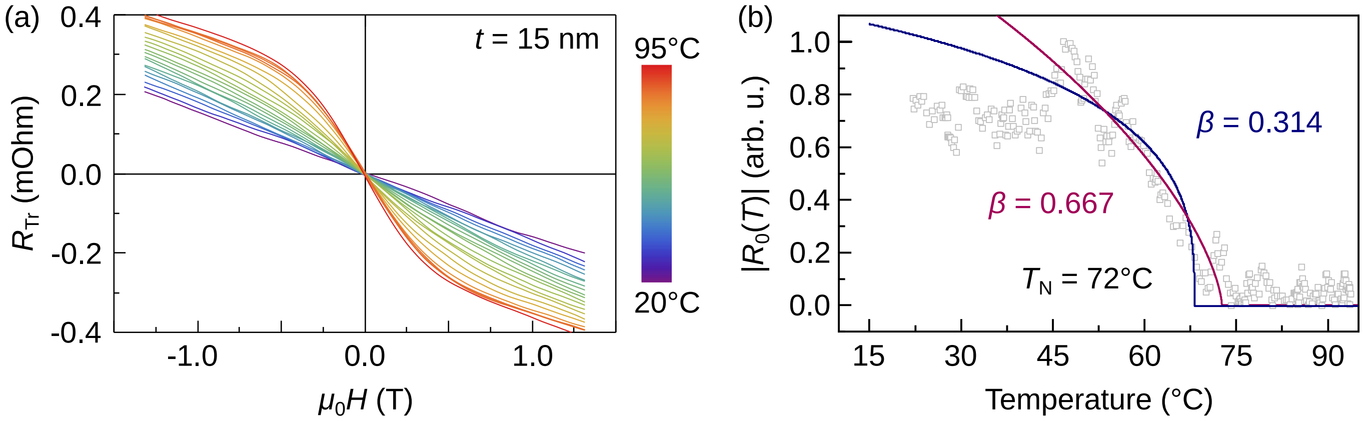

Supplementary Fig. 42. **Magnetotransport characterisation of 15-nm-thick Cr_2O_3 thin film.** (a) Transversal resistance *vs* magnetic field, $R_{\mathrm{Tr}}(H,T)$, curves are color coded according to the measurement temperature. (b) Normalized variation of the absolute values of $R_0(T)$ at $\mu_0 H = 1.25\,\mathrm{T}$ with temperature. Symbols correspond to the experimental data and lines correspond to the fit accordingly to the equation (S4) with the critical exponent β being equal to 0.314 (blue) and 0.667 (purple). We note that the fit with β being equal to 0.667 better fits the experimental data. Different critical behavior of this sample compared to samples with the thickness of larger than 30 nm suggests the onset of finite size effects.

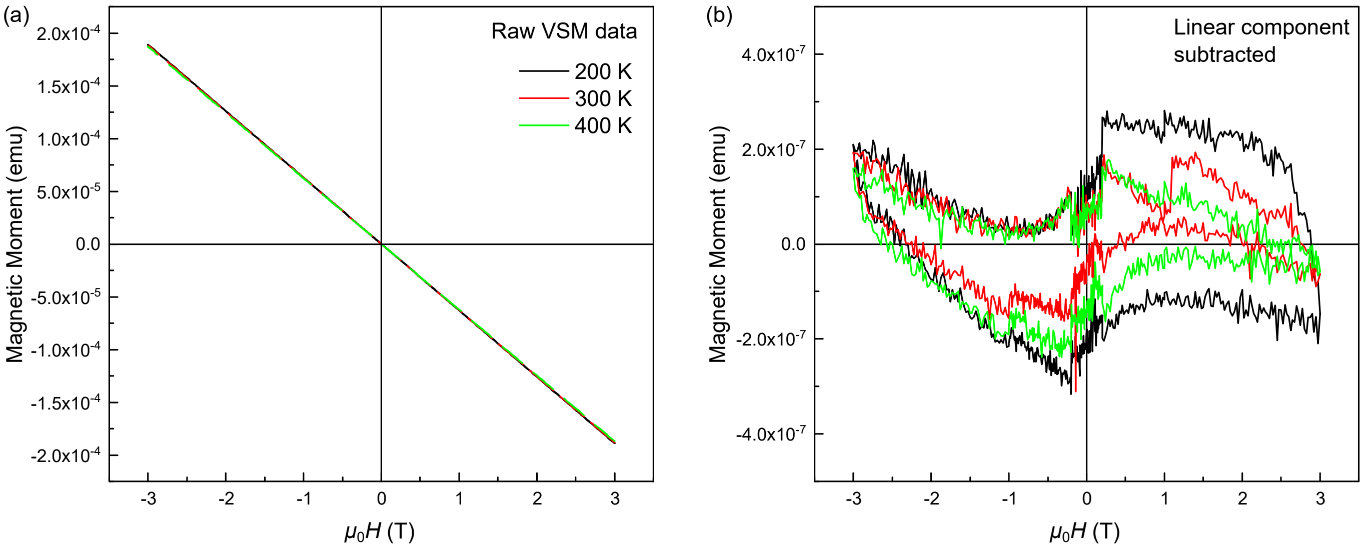

Supplementary Fig. 43. **Superconducting quantum interference device – vibrating sample magnetometry (SQUID-VSM) characterization of the 50-nm-thick Cr_2O_3 thin film.** (a) Magnetic hysteresis measured of the sample at different temperatures. The sample reveals linear change of magnetic moment with the applied magnetic field, which is expected for the diamagnetic Al_2O_3 substrate and antiferromagnetic (or paramagnetic above Néel temperature) Cr_2O_3 thin film. (b) Same as panel (a) but with subtracted linear contribution. The data reveal only a small variation of the magnetic moment below 0.2 μemu, which we consider as a detection limit of the device.

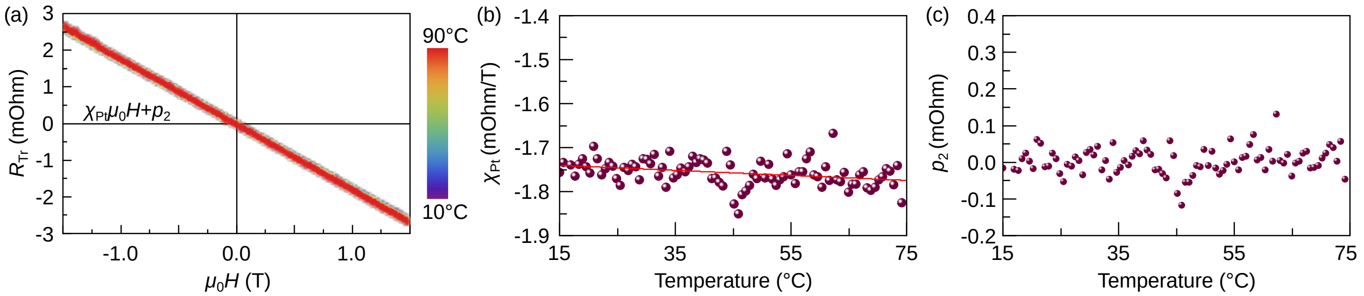

Supplementary Fig. 44. **Magnetotransport measurement of the Pt(5 nm) electrode deposited onto Al_2O_3 substrate**. (a) Transversal resistance *vs* magnetic field, $R_{Tr}(H,T)$, sweeps at different temperatures. (b,c) Temperature dependency of p_1 and p_2 coefficients of linear fit of $R_{Tr}(H,T)$ (S3). Linear temperature dependency indicates no magnetic transition in Pt electrode in measured temperature range.

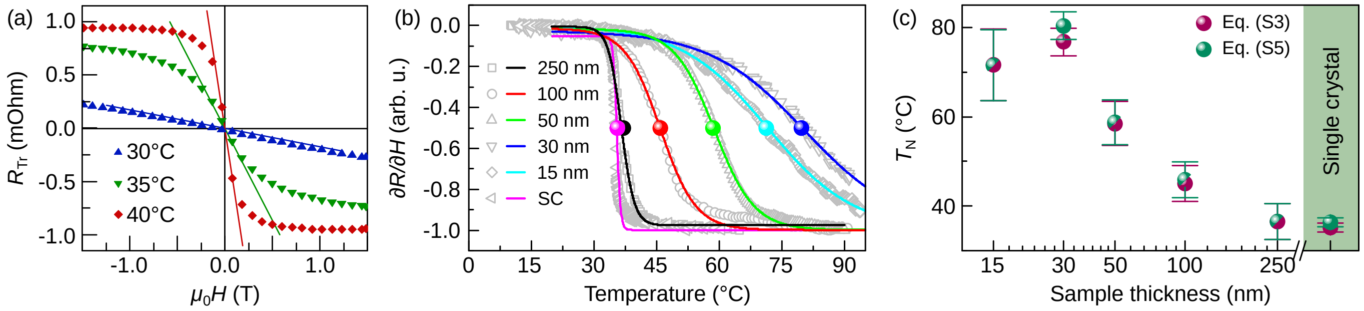

Supplementary Fig. 45. **Determination of the Néel temperature from magnetotransport measurements.** (a) $R_{\mathrm{Tr}}(H,T)$ sweeps at the selected temperatures for the 250-nm-thick Cr_2O_3 film. Symbols correspond to the measured data and lines represent the linear fit at low fields ($|\mu_0 H| < 100\,\mathrm{mT}$). (b) Temperature dependency of the linear slope of the data in the panel (a) and for other samples: symbols and lines correspond to data and fit accordingly to (S5), respectively. The transition temperature is determined from the inflection point on the fit data (highlighted with symbols). (c) Top surface Néel temperature $T_{\mathrm{N}}^{\mathrm{top}}$ evaluated according to Eq. (S4) and Eq. (S8). Error bars on the $T_{\mathrm{N}}^{\mathrm{top}}$ assessed by magnetotransport measurements arise from the smooth decay of the $|R_0(T)|$ to the noise level in the vicinity of AFM-PM transition.

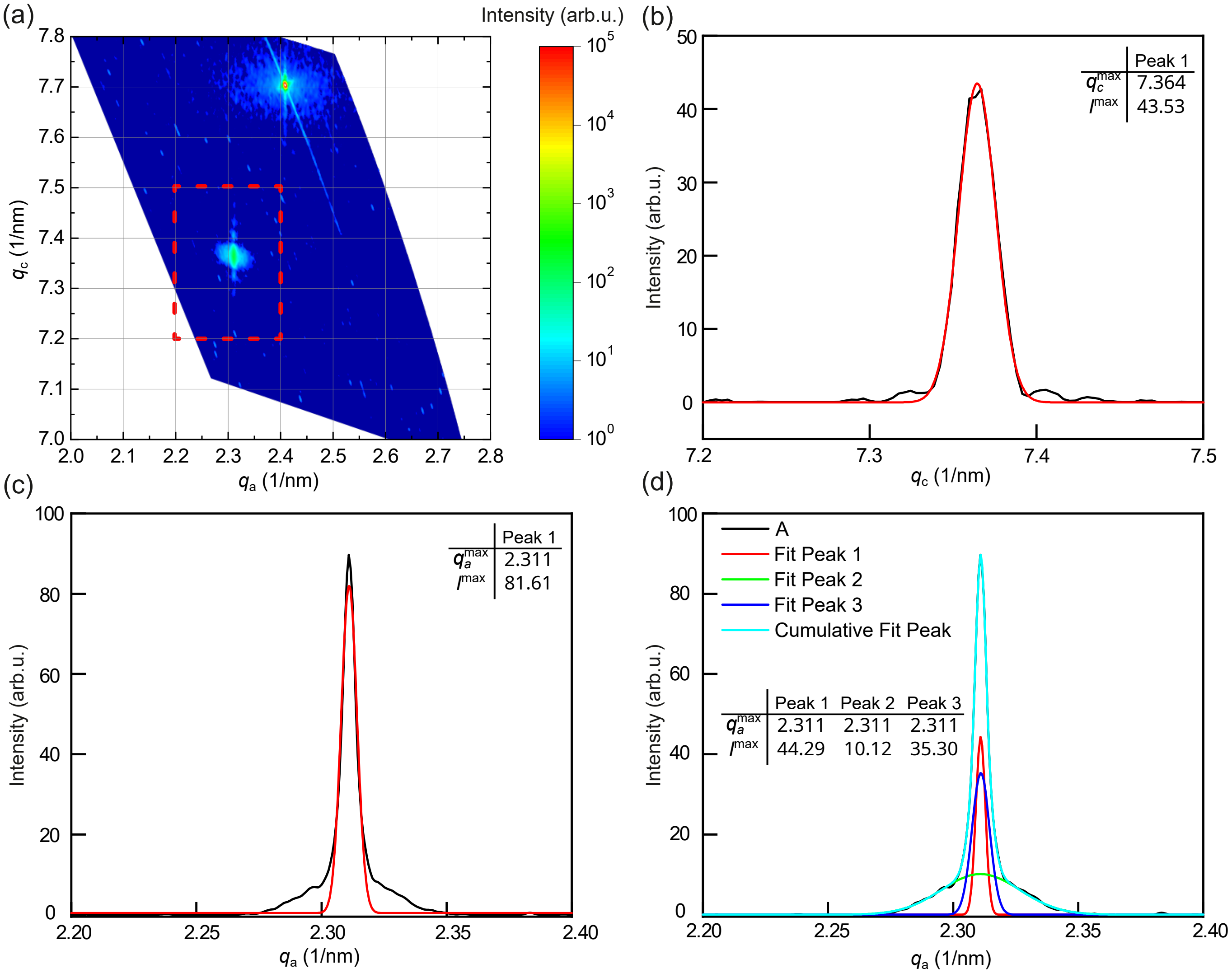

Supplementary Fig. 46. **Calculation of the average lattice parameters for the 30-nm-thick Cr_2O_3 film grown on a Pt(20 nm) seed layer.** (a) Experimental RSM data of the asymmetric $(1\,0\,\bar{1}\,10)$ reflection taken of Cr_2O_3 thin film with the thickness of 30 nm prepared on a 20-nm-thick Pt seed layer grown on the Al_2O_3 substrate. Line scans of the averaged intensity of the $(1\,0\,\bar{1}\,10)$ reflection along the q_c and q_a axes are shown in panels (b) and (c), respectively. The line scans are fitted using a single Gaussian function to extract the central position of the peak, which is taken as the average values of $q_{c,\mathrm{av}}$ and $q_{a,\mathrm{av}}$. The presence of shoulders on the line q_c is due to the incompete destructive interference on the thin film with smooth interfaces. We used triple peak Gaussian function to better fit the peak. We extracted three peak positions whose values are the same (2.311 nm^{-1}). The fit results (peak position and intensity) are shown as a table in each panel.

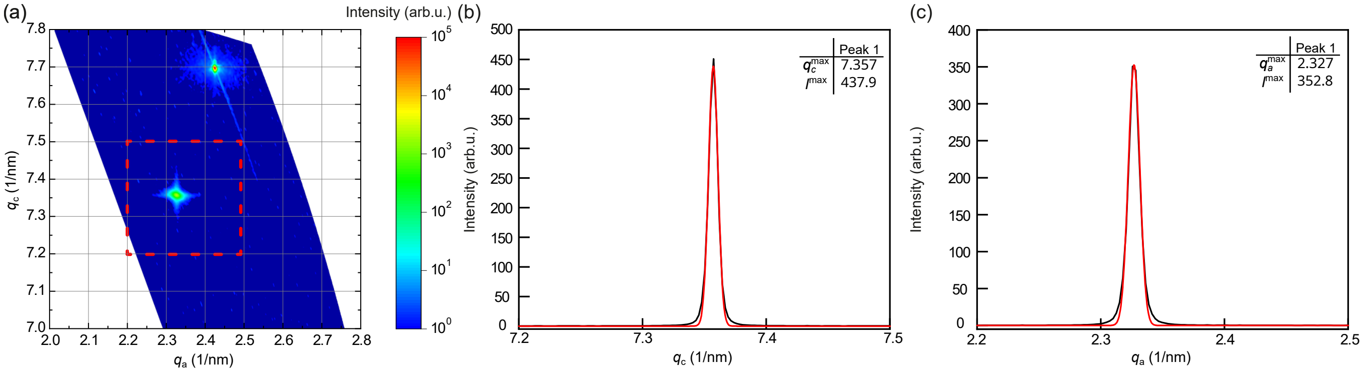

Supplementary Fig. 47. **Calculation of the average lattice parameters for the 250-nm-thick Cr_2O_3 film grown on Pt(20 nm).** (a) Experimental RSM data of the asymmetric $(1\,0\,\bar{1}\,10)$ reflection taken of Cr_2O_3 thin film with the thickness of 250 nm prepared on a 20-nm-thick Pt grown on Al_2O_3 substrate. Line scans of the averaged intensity of the $(1\,0\,\bar{1}\,10)$ reflection along the q_c and q_a axes are shown in panels (b) and (c), respectively. The line scans are fitted using a single Gaussian function to extract the central position of the peak, which is taken as the average values of $q_{c,\mathrm{av}}$ and $q_{a,\mathrm{av}}$. The fit results (peak position and intensity) are shown as a table in each panel.

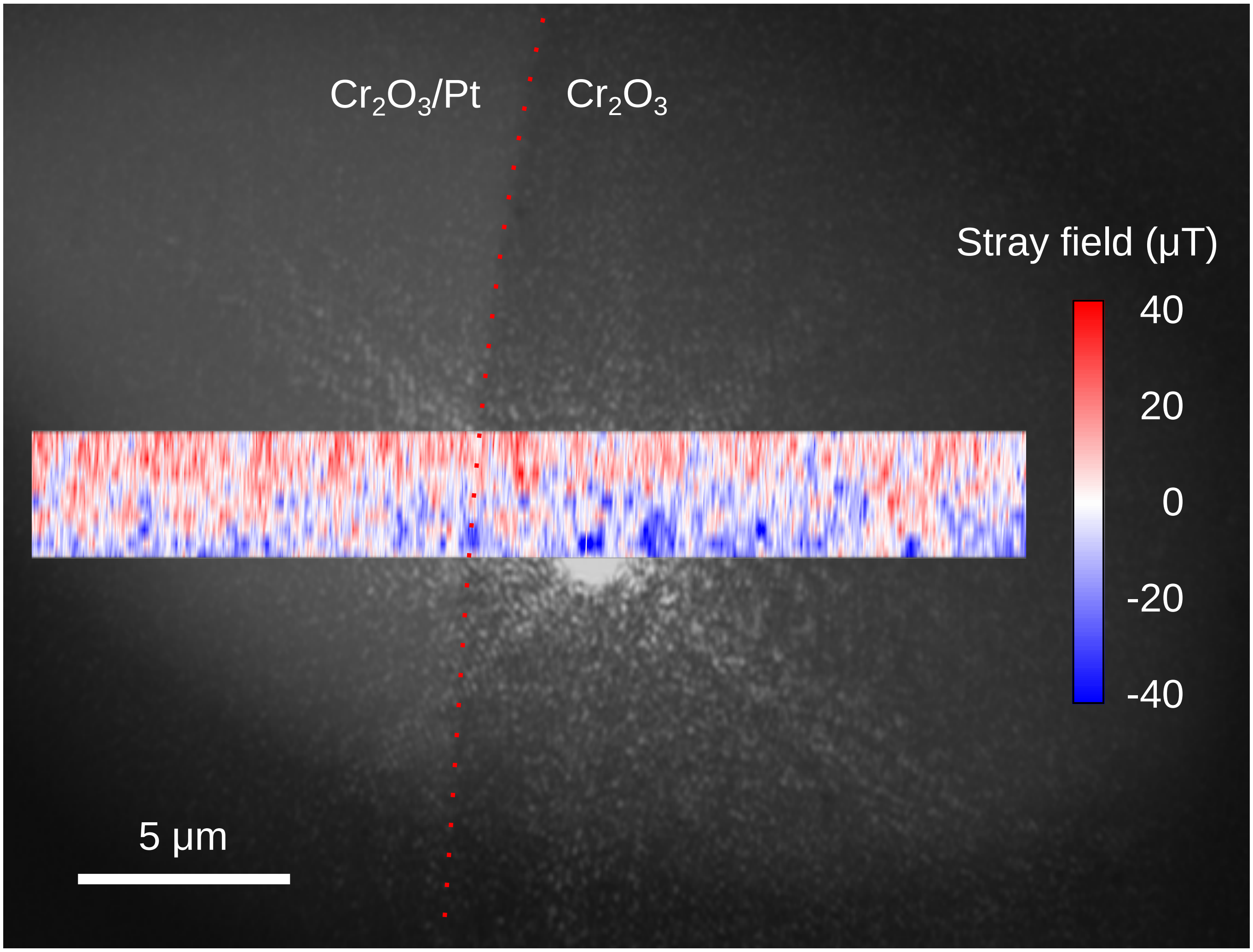

Supplementary Fig. 48. **Influence of a 3-nm-thick Pt electrode on the contrast of the antiferromagnetic domain pattern taken using scanning NV magnetometry.** The figure shows the optical photograph of a 50-nm-thick Cr_2O_3 thin film sample, which is partially covered with a Pt(3 nm) layer. The Pt layer is located at the left from the red dotted line. Inset shows the stray field map measured over both regions. We do not observe any change in the NV contrast when scanning over the region with and without Pt thin film.

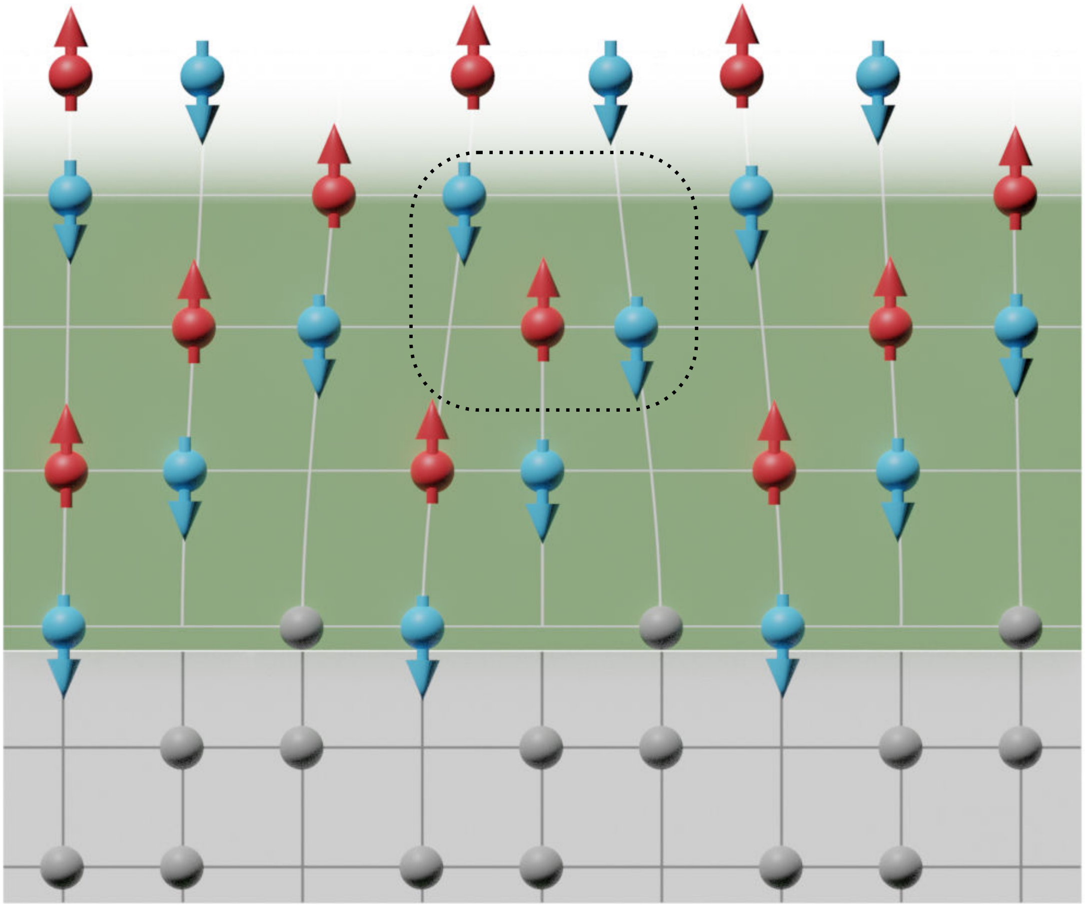

Supplementary Fig. 49. **Uncompensated magnetic moments at dislocations near the bottom Cr_2O_3 film surface.** The schematic shows how an uncompensated magnetic moment can arise in the vicinity of a dislocation (three moments of Cr ions giving this moment are highlighted by the dotted rectangle: the red arrow complemented to the blue one is absent). Here, antiferromagnetically coupled sublattices of Cr ions are shown by red and blue spheres with arrows and Al ions are shown by gray spheres. The first three atomic layers here preserve the Al_2O_3 structure and correspond to the pseudomorphic layer. Since the dislocations are mainly located near the bottom surface and their magnetization follows the Néel vector, they effectively contribute to the magnetization of the bottom film surface. The green and gray parts of the sample correspond to the Cr_2O_3 and Al_2O_3, respectively.

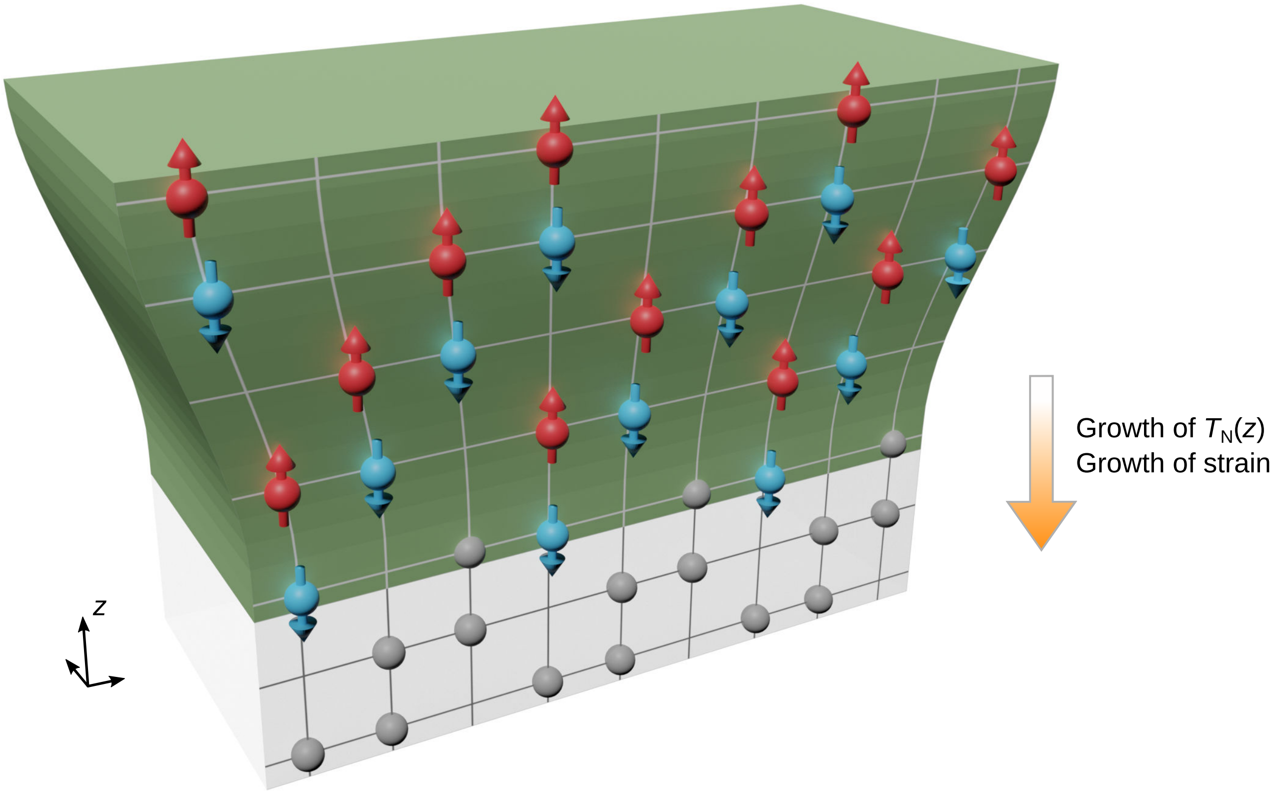

Supplementary Fig. 50. **Flexomagnetism of the inhomogeneously strained thin films.** The strain gradient leads to the emergent magnetization, whose direction is determined by the flexomagnetic tensor. In the particular case of the strain gradient along the direction perpendicular to the film surface, the film possesses a constant magnetization component of the flexomagnetic origin along z axis and the in-plane component aligned with one of the in-plane axes of crystal symmetry, see Eq. (S26). The green and gray parts of the sample correspond to the Cr_2O_3 and Al_2O_3, respectively.

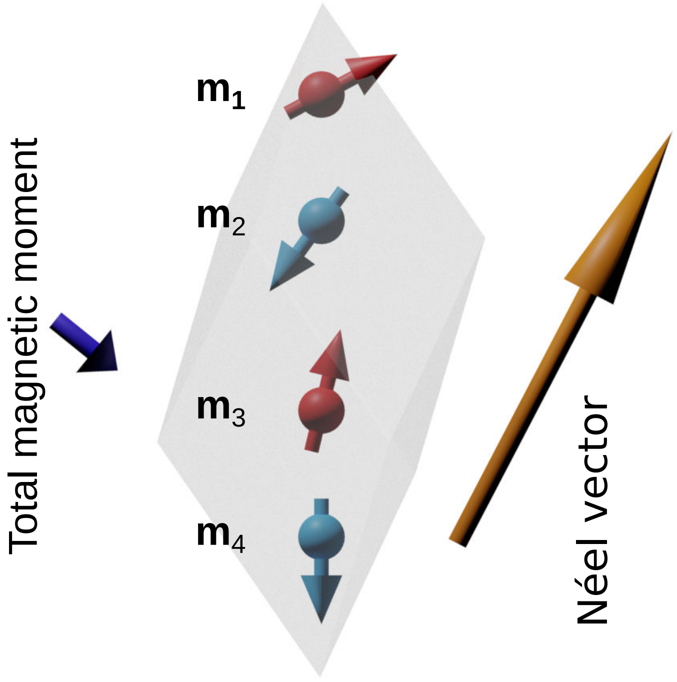

Supplementary Fig. 51. **The Dzyaloshinskii-like mechanism of the emergence of the magnetization at a non-collinear texture in Cr_2O_3 [60, 61].** The schematic shows a rhombohedral primitive cell of Cr_2O_3 with four Cr ions, whose magnetic moments are labeled by $\mathbf{m}_{1\ldots4}$. Two antiferromagnetic sublattices are highlighted by the arrows of red and light-blue color. A non-collinear texture leads to the continuous rotation of the neighboring magnetic moments. As a result, the atomistic Néel vector $\mathbf{l} = \mathbf{m}_1 - \mathbf{m}_2 + \mathbf{m}_3 - \mathbf{m}_4$ (orange arrow) is tilted in comparison with the direction of the moments shown here. The cell possesses an uncompensated magnetic moment $\mathbf{m} = \mathbf{m}_1 + \mathbf{m}_2 + \mathbf{m}_3 + \mathbf{m}_4$.

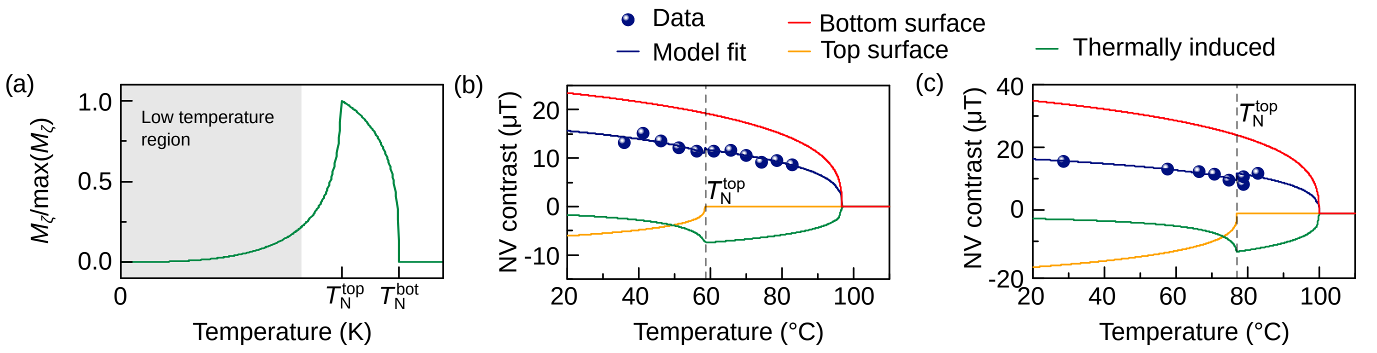

Supplementary Fig. 52. **Thermally induced magnetization in thin films.** (a) Magnetization induced by the thermal reduction of the sublattice magnetization along the film thickness reaches its maximum at $T = T_N^{top}$ when the top film surface becomes paramagnetic, and gradually approaches zero at $T = T_N^{bot}$ when the whole Cr_2O_3 film becomes paramagnetic. (b) Antiferromagnet-paramagnet phase transition for the 50-nm-thick film, and (c) for the 30-nm-thick film. In addition to the NV data and fit accordingly to Eq. (4, main text), individual components of the fit are shown.

Supplementary Tables

Supplementary Table 1. **Characterization of the structural defects in Cr_2O_3 films estimated from the PAS measurements.** Dislocations (dis.) and their complexes with mono-vacancies (dis.+V) are dominant defect types in the top part of the films.

Film thickness (nm)	τ_1 (ps)		τ_2 (ps)		Major defect type		Vacancy cluster size	
	top	interface	top	interface	top	interface	top	interface
250	176.8 ± 0.5	159 ± 2	312 ± 4	243 ± 3	dis.+V	dis.	~ 8	~ 4
100	184.3 ± 0.5	163 ± 1	334 ± 3	282 ± 2	dis.+V	dis.	~ 10	~ 6
50	184.7 ± 0.5	182.4 ± 0.6	355 ± 3	313 ± 5	dis.+V	dis.+V	~ 11	~ 8
30	192.6 ± 0.4	183.5 ± 0.5	377 ± 3	334 ± 4	dis.+V	dis.+V	>16	~ 10

Supplementary Table 2. **Estimations of the lattice parameters of Cr_2O_3 thin films based on the analysis of the RSM data.** The estimations are done for different regions of thin film samples. The region named as "Bottom" is closer to the Al_2O_3/Cr_2O_3 interface. The region named as "Top" is closer to the top surface of the Cr_2O_3 film. The region named as "Middle" is between regions indicated as "Bottom" and "Top". See also Fig. 1i,j of the main text. Confidence intervals for a and c lattice parameters are 0.03 Å and for c/a ratio 0.025.

Film thickness t, nm	Bottom			Middle			Top			Strain gradient, % nm^{-1}
	a, Å	c, Å	c/a	a, Å	c, Å	c/a	a, Å	c, Å	c/a	
30	4.75	14.08	2.96	4.8	13.85	2.88	4.88	13.75	2.82	0.18
50	4.75	14.08	2.96	4.83	13.81	2.86	4.92	13.7	2.79	0.13
100	4.93	13.88	2.81	–	–	–	4.93	13.67	2.77	0.015
250	–	–	–	4.95	13.63	2.76	–	–	–	–
Pt(20 nm)/250	–	–	–	4.96	13.59	2.74	–	–	–	–
Pt(20 nm)/30	–	–	–	4.96	13.59	2.74	–	–	–	–

Supplementary Table 3. **Contributions to the Néel temperature in films of different thickness.** The table summarizes measurements of the Néel temperature for the films of different thickness obtained via transport and NV magnetometry. The columns with a symbol δ are errors of the measurement. The column $T_{\mathrm{N}}^{\mathrm{top}}$ represents the lower estimate of the Néel temperature at the top surface obtained via transport measurements. The column $T_{\mathrm{N}}^{\mathrm{bot}}$ contains the estimate of the Néel temperature at the bottom surface. The contribution of the piezomagnetic effect $\Delta T_{\mathrm{piezo}}(\langle c/a\rangle)$ is estimated via the average strain [Eq. (S19)]. The contribution of the inhomogeneous strain distribution is listed in the column $\Delta T_{\zeta}(z=0)$ [Eq. (S18)]. We note that the NV data taken of the 30-nm-thick sample contains very limited number of data points above the $T = T_{\mathrm{N}}^{\mathrm{top}}$ because $T_{\mathrm{N}}^{\mathrm{top}}$ for this sample is close to the maximal achievable temperature in the NV magnetometer. Therefore, to assess the $T_{\mathrm{N}}^{\mathrm{bot}}$ for the 30-nm-thick sample, we used ζ determined for the 50-nm-thick sample.

Thickness t, nm	$T_{\mathrm{N}}^{\mathrm{top}}$, $^\circ$C	$\delta T_{\mathrm{N}}^{\mathrm{top}}$, $^\circ$C	$T_{\mathrm{N}}^{\mathrm{bot}}$, $^\circ$C	$\delta T_{\mathrm{N}}^{\mathrm{bot}}$, $^\circ$C	$\Delta T_{\mathrm{piezo}}$, $^\circ$C	$\Delta T_{\zeta}(0)$, $^\circ$C	ζ, $^\circ\mathrm{C\,nm^{-1}}$
30	77	+3	100	±6	25	23	0.76 ± 0.1
50	59	+5	97	±7	23	38.5	0.76 ± 0.1
100	45	+4	40.5	+4	6	0	0
250	35.5	+4	38.5	+5	4	0	0
Pt(20 nm)/30	30	+4	–	–	–	–	–
Pt(20 nm)/250	35	+4	–	–	–	–	–
Single crystal	35	+1	–	–	–	–	–

Supplementary Table 4. **Flexomagnetism of Cr_2O_3 thin films.** The table contains estimations for 30-nm-thick and 50-nm-thick films revealing flexomagnetism. The strain gradient is estimated accordingly to Eq. (S25). Then we use the value of the flexomagnetic coefficient (S23) to obtain magnetization. The surface magnetic moment is determined as the magnetization integrated over the film thickness. The average strain is estimated as $\langle u_{ij} \rangle = |\langle c/a \rangle - (c_0/a_0)|/(c_0/a_0)$ and is given for clarity.

Thickness t nm	Average strain %	Strain gradient $\langle \partial u_{ij}/\partial z \rangle$ $\%\,\mathrm{nm}^{-1}$	Magnetization $\mu_\mathrm{B}\,\mathrm{nm}^{-3}$	Surface magnetic moment $\mu_\mathrm{B}\,\mathrm{nm}^{-2}$
50	1.7	0.12	0.015 ± 0.001	0.72 ± 0.04
30	3.6	0.18	0.022 ± 0.001	0.65 ± 0.03

Supplementary Table 5. **Magnetization of Cr_2O_3 films.** The table summarizes the strength of different sources of magnetization, which can be measured with the given areal density of magnetic moment in units of μ_B/nm^2 (either present at the surface, or integrated over the thin film's thickness). Values in the table are given for the specific sample's temperature T. There are only low-temperature estimations for tensor components μ_{ijkl} and ν_{ijkl} in Eq. (S22b). For the flexomagnetic and Dzyaloshinskii-like sources of magnetization, the areal density of magnetic moment is given as the respective magnetization integrated over the film thickness. The column "Strain" indicates whether the strain is important for the given mechanism.

Source	Thickness, nm	Strain	Temperature	Value, $\mu_B\,\mathrm{nm}^{-2}$	Ref.
Uncompensated surface spins	200	–	$0.96T/T_N$	2.14 ± 1.5	[2]
	Single crystal	–	$0.98T/T_N$	2.2	[30]
	Single crystal	–	$0.96T/T_N$	2.3	[58]
Dislocations	200	✓	$0.95T/T_N$	0.455 ± 0.28	[1]
Flexomagnetism, Eq. (S26)	30	✓	$O(10^2\,\mathrm{K})$	0.65	This work
	50	✓	$O(10^2\,\mathrm{K})$	0.72	
Dzyaloshinskii-like [60, 61]	t	–	$\ll T_N$	$\sim 0.01t$	
Thermally induced, Eq. (S30)	$30\ldots50$	✓	T_N^{top}	~ 2	

References

[1] T. Kosub, M. Kopte, R. Hühne, P. Appel, B. Shields, P. Maletinsky, R. Hübner, M. O. Liedke, J. Fassbender, O. G. Schmidt, and D. Makarov, Nature Communications **8**, 13985 (2017).
[2] P. Appel, B. J. Shields, T. Kosub, N. Hedrich, R. Hübner, J. Faßbender, D. Makarov, and P. Maletinsky, Nano Letters **19**, 1682 (2019).
[3] M. Ichimura and J. Narayan, Philosophical Magazine A **72**, 297 (1995).
[4] J. Narayan, Acta Materialia **61**, 2703 (2013).
[5] M. Hÿtch, E. Snoeck, and R. Kilaas, Ultramicroscopy **74**, 131 (1998).
[6] GPA analysis was performed using FRWRtools plugin of Ch. Koch, https://www.physik.hu-berlin.de/en/sem/software/software_frwrtools.
[7] Y. Yang, X. Mao, Y. Yao, H. Huang, Y. Lu, L. Luo, X. Zhang, G. Yin, T. Yang, and X. Gao, Journal of Applied Physics **125**, 082508 (2019).
[8] S. Ryu, J. Y. Son, Y.-H. Shin, H. M. Jang, and J. F. Scott, Applied Physics Letters **95**, 242902 (2009).
[9] T. Yoshimoto, T. Goto, K. Shimada, B. Iwamoto, Y. Nakamura, H. Uchida, C. A. Ross, and M. Inoue, Advanced Electronic Materials **4**, 1800106 (2018).
[10] Y. Ge, O. Heczko, S.-P. Hannula, and S. Fähler, Acta Materialia **58**, 6665 (2010).
[11] "Cr_2O_3 crystal structure: Datasheet from "PAULING FILE multinaries edition – 2012" in Springermaterials (https://materials.springer.com/isp/crystallographic/docs/sd_0541813)," Copyright 2016 Springer-Verlag Berlin Heidelberg & Material Phases Data System (MPDS), Switzerland & National Institute for Materials Science (NIMS), Japan.
[12] M. O. Liedke, W. Anwand, R. Bali, S. Cornelius, M. Butterling, T. T. Trinh, A. Wagner, S. Salamon, D. Walecki, A. Smekhova, H. Wende, and K. Potzger, Journal of Applied Physics **117**, 163908 (2015).
[13] W. Anwand, G. Brauer, M. Butterling, H. R. Kissener, and A. Wagner, Defect and Diffusion Forum **331**, 25 (2012).
[14] R. Krause-Rehberg and H. S. Leipner, *Positron Annihilation in Semiconductors: Defect Studies*, Springer Series in Solid-State Sciences, Vol. 127 (Springer Berlin Heidelberg, 1999).
[15] A. Wagner, M. Butterling, M. O. Liedke, K. Potzger, and R. Krause-Rehberg (Author(s), 2018).
[16] J. V. Olsen, P. Kirkegaard, N. J. Pedersen, and M. Eldrup, physica status solidi (c) **4**, 4004 (2007).
[17] F. Tuomisto and I. Makkonen, Reviews of Modern Physics **85**, 1583 (2013).
[18] M. J. Puska and R. M. Nieminen, Reviews of Modern Physics **66**, 841 (1994).
[19] M. Alatalo, B. Barbiellini, M. Hakala, H. Kauppinen, T. Korhonen, M. J. Puska, K. Saarinen, P. Hautojärvi, and R. M. Nieminen, Physical Review B **54**, 2397 (1996).
[20] S. Assali, M. Elsayed, J. Nicolas, M. O. Liedke, A. Wagner, M. Butterling, R. Krause-Rehberg, and O. Moutanabbir, Applied Physics Letters **114**, 251907 (2019).
[21] J. Čížek, O. Melikhova, Z. Barnovská, I. Procházka, and R. K. Islamgaliev, Journal of Physics: Conference Series **443**, 012008 (2013).
[22] J. Mougin, N. Rosman, G. Lucazeau, and A. Galerie, Journal of Raman Spectroscopy **32**, 739 (2001), https://analyticalsciencejournals.onlinelibrary.wiley.com/doi/pdf/10.1002/jrs.734.
[23] K. Iishi, Physics and Chemistry of Minerals **3**, 1 (1978).
[24] S.-H. Shim, T. S. Duffy, R. Jeanloz, C.-S. Yoo, and V. Iota, Physical Review B **69** (2004), 10.1103/physrevb.69.144107.
[25] S. Foner, Physical Review **130**, 183 (1963).
[26] B. A. Ivanov, Low Temperature Physics **31**, 635 (2005).
[27] T. Iino, T. Moriyama, H. Iwaki, H. Aono, Y. Shiratsuchi, and T. Ono, Applied Physics Letters **114**, 022402 (2019).
[28] A. Hoser and U. Köbler, *Renormalization Group Theory* (Springer Berlin Heidelberg, 2012).
[29] I. Veremchuk, P. Makushko, N. Hedrich, Y. Zabila, T. Kosub, M. O. Liedke, M. Butterling, A. G. Attallah, A. Wagner, U. Burkhardt, O. V. Pylypovskyi, R. Hübner, J. Fassbender, P. Maletinsky, and D. Makarov, ACS Applied Electronic Materials (2022), 10.1021/acsaelm.2c00398.
[30] N. Hedrich, K. Wagner, O. V. Pylypovskyi, B. J. Shields, T. Kosub, D. D. Sheka, D. Makarov, and P. Maletinsky, Nature Physics **17**, 574 (2021).
[31] S. Sahoo and C. Binek, Philosophical Magazine Letters **87**, 259 (2007).
[32] P. J. van der Zaag, Y. Ijiri, J. A. Borchers, L. F. Feiner, R. M. Wolf, J. M. Gaines, R. W. Erwin, and M. A. Verheijen, Physical Review Letters **84**, 6102 (2000).
[33] A. Mandziak, G. D. Soria, J. E. Prieto, P. Prieto, C. Granados-Miralles, A. Quesada, M. Foerster, L. Aballe, and J. de la Figuera, Scientific Reports **9**, 13584 (2019).
[34] X. He, W. Echtenkamp, and C. Binek, Ferroelectrics **426**, 81 (2012).
[35] W. Yuan, Q. Zhu, T. Su, Y. Yao, W. Xing, Y. Chen, Y. Ma, X. Lin, J. Shi, R. Shindou, X. C. Xie, and W. Han, Science Advances **4**, eaat1098 (2018).
[36] W. Yuan, T. Su, Q. Song, W. Xing, Y. Chen, T. Wang, Z. Zhang, X. Ma, P. Gao, J. Shi, and W. Han, Scientific Reports **6** (2016), 10.1038/srep28397.
[37] R. Schlitz, T. Kosub, A. Thomas, S. Fabretti, K. Nielsch, D. Makarov, and S. T. B. Goennenwein, Applied Physics Letters

112, 132401 (2018).
[38] A. Mahmood, W. Echtenkamp, M. Street, J.-L. Wang, S. Cao, T. Komesu, P. A. Dowben, P. Buragohain, H. Lu, A. Gruverman, A. Parthasarathy, S. Rakheja, and C. Binek, Nature Communications **12** (2021), 10.1038/s41467-021-21872-3.
[39] S. Mu and K. D. Belashchenko, Physical Review Materials **3**, 034405 (2019).
[40] R. A. Buckingham and J. E. Lennard-Jones, Proceedings of the Royal Society of London. Series A. Mathematical and Physical Sciences **168**, 264 (1938).
[41] Y. K. Shin, H. Kwak, A. V. Vasenkov, D. Sengupta, and A. C. van Duin, ACS Catalysis **5**, 7226 (2015).
[42] I. F. Galván, M. Vacher, A. Alavi, C. Angeli, F. Aquilante, J. Autschbach, J. J. Bao, S. I. Bokarev, N. A. Bogdanov, R. K. Carlson, L. F. Chibotaru, J. Creutzberg, N. Dattani, M. G. Delcey, S. S. Dong, A. Dreuw, L. Freitag, L. M. Frutos, L. Gagliardi, F. Gendron, A. Giussani, L. González, G. Grell, M. Guo, C. E. Hoyer, M. Johansson, S. Keller, S. Knecht, G. Kovačević, E. Källman, G. L. Manni, M. Lundberg, Y. Ma, S. Mai, J. P. Malhado, P. Å. Malmqvist, P. Marquetand, S. A. Mewes, J. Norell, M. Olivucci, M. Oppel, Q. M. Phung, K. Pierloot, F. Plasser, M. Reiher, A. M. Sand, I. Schapiro, P. Sharma, C. J. Stein, L. K. Sørensen, D. G. Truhlar, M. Ugandi, L. Ungur, A. Valentini, S. Vancoillie, V. Veryazov, O. Weser, T. A. Wesołowski, P.-O. Widmark, S. Wouters, A. Zech, J. P. Zobel, and R. Lindh, Journal of Chemical Theory and Computation **15**, 5925 (2019).
[43] S. Plimpton, Journal of Computational Physics **117**, 1 (1995).
[44] T.-T. Chang, D. Foster, and A. H. Kahn, Journal of Research of the National Bureau of Standards **83**, 133 (1978).
[45] M. Tachiki and T. Nagamiya, Journal of the Physical Society of Japan **13**, 452 (1958), https://doi.org/10.1143/JPSJ.13.452.
[46] P. Lukashev and R. F. Sabirianov, Physical Review B **82**, 094417 (2010).
[47] H. Klein, U. Scherz, M. Schulz, H. Setyono, and K. Wisznewski, Zeitschrift für Physik B Condensed Matter **28**, 149 (1977).
[48] L. D. Landau, L. P. Pitaevskii, and E. M. Lifshitz, *Electrodynamics of Continuous Media* (Elsevier Science & Technology, 1984).
[49] A. A. Bukharaev, A. K. Zvezdin, A. P. Pyatakov, and Y. K. Fetisov, Physics-Uspekhi **61**, 1175 (2018).
[50] Y. Kota, H. Imamura, and M. Sasaki, Applied Physics Express **6**, 113007 (2013).
[51] Y. Kota, Y. Yoshimori, H. Imamura, and T. Kimura, Applied Physics Letters **110**, 042902 (2017).
[52] G. Gorodetsky, R. M. Hornreich, and S. Shtrikman, Physical Review Letters **31**, 938 (1973).
[53] C. A. Brown, "Magnetoelectric domains in single crystal chromium oxide," (1969).
[54] J. Matthews and J. Crawford, Thin Solid Films **5**, 187 (1970).
[55] C. Chappert and P. Bruno, Journal of Applied Physics **64**, 5736 (1988).
[56] J. Fassbender, U. May, B. Schirmer, R. M. Jungblut, B. Hillebrands, and G. Güntherodt, Physical Review Letters **75**, 4476 (1995).
[57] W. Weber, A. Bischof, R. Allenspach, C. H. Back, J. Fassbender, U. May, B. Schirmer, R. M. Jungblut, G. Güntherodt, and B. Hillebrands, Physical Review B **54**, 4075 (1996).
[58] M. S. Wörnle, P. Welter, M. Giraldo, T. Lottermoser, M. Fiebig, P. Gambardella, and C. L. Degen, Physical Review B **103**, 094426 (2021).
[59] A. F. Andreev and V. I. Marchenko, Sov. Phys. Usp. **23**, 21 (1980).
[60] A. F. Andreev, Journal of Experimental and Theoretical Physics Letters **63**, 758 (1996).
[61] A. F. Kabychenkov and F. V. Lisovskii, Technical Physics **64**, 980 (2019).
[62] E. A. Eliseev, M. D. Glinchuk, V. Khist, V. V. Skorokhod, R. Blinc, and A. N. Morozovska, Physical Review B **84**, 174112 (2011).
[63] Y. Kota and H. Imamura, Applied Physics Express **10**, 013002 (2016).
[64] (Springer-Verlag) pp. 1–6.
[65] N. Papanicolaou, Physical Review B **51**, 15062 (1995).
[66] E. G. Tveten, T. Müller, J. Linder, and A. Brataas, Physical Review B **93**, 104408 (2016).
[67] M. S. Wörnle, *Nanoscale Scanning Diamond Magnetometry of Antiferromagnets*, Ph.D. thesis (2021).
